# Stereoselective Synthesis of the Di-Spirooxindole Analogs Based Oxindole and Cyclohexanone Moieties as Potential Anticancer Agents

**DOI:** 10.3390/molecules26206305

**Published:** 2021-10-19

**Authors:** Abdullah Mohammed Al-Majid, M. Ali, Mohammad Shahidul Islam, Saeed Alshahrani, Abdullah Saleh Alamary, Sammer Yousuf, M. Iqbal Choudhary, Assem Barakat

**Affiliations:** 1 Department of Chemistry, College of Science, King Saud University, P.O. Box 2455, Riyadh 11451, Saudi Arabia; amajid@ksu.edu.sa (A.M.A.-M.); maly.c@ksu.edu.sa (M.A.); mislam@ksu.edu.sa (M.S.I.); chemistry99y@gmail.com (S.A.); alamary1401@yahoo.com (A.S.A.); 2 International Center for Chemical and Biological Sciences, H.E.J. Research Institute of Chemistry, University of Karachi, Karachi 75270, Pakistan; dr.sammer.yousuf@gmail.com (S.Y.); iqbal.choudhary@iccs.edu (M.I.C.); 3 Department of Chemistry, Faculty of Science, Alexandria University, P.O. Box 426, Ibrahimia, Alexandria 21321, Egypt

**Keywords:** spirooxindole, azomethine ylides, [3+2] cycloaddition reaction, anti-cancer activity

## Abstract

A new series of di-spirooxindole analogs, engrafted with oxindole and cyclohexanone moieties, were synthesized. Initially, azomethine ylides were generated via reaction of the substituted isatins **3a–f** (isatin, **3a**, 6-chloroisatin, **3b**, 5-fluoroisatin, **3c**, 5-nitroisatin, **3d**, 5-methoxyisatin, **3e**, and 5-methylisatin, **3f**, and (2*S*)-octahydro-1*H*-indole-2-carboxylic acid **2**, in situ azomethine ylides reacted with the cyclohexanone based-chalcone **1a–f** to afford the target di-spirooxindole compounds **4a–n**. This one-pot method provided diverse structurally complex molecules, with biologically relevant spirocycles in a good yields. All synthesized di-spirooxindole analogs, engrafted with oxindole and cyclohexanone moieties, were evaluated for their anticancer activity against four cancer cell lines, including prostate PC3, cervical HeLa, and breast (MCF-7, and MDA-MB231) cancer cell lines. The cytotoxicity of these di-spirooxindole analogs was also examined against human fibroblast BJ cell lines, and they appeared to be non-cytotoxic. Compound **4b** was identified as the most active member of this series against prostate cancer cell line PC3 (IC_50_ = 3.7 ± 1.0 µM). The cyclohexanone engrafted di-spirooxindole analogs **4a** and **4l** (IC_50_ = 7.1 ± 0.2, and 7.2 ± 0.5 µM, respectively) were active against HeLa cancer cells, whereas NO_2_ substituted isatin ring and *meta*-fluoro-substituted (2*E*,6*E*)-2,6-dibenzylidenecyclohexanone containing **4i** (IC_50_ = 7.63 ± 0.08 µM) appeared to be a promising agent against the triple negative breast cancer MDA-MB231 cell line. To explore the plausible mechanism of anticancer activity of di-spirooxindole analogs, molecular docking studies were investigated which suggested that spirooxindole analogs potentially inhibit the activity of MDM2.

## 1. Introduction

According to GLOBOCAN report in 2018, there were 9.6 million mortalities and 18 million new cases of cancer. In addition, the report describes cancer as the second leading cause of death worldwide. Therefore, development of anticancer agents with low toxicity, high efficacy, low drug resistance, and acceptable bioavailability is an urgent need to meet this global health challenge [1]. Drug discovery based on medicinal chemistry of natural and synthetic products have gained much attention [2,3]. In the past century, there has been an enormous success in drug innovation in the area of oral availability and biological compatibility, but drug resistance has emerged as a challenge for the medicinal chemists. Therefore, pharmacologists and chemists are focussing on functional diversity of drug leads [4], nano-formulation, and drug delivery development [5,6,7], with an aim of overcoming the existing problems. Specifically, the alkaloids spirooxindole scaffold, as a member of the oxindole class of natural products [8] has received much attention. The first member of this series was isolated from Apocynaceae and Rubiaceae plants. The spirooxindole scaffold is a privileged structure consisting of two basic sub-units: the first is oxindole with multiple functionalities, which can interact as acceptors or donors with the biological targets via hydrogen bonding. The second unit is a carbocyclic or heterocyclic moiety fused with oxindole ring at the C-3 position. It provides an opportunity to regulate many physicochemical properties and the liposolubility of spirooxindoles [9]. Accordingly, the significant biological activities (e.g., anti-inflammatory, anticancer, analgesic, antimicrobial, antimalarial, antioxidant, antiviral, antidiabetic, antiatherosclerotic, and insecticidal properties) and unique spatial architecture of spirooxindoles have attracted a remarkable attention of pharmacologists and chemists [10,11]. In the last few decades, several approaches towards new of spirooxindole analogs with structural diversity have been explored [12]. Based on the literature survey, the spirooxindole scaffold has shown to be a promising candidate for anti-cancer drug discovery.

**Figure 1:**
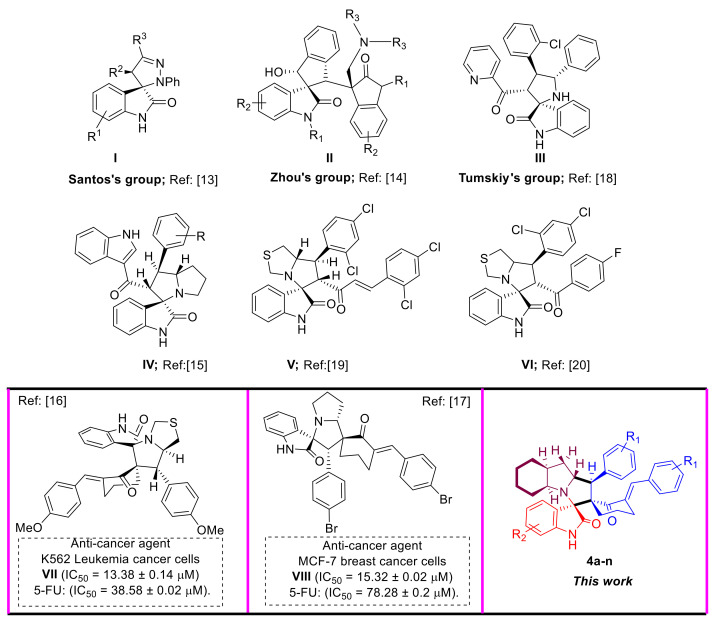
The anticancer activity of some reported spirooxindoles analogs.

In 2014, Santos’s group reported the synthesis of some novel spiropyrazoline based oxindole scaffold, and subsequently examined the cytotoxicity in vitro toward MCF-7 breast cancer cell line (Figure 1). The hit with high efficacy towards the breast cancer line was checked against MDA-MB-231 cell line. The results demonstrated that compound **I** exhibited a high activity with higher selectivity between the two cell lines with GI_50_ < 7.4 μM and >10-fold than the MDA-MB-231 cell line. Interestingly, the promising compound **I** behaved safely, and was noncytotoxic to HEK293T normal cell line [13].

Zhou’s group synthesized a family of spirooxindoles **II**, grafted with five-membered carbocyclic, with substituted oxindoles via set of chemical transformations, including Knoevenagel condensation/Michael cyclization (Figure 1). The synthesized compounds were evaluated for anticancer activity against three cancer cell lines, lung A549, human leukemia K562, and prostate-cancer cell lines PC-3. From this study, some representative compounds showed a comparable or stronger inhibitory effect against human leukemia cells K562 (IC_50_ = 7.4 to 32.8 μM) as compared to cisplatin (up to 3.4-fold). Moreover, other hits have either shown equivalent inhibition toward A549 cell line or slightly increased inhibitory activity against prostate cancer PC-3 cell line as compared to cisplatin [14]. 

In 2018, Barakat et al. reported a family of functionalized spirooxindoles. Representative compounds having the substituent on the benzene ring, such as 4-Me, 3-Br, and 4-CF_3_, exhibited potent cytotoxic activity against HCT-116 cells (IC_50_ = 7 μM, 9 μM, and 9 μM, respectively) and high selectivity index (SI) toward normal cells (SI > 2) [15]. On the other hand, the research group reported that the 2,4-diCl substituted benzene showed high cytotoxic activity and selectivity against PC-3 and HepG2 with IC_50_ = 2 μM for both cancer cells (SI = 4.5 and 4.5, respectively), and was more potent as compared to positive control, such as cisplatin (IC_50_ = 5 μM and 5.5 μM, respectively). In our previous reports, we explored the anticancer activity of the spriooxindole scaffold-based cyclohexanone against two cancer cell lines, MCF-7 breast and K562-leukemia cancer cells. We found that the compound **VII**, with a 4-methoxy group substituted benzene, was the most potent compound (IC_50_ = 13.38 ± 0.14 μM), targeting K562 leukemia cancer cells more selectively than 5-FU (IC_50_ = 38.58 ± 0.02 μM) Additionally, we noted that the hit with the *p*-Br-substituted benzene **VIII** had a higher efficacy for targeting MCF-7 breast cancer cells (IC_50_ = 15.32 ± 0.02 μM) as compared to the standard drug, 5-fluorouracil (5-FU) (IC_50_ = 78.28 ± 0.2 μM) (Figure 1) [16,17]. 

In 2019, Tumskiy et al. synthesized five spirooxindolepyrrolidines and examined the cytotoxic activity against some cell lines (Vero normal and HeLa cancer cells). The results demonstrated that hit **III** having a pyridine moiety with the chlorine atom in the *ortho* position exhibited a moderate selectivity (3-fold) between HeLa cancer cells and Vero healthy cells [18].

In continuation of research work, Barakat et al. reported the synthesis of spirooxindole–pyrrolothiazoles having a 3-cinnamoyl moiety. The results of cytotoxicity activities assay disclosed that compound **V** was the most active member of the series towards HCT-116, HepG2, and PC-3 cancer cells (IC_50_ < 4 μM). The selectivity index for the cancer cells versus the normal cells was superior to 2. Additionally, the research group carried out a set of biological assays which indicated that compound **V** could inhibit cell migration, colony formation, arrest cancer cell growth at the G2/M phase and induce apoptosis through extrinsic and intrinsic pathways [19] (Figure 1). 

In 2019, Barakat et al. reported the synthesis and cytotoxicity activities (HeLa) of the hit depicted in Figure 1 (i.e., **VI**). The antiproliferative assay showed that the compound can inhibit the proliferation of HeLa cancer cell line (IC_50_ = 11.2 μM), but less than the anticancer drug, doxorubicin (IC_50_ = 1.2 μM) [20].

The large library of the spirooxindole scaffold was generated with diverse pharmaceutical activities including low toxicity, acceptable bioavailability, and high efficiency [21,22,23,24,25,26,27,28,29]. In this paper, we describe in detail the synthesis of the spirooxindole analogs with significant bioactivities against the cancer cell in vitro. Molecular docking studies were also carried out to explore the plausible mechanism of anticancer activity of di-spirooxindole analogs.

## 2. Results and Discussion

### 2.1. Synthesis of *(**4a–n**)*

**Scheme 1:**
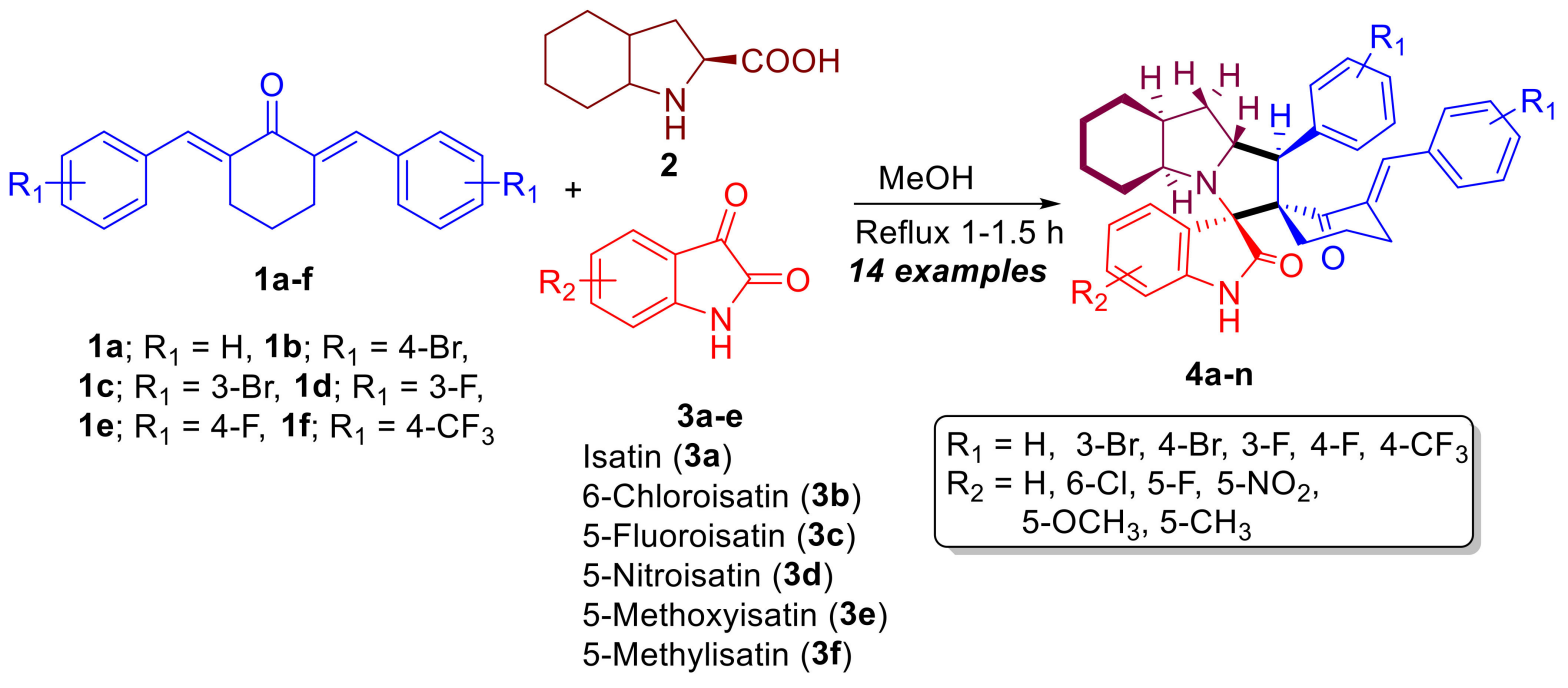
Enantio-selective synthesis of the di-spirooxindoles **4a–n**-based oxindole and cyclohexanone moieties.

**Scheme 2:**
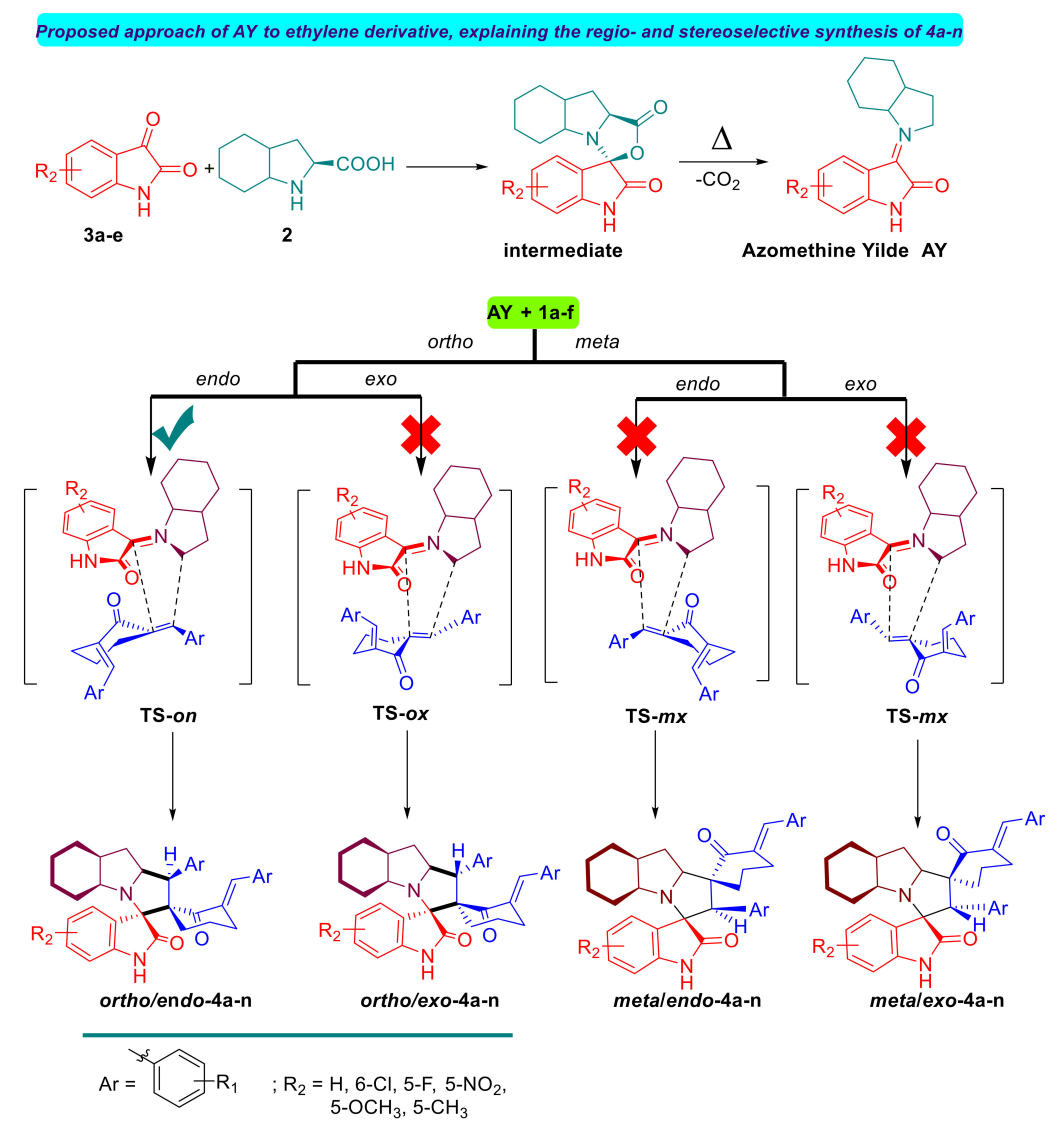
Plausible approach of **AY** to ethylene derivative, explaining the regio- and stereoselective synthesis of the target compounds **4a–n.**

The main objective of this study was to synthesize a new series of dispirooxindole scaffold and to examine their anticancer activity. For this purpose, desired dispirooxindole derivatives (**4a–n**) were synthesized *via* one-pot multicomponent reaction approach proceeded through [3+2] cycloaddition reaction (Scheme 1), and the plausible mechanism described in Scheme 2 [17,30,31,32,33,34,35]. Fourteen members with different electronic effects were prepared according to our previously reported protocol. The reaction proceeded smoothly via mixing the *α*,*β*-unsaturated ketones (**1a–f**), with the substituted isatin (**3a–f**) (Isatin (**3a**), 6-chloroisatin (**3b**), 5-fluoroisatin (**3c)**, 5-nitroisatin (**3d**), 5-methoxyisatin (**3e**), and 5-methylisatin (**3f**), and (2*S*)-octahydro-1*H*-indole-2-carboxylic acid (**2**) in MeOH under reflux conditions at 60 °C for 1–1.5 h to afford the requisite di-spirooxindoles in high chemical yields (up to 75%) with regio- and diastereoselective manner. This led to the generation of indoline analogs which are isosteric derivatives of previously synthesized compounds [12,13]. The aromatic substituents (R_1_ = H, F, Br, and CF_3_) in the cyclohexanone based-chalcones were employed in the [3+2]-dipolar cycloaddition reaction approach [31,32,33,34,35]. The chemical structures were deduced on the basis of spectroscopic techniques, including NMR, IR, and MS as well as CHN elemental analysis. Optical rotation of the synthesized compounds were carried out and confirmed the optical purity of the dispirooxindoles (**4a–n**).

**Figure 2:**
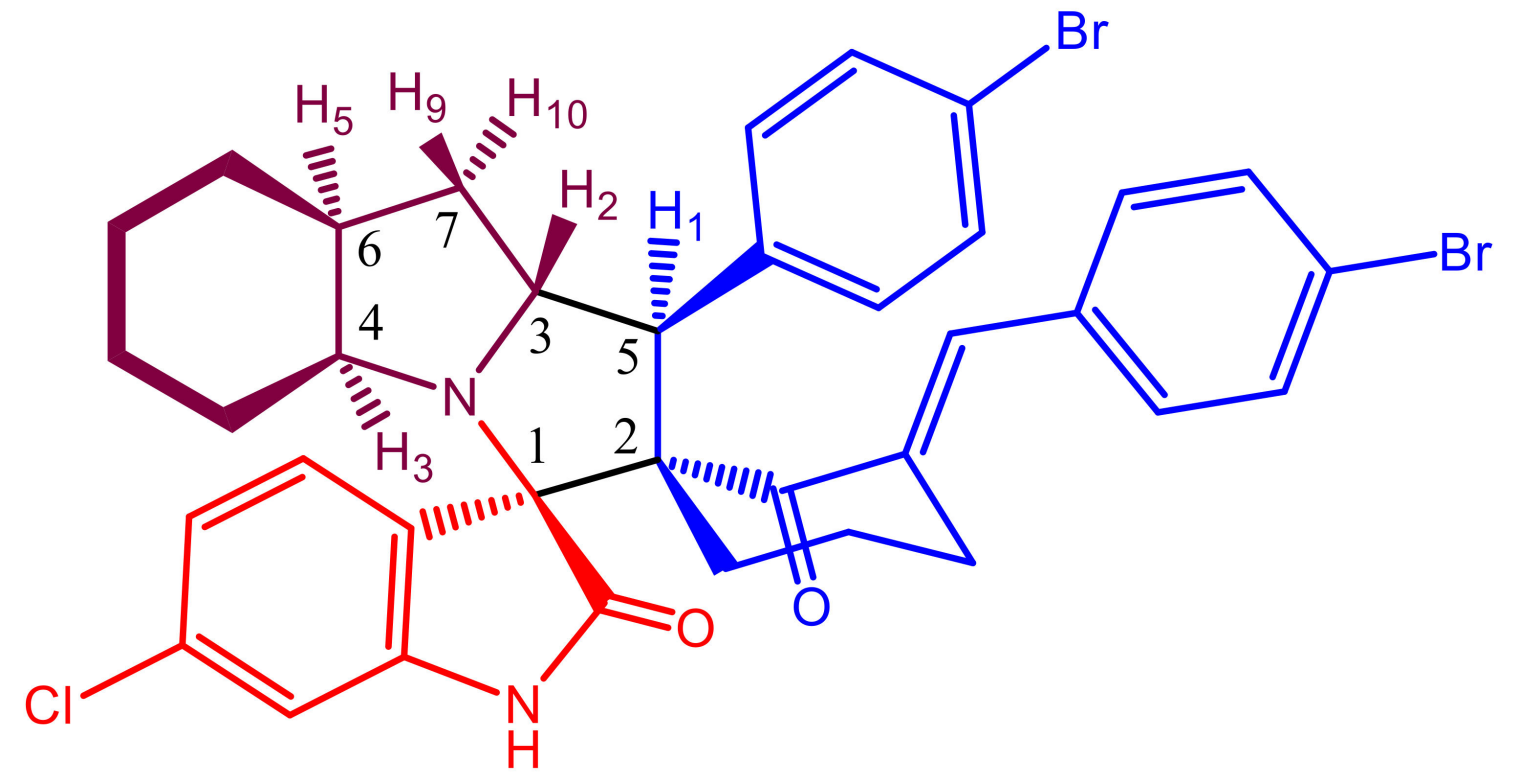
Chemical structure of **4f**.

To assign the stereochemistry of the final compounds, several attempts were carried out to obtain a suitable single crystal for X-ray diffraction analysis but unfortunately, it failed as it provided amorphous materials. Then, extensive analysis by 1D- and 2D-NMR spectroscopy were investigated (Supplementary Materials Figures S15–S19). Compound **4f** was chosen as a model example to reach the stereochemistry assignment of the final compounds (Figure 2). As shown in Supplementary Materials Figure S15, ^1^H- and ^13^C-NMR confirmed the only desired regioisomer which confirm the proposed [3+2] cycloaddition reaction approach (*ortho/endo*-Pathway) [31,32,33,34,35] (Scheme 2). 

It was clearly evident from the ^13^C- and DEPT-135 spectra by comparison that the molecule **4f** having 4 CH carbon (C_3_, C_4,_ C_5_, and C_6_) at *δ* (ppm) = 64.29, 58.08, 51.44, and 42.51, respectively (+ve peaks) and 8 **C**H_2_ carbon (C_7_–C_14_) at δ (ppm) = 36.17, 28.47, 28.32, 28.02, 26.63, 24.67, 21.59, and 20.31, respectively (-ve peaks) which are clearly observed in DEPT-135 spectra, while two quaternary carbons C_1_ and C_2_ appear at *δ*(ppm) = 79.58 and 69.30, respectively in ^13^C-NMR, while those peaks disappeared from the DEPT-135 spectra (Supplementary Materials Figure S16).

**HMQC** (Supplementary Materials Figure S17) analysis confirmed that C_1_ and C_2_ carbon do not have any proton attached to these carbons but C_3_, C_4_, C_5_, and C_6,_ bear single proton H_2_, H_3_, H_1_, H_5_, respectively, while for eight carbons C_7_–C_14_, each of them is attached to two protons (C_7_-H_9,10_; C_8_-H_18,20_; C_9_-H_11,13_; C_10_-H_7,8_; C_11_-H_4,6_; C_12_-H_12,17_; C_13_-H_15,19_; C_14_-H_14,16_). By this observation, we excluded the two diastereoisomeres formed *via meta*-approach in the [3+2] cycloaddition reaction (32CA) (Scheme 2) [31,32,33,34,35]. 

Next, COSY analysis mapping helped us to find the neighboring protons association. H_1_ and H_2_ coupled with each other while H_2_ also coupled with two other protons (H_9,10_). Proton H_3_ had three strong coupling signals with H_5_ and H_18_,_20_. Similarly, proton H_5_ had five adjacent protons since it had five coupling signals with H_3_, H_9,10_, and H_11,13_ (Figure S18, Supplementary Materials). HMQC Spectra explain the 1–3 and 1–4 interaction of H_1_ with C_2_, C_3_, C_7_, C_11_, and CO which further confirm the position of H_1_ proton (Supplementary Materials Figure S19).

### 2.2. Biological Activity

All synthesized di-spirooxindole analogs **4a–n**, attached with substituted cyclohexanone moiety, were initially examined for toxicity against human fibroblast BJ cell line and appeared to be non-toxic except compound **4g** which was slightly toxic (IC_50_ = 21.7 ± 0.2) at 30 µM concentration. The antiproliferative activity against four cancer cell lines, including prostate PC3, cervical HeLa, and triple-negative breast cancer (MCF-7 and MDA-MB231) was evaluated by MTT assay, while standard drug doxorubicin was used as a reference for comparison (Table 1). 

Among synthesized di-spirooxindole analogs **4a–n**, compound **4b** (IC_50_ = 3.7 ± 1.0 µM) was found to be the most active candidate against prostate cancer PC3 cell line in comparison to standard drug doxorubicin (IC_50_ = 1.9 ± 0.4 µM) and non-substituted spirooxindole analogue **4a** (IC_50_ = 24.1 ± 1.1 µM). Structurally, in comparison to **4a**, compound **4b** consisted of 6-chloro substituted isatin moiety attached to non-substituted phenyl rings containing (2*E*,6*E*)-2,6-dibenzylidenecyclohexanone. The change of 6-chloro phenyl substituents of isatin moiety with 5-flouro, 5-methoxy, and 5 nitro phenyl rings contributed towards a gradual decrease in activity as in compounds **4c** (IC_50_ = 17.9 ± 0.2 µM), **4e** (IC_50_ = 19.6 ± 1.2 µM), and **4d** (IC_50_ = 29.8 ± 0.1 µM), respectively. However, a major increase in activity was observed in compound **4g** (IC_50_ = 14.3 ± 1.0 µM) having a *para*-bromo-substituted benzene rings attached to 5 nitro isatin moiety instead of 5 nitro isatin moiety containing compound **4d** (IC_50_ was 29.8 ± 0.1 µM). All other compounds, i.e., **4f**, and **4h–n** appeared to be inactive against prostate cancer cell line PC3. 

The anticancer potential of the spirooxindole analogs **4a–n**, attached with cyclohexanone moiety, was also evaluated against cervical cancer HeLa cell line in comparison to the standard drug doxorubicin (IC_50_ = 0.9 ± 0.14 µM). The most active spirooxindole analog appeared to be compound **4a** (IC_50_ = 7.1 ± 0.2 µM), having un-substituted isatin and aromatic ring of chlacones moieties. No change in activity was observed for compound **4l** incorporated with 6-choloro isatin and *p*-fluoro-substituted aromatic ring of chlacones moieties (IC_50_ = 7.2 ± 0.5 µM). However, substitution of *p*-fluoro atom of aromatic ring of chlacone moieties with *p*-trifluoromethyl groups contributed towards a decrease in activity as observed in compound **4m** (IC_50_ = 24.6 ± 0.4 µM). The incorporation of methoxy group on isatin ring (**4e**; IC_50_ = 26.5 ± 0.04 µM) also contributed towards a decrease in activity in comparison to **4a** (IC_50_ = 7.1 ± 0.2 µM), having un-substituted isatin and aromatic ring of chlacone moieties. However, a significant improvement of anticancer potential was observed for compound **4j** (IC_50_ = 11.9 ± 0.04 µM) having 6-methoxy isatin and *p*-fluoro-substituted aromatic ring. All other compounds **4b–d, 4f–i, 4k**, and **4n** appeared to be inactive. 

Many of the tested di-spirooxindole analogs **4a–n** attached with cyclohexanone moiety appeared to be inactive against breast cancer MCF-7 cancer cell line, except compounds **4a** (IC_50_ = 25.04 ± 0.57 µM), **4b** (IC_50_ = 27.72 ± 0.59 µM), and **4c** (IC_50_ = 27.82 ± 1.02 µM), which appeared to be weakly active against the MCF-7 cell line in comparison to standard drug, doxorubicin (IC_50_ = 0.79 ± 0.4 µM). 

Finally, all synthesized di-spirooxindole analogues **4a–n** were evaluated against MDA-MB231 triple negative breast cancer cell line. 5-Nitro isatin and *m*-fluoro-phenyl ring containing chlacone **4i** (IC_50_ = 7.63 ± 0.08 µM) appeared to be the most active member of the series. A gradual decrease in activity was observed for compounds **4j** (IC_50_ = 10.49 ± 0.71 µM) and **4h** (IC_50_ = 14.43 ± 0.09 µM) having 5-nitro isatin and 6-chloro isatin moieties, instead of 5-nitro isatin as observed in **4i** (IC_50_ = 7.63 ± 0.08 µM). However, change of position of fluoro substituent from *meta* **4h** (IC_50_ = 14.43 ± 0.09 µM) to *para* **4l** (IC_50_ = 14.45 ± 0.08 µM) on phenyl rings of dibenzylidenecyclohexanone did not exert any effect on activity against MDA-MB231 cell line. Significant reduction in activity was observed in compounds **4b** (IC_50_ = 24.08 ± 0.02 µM) and **4c** (IC_50_ = 20.62 ± 2.16 µM) having non-substituted phenyl rings containing dibenzylidenecyclohexanone moiety attached to 6-chloro and 5-flouro isatin ring, respectively. Compounds **4d–g, 4k, 4m**, and **4n** appeared inactive against the breast cancer cell line (MDA-MB231). All results are summarized in Table 1.

### 2.3. Molecular Docking Study

It has been reported that most of the human cancer cells overexpressed p-53 protein [4,11,36,37]. Therefore, docking studies were performed to rationalize the plausible mechanism of inhibition of p53-MDM2 protein–protein interactions. 

**Figure 3:**
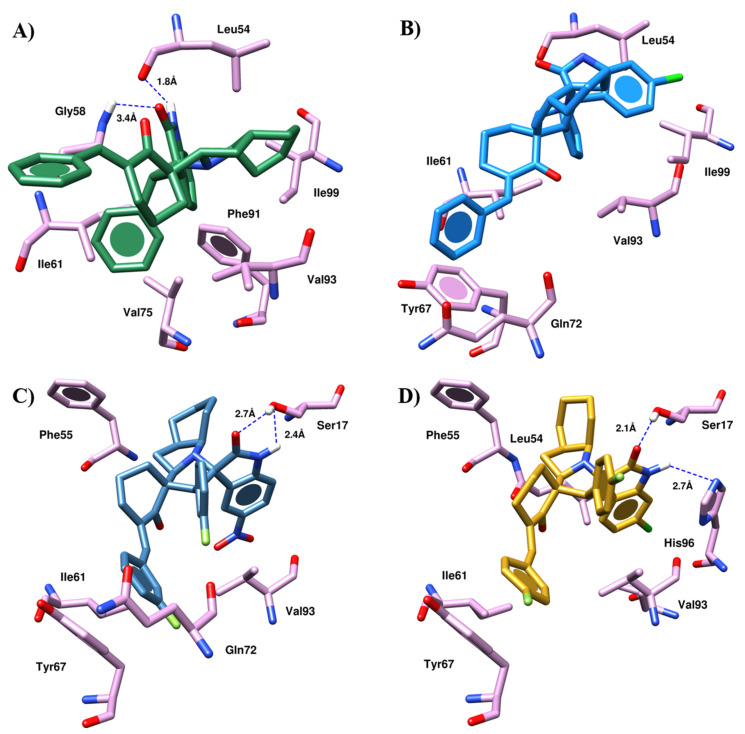
Docked pose of compounds (**A**) **4a**, (**B**) **4b**, (**C**) **4i**, and (**D**) **4l** in the binding site of MDM2.

A tumor suppressor protein p53 plays a pivotal role in preventing tumor progression and development. Cellular stress in response to DNA damage and hypoxia triggers the stimulation of p53. Up-regulated p53 stimulates the transcription of many important genes involved in apoptosis, senescence, DNA repair, and apoptosis. Consequently, suppression of p53 may be a requisite step in tumor formation. Murine double minute 2 (MDM2) is a central negative regulator of p53. Due to the vital role of MDM2 in inhibiting the tumor suppressor function of p53, blockade of protein–protein interaction of MDM2-p53 is an attractive anticancer therapeutic target. Furthermore, it has been extensively reported that spirooxindole analogs potentially inhibit the activity of MDM2 [19,38,39,40]. Thus, in this study, molecular docking studies of the potential anticancer di-spirooxindole analogs were carried out using MDM2 crystal structure to explore the observed anticancer activity. The docking studies suggested that **4a, 4b, 4i**, and **4l** accommodated well in the binding site of MDM2 with a binding affinity of −7.20, −7.37, −7.83, and −7.90 kcal/mol, respectively (Figure 3).

Compound **4a** showed a slightly different binding mode in comparison to **4b**, **4i**, and **4l**. The unsubstituted oxindole ring of **4a** interacts with the side chain of Leu54 and Gly58 while the substituted oxindole ring of **4b**, **4i**, and **4l** is buried deeply in the binding cavity of MDM2 by interacting with Val93, His96, and Ile99. Substitution on the oxindole ring may account for the different binding mode of the di-spirooxindole analogs within the binding cavity of MDM2. Similarly, compound **4a** confers two hydrogen bond contacts with the carboxyl group of Leu54 and Gly58. Six-membered aromatic rings in the **4a** mediate π-π and π-alkyl interactions with the binding site residues of MDM2, including Leu54, Ile61, Val75, Phe91, Val93, and Ile99, which projected **4a** firmly in the binding pocket. The compound **4b** resides comfortably in the binding site extending hydrophobic interactions with Leu54, Ile61, Tyr67, Gln72, Val93, and Ile99, while no hydrogen bond was observed. The docked pose of compound **4i** suggested a number of significant hydrophobic interactions with binding site residues. Fluorinated phenyl rings stacked between Ile61, Tyr67, and Val93 produced hydrophobic effects. Similarly, Phe55 was also observed to mediate hydrophobic interactions. In addition, **4i** confers two hydrogen bonds with the side chain hydroxyl group of Ser17 and a halogen bond with Gln72, whereas fluorinated phenyl rings of compound **4l** establish hydrophobic interactions with Leu57, Phe55, Ile61, Tyr67, Val93, and Ile99. Moreover, two hydrogen bonds were observed with the side chain hydroxyl group and imidazol ring of Ser17 and His96, respectively.

## 3. Materials and Methods

### 3.1. General Procedure for the Synthesis of Di-spirooxindoles *(**4a–n**)* (GP1)

Substituted (2*E*,6*E*)-2,6-dibenzylidenecyclohexanone (**1a–f**) (0.25 mmol), isatin derivatives (**3a–f**) (0.25 mmol) (2*S*)-octahydro-1*H*-indole-2-carboxylic acid (**2**) (63 mg, 0.37 mmol) were dissolved in methanol (20 mL) and refluxed for 1–1.5 h. Finally, the products were isolated by flash column chromatography using 100–200 mesh silica gel and 10–20% ethyl acetate/*n*-hexane as an eluent to afford pure spirooxindoles (**4a-n**). Note for optical rotation measurement: sample prepared in 10 mL, then concentration calculated in g/100 mL. A 100 mm polarimeter tube was used. Instrument used: A.KRÜSS Optronic P8000-PT digital polarimeter (A.KRÜSS Optronic GmbH Alsterdorfer Straße 276–278 22297 Hamburg, Germany).

#### 3.1.1. (1. S,1′S,3′S,4a′S,8a′S,9a′R)-3-((E)-Benzylidene)-1′-phenyl-4a′,5′,6′,7′,8′,8a′,9′,9a′-octahydro-1′H-dispiro[cyclohexane-1,2′-pyrrolo[1,2-a]indole-3′,3″-indoline]-2,2″-dione (**4a**)

2,6-Di((*E*)-benzylidene)cyclohexan-1-one (**1a**) (69 mg, 0.25 mmol), (2*S*)-octahydro-1*H*-indole-2-carboxylic acid (**2**) (64 mg, 0.37 mmol) and isatin (**3a**) (37 mg, 0.25 mmol) were reacted according to GP1 for 1 h and yielded white solid spirooxindole (**4a**) (104 mg, 76%); m.p.: 70–71 °C; ^1^H-NMR (500 MHz, CDCl_3_): δ (ppm) = 7.90 (s, 1H, N**H**), 7.57 (d, *J* = 7.4 Hz, 1H, Ar-**H**), 7.38 (d, *J* = 8.0 Hz, 3H, Ar-**H**), 7.25–7.21 (m, 5H, Ar-**H**), 7.14 (t, *J* = 7.7 Hz, 1H, Ar-**H**), 7.06 (d, *J* = 6.3 Hz, 1H, Ar-**H**), 7.04 (d, *J* = 6.3 Hz, 2H, Ar-**H**), 6.96 (d, *J* = 7.6 Hz, 1H, Ar-**H**), 6.64 (d, *J* = 7.7 Hz, 1H, Ar-**H**), 4.98 (d, *J* = 10.3 Hz, 1H, C**H**), 4.58 (m, 1H, NC**H**), 3.39 (q, *J* = 4.3 Hz, 1H, NC**H**), 2.89–2.81 (m, 1H, C**H**_2_), 2.36–2.30 (m, 1H, C**H**_2_), 2.25–2.18 (m, 1H, C**H**_2_), 2.02–1.97 (m, 3H, C**H**_2_), 1.70–1.66 (m, 1H, C**H**_2_), 1.65–1.60 (m, 3H, C**H**_2_), 1.54–1.52 (m, 2H, C**H**_2_), 1.02–0.96 (m, 3H, C**H**_2_); ^13^C-NMR (126 MHz, CDCl_3_): δ (ppm) = 202.1 (**C**O), 180.2 (**C**O), 141.3, 138.6, 137.8, 135.8, 134.8, 130.1, 129.7, 129.1, 128.5, 128.3, 128.2, 128.1, 127.1, 126.8, 121.4, 109.7, 79.7, 69.5, 64.3, 57.9, 52.3, 42.6, 36.3, 28.5, 28.4, 27.9, 26.6, 24.8, 21.7, 20.4; IR (KBr, cm^−1^) ν_max_ = 3057, 2928, 2854, 1711, 1683, 1617, 1600, 1521, 1492, 1471, 1447, 1365, 1321, 1289, 1199, 1154, 1030, 961, 753, 697; (Anal. Calcd. for C_36_H_36_N_2_O_2_: C, 81.79; H, 6.86; N, 5.30; Found: C, 81.88; H, 6.94; N, 5.21); LC/MS (ESI, *m*/*z*): found 529.3 [M+H]^+^; exact mass 528.3 for C_36_H_36_N_2_O_2_. ${\left[\alpha \right]}_{D}^{25}$ = −20.72° (c 0.10, MeOH).

#### 3.1.2. (1. S,1′S,3′S,4a′S,8a′S,9a′R)-3-((E)-Benzylidene)-6″-chloro-1′-phenyl-4a′,5′,6′,7′,8′,8a′,9′,9a′-octahydro-1′H-dispiro[cyclohexane-1,2′-pyrrolo[1,2-a]indole-3′,3″-indoline]-2,2″-dione (**4b**)

2,6-Di((*E*)-benzylidene)cyclohexan-1-one (**1a**) (69 mg, 0.25 mmol), (2*S*)-octahydro-1*H*-indole-2-carboxylic acid (**2**) (64 mg, 0.37 mmol) and 6-chloroisatin (**3b**) (45 mg, 0.25 mmol) were reacted according to GP1 for 1 h and yielded white solid spirooxindole (**4b**) (98 mg, 70%); m.p.: 77–78 °C; ^1^H-NMR (500 MHz, CDCl_3_): δ (ppm) = 8.14 (s, 1H, N**H**), 7.46 (d, *J* = 8.2 Hz, 1H, Ar-**H**), 7.34 (d, *J* = 8.3 Hz, 3H, Ar-**H**), 7.25–7.21 (m, 6H, Ar-**H**), 7.06 (d, *J* = 8.0 Hz, 2H, Ar-**H**), 6.93 (d, *J* = 8.0 Hz, 1H, Ar-**H**), 6.70 (s, 1H, Ar-**H**), 4.94 (d, *J* = 10.3 Hz, 1H, C**H**), 4.58–4.53 (m, 1H, NC**H**), 3.37 (q, *J* = 4.0 Hz, 1H, NC**H**), 2.79–2.73 (m, 1H, C**H**_2_), 2.34–2.28 (m, 1H, C**H**_2_), 2.25–2.18 (m, 1H, C**H**_2_), 2.01–1.94 (m, 2H, C**H**_2_), 1.70–1.64 (d, *J* = 6.1 Hz, 2H, C**H**_2_), 1.64–1.58 (m, 3H, C**H**_2_), 1.53–1.48 (m, 2H, C**H**_2_), 1.28–1.22 (m, 2H, C**H**_2_), 1.07–0.97 (m, 3H, C**H**_2_); ^13^C-NMR (126 MHz, CDCl_3_): δ (ppm) = 202.0 (**C**O), 180.0 (**C**O), 142.6, 138.4, 137.5, 135.7, 135.4, 134.8, 130.8, 130.2, 129.7, 128.5, 128.3, 128.1, 127.9, 126.9, 121.4, 110.3, 79.3, 69.8, 64.2, 57.9, 52.6, 42.6, 36.2, 28.6, 28.4, 28.1, 26.8, 24.8, 21.6, 20.4; IR (KBr, cm^−1^) ν_max_ = 3058, 2928, 2853, 1716, 1685, 1619, 1608, 1525, 1491, 1470, 1448, 1363, 1317, 1275, 1199, 1153, 1032, 965, 758, 696; (Anal. Calcd. for C_36_H_35_ClN_2_O_2_: C, 76.78; H, 6.26; N, 4.97; Found: C, 76.71; H, 6.35; N, 5.07); LC/MS (ESI, *m*/*z*): found 563.1 [M(_35_Cl)+H]^+^; 565.1 [M(_37_Cl)+H]^+^; exact mass 562.1 for C_36_H_35_ClN_2_O_2_. ${\left[\alpha \right]}_{D}^{25}$ = −17.54° (c 0.19, MeOH).

#### 3.1.3. (1. S,1′S,3′S,4a′S,8a′S,9a′R)-3-((E)-Benzylidene)-5″-fluoro-1′-phenyl-4a′,5′,6′,7′,8′,8a′,9′,9a′-octahydro-1′H-dispiro[cyclohexane-1,2′-pyrrolo[1,2-a]indole-3′,3″-indoline]-2,2″-dione (**4c**)

2,6-Di((*E*)-benzylidene)cyclohexan-1-one (**1a**) (69 mg, 0.25 mmol), (2*S*)-octahydro-1*H*-indole-2-carboxylic acid (**2**) (64 mg, 0.37 mmol) and 5-fluoroisatin (**3c**) (41 mg, 0.25 mmol) were reacted according to GP1 for 1h and yielded white solid spirooxindole (**4c**) (104 mg, 76%); m.p.: 70–71 °C; ^1^H-NMR (500 MHz, CDCl_3_): δ (ppm) = 8.56 (s, 1H, N**H**), 7.37–7.34 (m, 3H, Ar-**H**), 7.24–7.22 (m, 6H, Ar-**H**), 7.09–7.05 (m, 3H, Ar-**H**), 6.86 (td, *J* = 8.8, 2.7 Hz, 1H, Ar-**H**), 6.65 (dd, *J* = 8.5, 4.3 Hz, 1H, Ar-**H**), 4.98 (d, *J* = 10.4 Hz, 1H, C**H**), 4.60–4.56 (m, 1H, NC**H**), 3.42–3.37 (m, 1H, NC**H**), 2.79–2.73 (m, 1H, C**H**_2_), 2.39–2.20 (m, 4H, C**H**_2_), 2.07–1.94 (m, 3H, C**H**_2_), 1.73–1.57 (m, 6H, C**H**_2_), 1.08–0.99 (m, 3H, C**H**_2_); ^13^C-NMR (126 MHz, CDCl_3_): δ (ppm) = 201.8 (**C**O), 180.7 (**C**O), 159.4, 157.5, 138.4, 137.4, 137.4, 136.5, 135.7, 135.2, 130.7, 130.5, 130.2, 130.1, 129.7, 128.4, 128.3, 128.14, 126.9, 115.6, 115.4, 114.7, 114.5, 110.3, 110.3, 79.9, 69.8, 64.2, 57.9, 52.7, 42.5, 36.12, 28.5, 28.3, 28.1, 26.7, 24.7, 21.6, 20.4; IR (KBr, cm^−1^) ν_max_ = 3054, 2929, 2852, 1715, 1680, 1614, 1605, 1527, 1495, 1476, 1444, 1368, 1321, 1284, 1196, 1151, 1007, 962, 755, 693; (Anal. Calcd. for C_36_H_35_FN_2_O_2_: C, 79.09; H, 6.45; N, 5.12; Found: C, 79.21; H, 6.58; N, 5.07); LC/MS (ESI, *m*/*z*): found 547.3 [M+H]^+^; exact mass 546.3 for C_36_H_35_FN_2_O_2_. ${\left[\alpha \right]}_{D}^{25}$ = −39.87° (c 0.10, MeOH).

#### 3.1.4. (1. S,1′S,3′S,4a′S,8a′S,9a′R)-3-((E)-Benzylidene)-5″-nitro-1′-phenyl-4a′,5′,6′,7′,8′,8a′,9′,9a′-octahydro-1′H-dispiro[cyclohexane-1,2′-pyrrolo[1,2-a]indole-3′,3″-indoline]-2,2″-dione (**4d**)

2,6-Di((*E*)-benzylidene)cyclohexan-1-one (**1a**) (69 mg, 0.25 mmol), (2*S*)-octahydro-1*H*-indole-2-carboxylic acid (**2**) (64 mg, 0.37 mmol) and 5-nitroisatin (**3d**) (48 mg, 0.25 mmol) were reacted according to GP1 for 1 h and yielded white solid spirooxindole (**4d**) (98 mg, 68%); m.p.: 111–122 °C; ^1^H-NMR (500 MHz, CDCl_3_): δ (ppm) = 8.47 (s, 1H, N**H**), 7.50–7.44 (m, 3H, Ar-**H**), 7.35–7.32 (m, 2H, Ar-**H**), 7.31–7.26 (m, 1H, Ar-**H**), 7.24–7.21 (m, 2H, Ar-**H**), 7.19–7.17 (m, 1H, Ar-**H**), 7.13 (d, *J* = 8.2 Hz, 1H, Ar-**H**), 7.12–7.09 (m, 2H, Ar-**H**), 7.02–6.99 (m, 1H, Ar-**H**), 6.74 (q, *J* = 1.7 Hz, 1H, Ar-**H**), 4.91 (d, *J* = 10.2 Hz, 1H, C**H**), 4.53–4.46 (m, 1H, NC**H**), 3.34 (q, *J* = 3.9 Hz, 1H, NC**H**), 2.92 -2.87 (m, 1H, C**H**_2_), 2.82–2.73 (m, 1H, C**H**_2_), 2.39–2.29 (m, 2H, C**H**_2_), 2.19–2.14 (m, 1H, C**H**_2_), 2.08–2.00 (m, 3H, C**H**_2_), 1.96–1.92 (m, 1H, C**H**_2_), 1.71–1.59 (m, 5H, C**H**_2_), 1.08–1.01 (m, 3H, C**H**_2_); ^13^C-NMR (126 MHz, CDCl_3_): δ (ppm) = 201.8 (**C**O), 180.1 (**C**O), 142.5, 139.9, 139.5, 137.6, 135.1, 133.7, 132.7, 132.5, 131.4, 130.2, 129.7, 128.6, 127.9, 122.5, 121.6, 110.5, 79.4, 69.6, 64.2, 58.0, 52.1, 42.5, 36.1, 28.6, 28.3, 28.0, 26.8, 24.6, 21.5, 20.9; IR (KBr, cm^−1^) ν_max_ = 3262, 2926, 2853, 1715, 1681, 1612, 1592, 1558, 1484, 1473, 1447, 1365, 1317, 1260, 1200, 1155, 1102, 1073, 995, 961, 914, 846, 784, 683; (Anal. Calcd. for C_36_H_35_N_3_O_4_: C, 75.37; H, 6.15; N, 7.32; Found: C, 75.24; H, 6.01; N, 7.23); LC/MS (ESI, *m*/*z*): found 574.3 [M+H]^+^; exact mass 573.26 for C_36_H_35_N_3_O_4_. ${\left[\alpha \right]}_{D}^{25}$ = −15.78° (c 0.13, MeOH).

#### 3.1.5. (1. S,1′S,3′S,4a′S,8a′S,9a′R)-3-((E)-Benzylidene)-5″-methoxy-1′-phenyl-4a′,5′,6′,7′,8′,8a′,9′,9a′-octahydro-1′H-dispiro[cyclohexane-1,2′-pyrrolo[1,2-a]indole-3′,3″-indoline]-2,2″-dione (**4e**)

2,6-Di((*E*)-benzylidene)cyclohexan-1-one (**1a**) (69 mg, 0.25 mmol), (2*S*)-octahydro-1*H*-indole-2-carboxylic acid (**2**) (64 mg, 0.37 mmol) and 5-methoxyisatin (**3e**) (44 mg, 0.25 mmol) were reacted according to GP1 for 1 h and yielded white solid spirooxindole (**4e**) (91 mg, 65%); m.p.: 111–122 °C; ^1^H-NMR (500 MHz, CDCl_3_): δ (ppm) = 7.36 (t, *J* = 6.9 Hz, 4H, Ar-**H**), 7.23 (d, *J* = 5.7 Hz, 2H, Ar-**H**), 7.20 (d, *J* = 2.7 Hz, 1H, Ar-**H**), 7.13 (s, 1H, Ar-**H**), 7.10 (d, *J* = 6.7 Hz, 2H, Ar-**H**), 7.07 (d, *J* = 6.4 Hz, 2H, Ar-**H**), 6.70 (dd, *J* = 8.4, 2.6 Hz, 1H, Ar-**H**), 6.52 (d, *J* = 8.4 Hz, 1H, Ar-**H**), 4.96 (d, *J* = 10.4 Hz, 1H, C**H**), 4.59–4.55 (m, 1H, NC**H**), 3.79 (s, 3H, C**H**_3_), 3.41 (d, *J* = 4.5 Hz, 1H, NC**H**), 2.86–2.80 (m, 1H, C**H**_2_), 2.38–2.29 (m, 2H, C**H**_2_), 2.27–2.15 (m, 2H, C**H**_2_), 2.09–1.96 (m, 5H, C**H**_2_), 1.75–1.64 (m, 3H, C**H**_2_), 1.08–0.98 (m, 4H, C**H**_2_); ^13^C-NMR (126 MHz, CDCl_3_): δ (ppm) = 201.9 (**C**O), 181.1 (**C**O), 155.0, 138.7, 137.7, 137.1, 134.8, 131.6, 130.8, 130.5, 130.1, 129.8, 128.5, 128.3, 128.1, 126.8, 114.1, 109.7, 79.9, 69.6, 64.3, 57.9, 56.1 (O**C**H_3_), 52.54, 42.55, 36.21, 28.53, 28.39, 27.94, 26.50, 24.79, 21.68, 20.44; IR (KBr, cm^−1^) ν_max_ = 3267, 2928, 2854, 1718, 1685, 1610, 1597, 1554, 1483, 1471, 1449, 1363, 1317, 1265, 1202, 1154, 1105, 1077, 993, 965, 912, 844, 787, 686; (Anal. Calcd. for C_37_H_38_N_2_O_3_: C, 79.54; H, 6.86; N, 5.01; Found: C, 79.68; H, 6.74; N, 5.11); LC/MS (ESI, *m*/*z*): found 559.3 [M+H]^+^; exact mass 558.28 for C_37_H_38_N_2_O_3_. ${\left[\alpha \right]}_{D}^{25}$ = −14.21° (c 0.06, MeOH).

#### 3.1.6. (1. S,1′S,3′S,4a′S,8a′S,9a′R)-3-((E)-4-Bromobenzylidene)-1′-(4-bromophenyl)-6″-chloro-4a′,5′,6′,7′,8′,8a′,9′,9a′-octahydro-1′H-dispiro[cyclohexane-1,2′-pyrrolo[1,2-a]indole-3′,3″-indoline]-2,2″-dione (**4f**)

2,6-Bis((*E*)-4-bromobenzylidene)cyclohexan-1-one (**1b**) (108 mg, 0.25 mmol), (2*S*)-octahydro-1*H*-indole-2-carboxylic acid (**2**) (64 mg, 0.37 mmol) and 6-chloroisatin (**3b**) (45 mg, 0.25 mmol) were reacted according to GP1 for 1h and yielded white solid spirooxindole (**4f**) (124 mg, 69%); m.p: 97–98 °C; ^1^H-NMR (400 MHz, CDCl_3_): δ (ppm) = 8.55 (s, 1H, N**H**), 7.48 (d, *J* = 8.0 Hz, 1H, Ar-**H**), 7.40–7.34 (m, 4H, Ar-**H**), 7.24–7.22 (m, 2H, Ar-**H**), 7.20–7.17 (m, 1H, Ar-**H**), 6.97 (dd, *J* = 8.1, 1.9 Hz, 1H, Ar-**H**), 6.93 (d, *J* = 8.5 Hz, Ar-**H**), 6.73 (d, *J* = 1.8 Hz, 1H, Ar-**H**), 4.89 (d, *J* = 10.4 Hz, 1H, C**H**), 4.49 (td, *J* = 10.0, 5.8 Hz, 1H, NC**H**), 3.35 (q, *J* = 3.9 Hz, 1H, NC**H**), 2.80–2.70 (m, 1H, C**H**_2_), 2.36–2.26 (m, 1H, C**H**_2_), 2.19–2.10 (m, 1H, C**H**_2_), 2.04–1.90 (m, 3H, C**H**_2_), 1.67–1.58 (m, 3H, C**H**_2_), 1.52–1.43 (m, 1H, C**H**_2_), 1.42–1.31 (m, 3H, C**H**_2_), 1.19–1.09 (m, 2H, C**H**_2_), 1.05–0.95 (m, 3H, C**H**_2_); ^13^C-NMR (101 MHz, CDCl_3_): δ (ppm) = 202.03 (Hex-**C**O), 180.02 (**C**O), 142.54, 138.77, 136.57, 134.97, 134.38, 134.03, 131.67, 131.60, 131.39, 131.26, 128.30, 127.90, 122.87, 121.56, 120.96, 110.49, 79.58, 69.30, 64.29, 58.08, 51.44, 42.51, 36.17, 28.47, 28.32, 28.02, 26.63, 24.67, 21.59, 20.31; IR (KBr, cm^−1^) ν_max_ = 3242, 2927, 2853, 1716, 1684, 1612, 1487, 1446, 1316, 1260, 1153, 1073, 1010, 851, 720, 691; (Anal. Calcd. for C_36_H_33_Br_2_ClN_2_O_2_: C, 59.98; H, 4.61; N, 3.89; Found: C, 60.12; H, 4.73; N, 3.93); LC/MS (ESI, *m*/*z*): found 719.1 [M(_35_Cl)+H]^+^; 721.1 [M(_37_Cl)+H]^+^, 723.1 [M(_37_Cl+_81_Br)+H]^+^, exact mass 718.06 for C_36_H_33_Br_2_ClN_2_O_2_. ${\left[\alpha \right]}_{D}^{25}$ = −24.26° (c 0.11, MeOH).

#### 3.1.7. (1. S,1′S,3′S,4a′S,8a′S,9a′R)-3-((E)-4-bromobenzylidene)-1′-(4-bromophenyl)-5″-nitro-4a′,5′,6′,7′,8′,8a′,9′,9a′-octahydro-1′H-dispiro[cyclohexane-1,2′-pyrrolo[1,2-a]indole-3′,3″-indoline]-2,2″-dione (**4g**)

2,6-*Bis*((*E*)-4-bromobenzylidene)cyclohexan-1-one (**1b**) (108 mg, 0.25 mmol), (2*S*)-octahydro-1*H*-indole-2-carboxylic acid (**2**) (64 mg, 0.37 mmol) and 5-nitroisatin (**3d**) (48 mg, 0.25 mmol) were reacted according to GP1 for 1 h and yielded white solid spirooxindole (**4g**) (123 mg, 67%); m.p.: 125–126 °C; ^1^H-NMR (500 MHz, CDCl_3_): δ (ppm) = 8.47 (d, *J* = 2.4 Hz, 1H, Ar-**H**), 8.14 (dd, *J* = 8.6, 2.4 Hz, 1H, Ar-**H**), 8.01 (s, 1H, N**H**), 7.47 (d, *J* = 7.2 Hz, 1H, Ar-**H**), 7.41 (t, *J* = 7.6 Hz, 1H, Ar-**H**), 7.37 (d, *J* = 7.4 Hz, 1H, Ar-**H**), 7.34–7.31 (m, 3H, Ar-**H**), 7.07 (dd, *J* = 7.7, 1.9 Hz, 2H, Ar-**H**), 6.80 (dt, *J* = 8.6, 1.4 Hz, 1H, Ar-**H**), 4.90 (d, *J* = 10.5 Hz, 1H, C**H**), 4.67–4.61 (m, 1H, NC**H**), 3.38 (q, *J* = 3.8 Hz, 1H, NC**H**), 2.97–2.92 (m, 1H, C**H**_2_), 2.85–2.78 (m, 1H, C**H**_2_), 2.43–2.28 (m, 3H, C**H**_2_), 2.18–2.10 (m, 2H, C**H**_2_), 1.99–1.92 (m, 2H, C**H**_2_), 1.73–1.61 (m, 5H, C**H**_2_), 1.11–0.99 (m, 3H, C**H**_2_); ^13^C-NMR (126 MHz, CDCl_3_): δ (ppm) = 201.8 (**C**O), 179.5 (**C**O), 147. 1, 143.4, 138.1, 137.1, 136.1, 135.4, 130.8, 130.5, 130.2, 129.6, 128.8, 128.5, 128.3, 126.2, 122.6, 109.3, 79.1, 70.6, 64.2, 57.8, 53.8, 42.6, 35.9, 28.7, 28.6, 28.2, 27.2, 24.6, 21.4, 20.2; IR (KBr, cm^−1^) ν_max_ = 2927, 2854, 1740, 1668, 1619, 1606, 1523, 1507, 1446, 1336, 1272, 1164, 1082, 1028, 965, 909, 842, 773, 747, 732, 696; (Anal. Calcd. for C_36_H_33_Br_2_N_3_O_4_: C, 59.11; H, 4.55; N, 5.74; Found: C, 59.24; H, 4.43; N, 5.81); LC/MS (ESI, *m*/*z*): found 730.1 [M(_79_Br)+H]^+^; 732.1 [M(_81_Br)+H]^+^, exact mass 729.08 for C_36_H_33_Br_2_N_3_O_4_. ${\left[\alpha \right]}_{D}^{25}$ = −12.64° (c 0.12, MeOH).

#### 3.1.8. (1. S,1′S,3′S,4a′S,8a′S,9a′R)-6″-Chloro-3-((E)-3-fluorobenzylidene)-1′-(3-fluorophenyl)-4a′,5′,6′,7′,8′,8a′,9′,9a′-octahydro-1′H-dispiro[cyclohexane-1,2′-pyrrolo[1,2-a]indole-3′,3″-indoline]-2,2″-dione (**4h**)

2,6-*Bis*((*E*)-3-fluorobenzylidene)cyclohexan-1-one (**1d**) (78 mg, 0.25 mmol), (2*S*)-octahydro-1*H*-indole-2-carboxylic acid **2** (64 mg, 0.37 mmol) and 6-chloroisatin (**3b**) (45 mg, 0.25 mmol) were reacted according to GP1 for 1 h and yielded white solid spirooxindole (**4h**) (109 mg, 73%); m.p.: 83–84 °C; ^1^H-NMR (500 MHz, CDCl_3_): δ (ppm) = 8.82 (s, 1H, N**H**), 7.48 (d, *J* = 8.1 Hz, 1H, Ar-**H**), 7.25–7.18 (m, 3H, Ar-**H**), 7.13 (d, *J* = 8.1 Hz, 1H, Ar-**H**), 7.12–7.08 (m, 1H, Ar-**H**), 6.97 (dd, *J* = 8.1, 1.9 Hz, 1H, Ar-**H**), 6.94–6.88 (m, 3H, Ar-**H**), 6.81–6.77 (m, 1H, Ar-**H**), 6.74 (d, *J* = 1.9 Hz, 1H, Ar-**H**), 4.96 (d, *J* = 10.3 Hz, 1H, C**H**), 4.51 (td, *J* = 10.0, 5.8 Hz, 1H, NC**H**), 3.36 (q, *J* = 3.7 Hz, 1H, NC**H**), 2.79–2.73 (m, 1H, C**H**_2_), 2.37–2.30 (m, 1H, C**H**_2_), 2.22–2.15 (m, 1H, C**H**_2_), 2.09–2.05 (m, 2H, C**H**_2_), 2.01–1.94 (m, 1H, C**H**_2_), 1.73 -1.67 (m, 1H, C**H**_2_), 1.66–1.60 (m, 2H, C**H**_2_), 1.59–1.55(m, 1H, C**H**_2_), 1.45–1.32 (m, 4H, C**H**_2_), 1.07–0.97 (m, 3H, C**H**_2_); ^13^C-NMR (126 MHz, CDCl_3_): δ (ppm) = 202.0 (**C**O), 180.4 (**C**O), 163.7, 163.5, 161.7, 161.6, 142.6, 140.3, 140.3, 139.4, 137.8, 137.8, 135.1, 134.0, 129.9, 129.8, 129.6, 129.5, 128.2, 127.9, 126.1, 126.1, 125.6, 121.6, 116.6, 116.5, 116.4, 116.3, 115.5, 115.3, 113.9, 113.7, 110.6, 79.7, 69.5, 64.4, 58.1, 51.6, 42.5, 36.2, 28.5, 28.3, 28.1, 26.8, 24.7, 21.5, 20.3; IR (KBr, cm^−1^) ν_max_ = 2929, 2854, 1714, 1681, 1612, 1584, 1366, 1317, 1276, 1236, 1201, 1151, 1074, 976, 906, 787, 732, 686; (Anal. Calcd. for C_36_H_33_ClF_2_N_2_O_2_: C, 72.17; H, 5.55; N, 4.68; Found: C, 71.96; H, 5.63; N, 4.59); LC/MS (ESI, *m*/*z*): found 599.2 [M(_35_Cl)+H]^+^; 601.2 [M(_37_Cl)+H]^+^, exact mass 598.22 for C_36_H_33_ClF_2_N_2_O_2_. ${\left[\alpha \right]}_{D}^{25}$ = −44.98° (c 0.11, MeOH).

#### 3.1.9. (1. S,1′S,3′S,4a′S,8a′S,9a′R)-3-((E)-3-Fluorobenzylidene)-1′-(3-fluorophenyl)-5″-nitro-4a′,5′,6′,7′,8′,8a′,9′,9a′-octahydro-1′H-dispiro[cyclohexane-1,2′-pyrrolo[1,2-a]indole-3′,3″-indoline]-2,2″-dione (**4i**)

2,6-*Bis*((*E*)-3-fluorobenzylidene)cyclohexan-1-one (**1d**) (78 mg, 0.25 mmol), (2*S*)-octahydro-1*H*-indole-2-carboxylic acid (**2**) (64 mg, 0.37 mmol) and 5-nitroisatin (**3d**) (48 mg, 0.25 mmol) were reacted according to GP1 for 1h and yielded white solid spirooxindole (**4i**) (100 mg, 66%); m.p.: 107–108 °C; ^1^H-NMR (500 MHz, CDCl_3_): δ (ppm) = 8.91 (s, 1H, N**H**), 8.46 (d, *J* = 2.2 Hz, 1H, Ar-**H**), 8.15 (dd, *J* = 8.6, 2.4 Hz, 1H, Ar-**H**), 7.23–7.19 (m, 2H, Ar-**H**), 7.12–7.00 (m, 3H, Ar-**H**), 6.99–6.90 (m, 2H, Ar-**H**), 6.90–6.83 (m, 2H, Ar-**H**), 6.81–6.77 (m, 1H, Ar-**H**), 4.90 (d, *J* = 10.53, 1H, C**H**), 4.60–4.55 (m, 1H, NC**H**), 3.36 (q, *J* = 4.1 Hz, 1H, NC**H**), 2.83–2.77 (m, 1H, C**H**_2_), 2.39–2.33 (m, C**H**_2_), 2.31–2.26 (m, 1H, C**H**_2_), 2.06–2.00 (m, 1H, C**H**_2_), 1.96–1.91 (m, 1H, C**H**_2_), 1.74–1.59 (m, 6H, C**H**_2_), 1.57–1.50 (m, 3H, C**H**_2_), 1.10–1.00 (m, 3H, C**H**_2_); ^13^C-NMR (126 MHz, CDCl_3_): δ (ppm) = 201.7 (**C**O), 180.3 (**C**O), 163.7, 163.6, 161.8, 161.6, 147.2, 142.9, 139.4, 139.1, 137.4, 134.7, 131.0, 130.0, 130.0, 129.8, 126.3, 125.9, 125.4, 122.6, 116.7, 116.6, 116.5, 116.4, 115.9, 115.7, 114.3, 114.2, 114.1, 109.7, 79.4, 70.2, 64.3, 58.1, 52.9, 42.5, 35.9, 28.6, 28.3, 28.2, 27.2, 24.5, 21.2, 20.2; IR (KBr, cm^−1^) ν_max_ = 2924, 2854, 1734, 1719, 1610, 1584, 1525, 1507, 1483, 1448, 1337, 1278, 1244, 1224, 1168, 1151, 968, 906, 840, 788, 684; (Anal. Calcd. for C_36_H_33_F_2_N_3_O_4_: C, 70.92; H, 5.46; N, 6.89; Found: C, 71.08; H, 5.37; N, 6.74); LC/MS (ESI, *m*/*z*): found 510 [M+H]^+^; exact mass 609.24 for C_36_H_33_F_2_N_3_O_4_. ${\left[\alpha \right]}_{D}^{25}$ = +20.79° (c 0.13, MeOH).

#### 3.1.10. (1. S,1′S,3′S,4a′S,8a′S,9a′R)-3-((E)-3-Fluorobenzylidene)-1′-(3-fluorophenyl)-5″-methoxy-4a′,5′,6′,7′,8′,8a′,9′,9a′-octahydro-1′H-dispiro[cyclohexane-1,2′-pyrrolo[1,2-a]indole-3′,3″-indoline]-2,2″-dione (**4j**)

2,6-*Bis*((*E*)-3-fluorobenzylidene)cyclohexan-1-one (**1d**) (78 mg, 0.25 mmol), (2*S*)-octahydro-1*H*-indole-2-carboxylic acid (**2**) (64 mg, 0.37 mmol) and 5-methoxyisatin (**3e**) (44 mg, 0.25 mmol) were reacted according to GP1 for 1 h and yielded white solid spirooxindole (**4j**) (106 mg, 71%); m.p.: 92–93 °C; ^1^H-NMR (500 MHz, CDCl_3_): δ (ppm) = 8.12 (s, 1H, N**H**), 7.24–7.18 (m, 4H, Ar-**H**), 7.18–7.12 (m, 2H, Ar-**H**), 7.01 (d, *J* = 10.7 Hz, 2H, Ar-**H**), 6.88 (d, *J* = 7.0 Hz, 2H, Ar-**H**), 6.71 (dd, *J* = 8.4, 2.6 Hz, 1H, Ar-**H**), 6.58 (d, *J* = 8.4 Hz, 1H, Ar-**H**), 4.97 (d, *J* = 10.3 Hz, 1H, C**H**), 4.50 (dt, *J* = 10.0, 5.0 Hz, 1H, NC**H**), 3.79 (s, 3H, C**H**_3_), 3.38 (q, *J* = 3.8 Hz, 1H, NC**H**), 2.82 (ddd, *J* = 14.3, 9.7, 4.3 Hz, 1H, C**H**_2_), 2.38–2.30 (m, 2H, C**H**_2_), 2.04 (ddt, *J* = 12.0, 6.7, 3.9 Hz, 3H, C**H**_2_), 1.99–1.93 (m, 1H, C**H**_2_), 1.71–1.56 (m, 6H, C**H**_2_), 1.17–1.13 (m, 4H, C**H**_2_); ^13^C-NMR (126 MHz, CDCl_3_): δ (ppm) = 201.9 (**C**O), 180.19 (**C**O), 163.7, 163.5, 161.7, 161.5, 155.1, 139.9, 139.6, 138.1, 138.0, 135.0, 134.8, 133.3, 131.8, 129.8, 129.7, 129.5, 129.4, 128.6, 126.0, 125.6, 116.6, 116.5, 116.5, 116.4, 115.3, 115.1, 114.3, 114.2, 113.9, 113.8, 113.7, 113.6, 110.1, 80.3, 70.0, 64.4, 58.1, 56.11 (O**C**H_3_), 51.60, 42.49, 36.21, 28.46, 28.33, 27.94, 26.45, 24.70, 21.56, 20.40; IR (KBr, cm^−1^) ν_max_ = 2926, 2855, 1736, 1717, 1612, 1581, 1524, 1509, 1485, 1444, 1332, 1274, 1242, 1224, 1165, 1154, 962, 904, 847, 784; (Anal. Calcd. for C_37_H_36_F_2_N_2_O_3_: C, 74.73; H, 6.10; N, 4.71; Found: C, 74.64; H, 6.25; N, 4.82); LC/MS (ESI, *m*/*z*): found 595.2 [M+H]^+^; exact mass 594.27 for C_37_H_36_F_2_N_2_O_3_. ${\left[\alpha \right]}_{D}^{25}$ = −7.51° (c 0.10, MeOH).

#### 3.1.11. (1. S,1′S,3′S,4a′S,8a′S,9a′R)-3-((E)-3-Bromobenzylidene)-1′-(3-bromophenyl)-6″-chloro-4a′,5′,6′,7′,8′,8a′,9′,9a′-octahydro-1′H-dispiro[cyclohexane-1,2′-pyrrolo[1,2-a]indole-3′,3″-indoline]-2,2″-dione (**4k**)

2,6-*Bis*((*E*)-3-bromobenzylidene)cyclohexan-1-one (**1c**) (108 mg, 0.25 mmol), (2*S*)-octahydro-1*H*-indole-2-carboxylic acid (**2**) (64 mg, 0.37 mmol) and 6-chloroisatin (**3b**) (45 mg, 0.25 mmol) were reacted according to GP1 for 1 h and yielded white solid spirooxindole (**4k**) (133 mg, 74%); m.p.: 115–116 °C; ^1^H-NMR (500 MHz, CDCl_3_): δ (ppm) = 8.42 (s, 1H, N**H**), 7.48 (d, *J* = 4.9 Hz, 1H, Ar-**H**), 7.46 (d, *J* = 3.9 Hz, 1H, Ar-**H**), 7.34 (d, *J* = 7.9 Hz, 2H, Ar-**H**), 7.23 (s, 1H, Ar-**H**), 7.18 (s, 1H), 7.12 (q, *J* = 7.9 Hz, 3H, Ar-**H**), 7.00 (d, *J* = 8.6 Hz, 1H, Ar-**H**), 6.97 (d, *J* = 8.2 Hz, 1H, Ar-**H**), 6.75 (d, *J* = 2.3 Hz, 1H, Ar-**H**), 4.91 (d, *J* = 10.3 Hz, 1H, C**H**), 4.54–4.47 (m, 1H, NC**H**), 3.35 (q, *J* = 4.0 Hz, 1H, NC**H**), 2.80–2.73 (m, 1H, C**H**_2_), 2.41–2.29 (m, 2H, C**H**_2_), 2.19–2.13 (m, 1H, C**H**_2_), 2.07–2.01 (m, 2H, C**H**_2_), 1.70–1.60 (m, 4H, C**H**_2_), 1.58–1.53 (m, 2H, C**H**_2_), 1.53–1.47 (m, 2H, C**H**_2_), 1.09–0.98 (m, 3H, C**H**_2_); ^13^C-NMR (126 MHz, CDCl_3_): δ (ppm) = 201.8 (**C**O), 180.1 (**C**O), 142.5, 139.9, 139.5, 137.6, 135.1, 133.8, 132.8, 132.7, 132.5, 131.4, 130.2, 129.9, 129.8, 128.6, 128.5, 128.1, 127.9, 122.5, 121.7, 110.5, 79.4, 69.6, 64.2, 58.0, 52.1, 42.5, 36.1, 28.5, 28.3, 28.0, 26.8, 24.6, 21.5, 20.4; IR (KBr, cm^−1^) ν_max_ = 2926, 2853, 1715, 1682, 1612, 1591, 1558, 1473, 1446, 1316, 1272, 1200, 1155, 1073, 995, 961, 930, 856, 784, 730, 682; (Anal. Calcd. for C_36_H_33_Br_2_ClN_2_O_2_: C, 59.98; H, 4.61; N, 3.89; Found: C, 60.86; H, 4.53; N, 3.87); LC/MS (ESI, *m*/*z*): found 719.1 [M(_35_Cl)+H]^+^; 721.1 [M(_37_Cl)+H]^+^, 723.1 [M(_37_Cl+_81_Br)+H]^+^, exact mass 718.06 for C_36_H_33_Br_2_ClN_2_O_2_. ${\left[\alpha \right]}_{D}^{25}$ = −15.68° (c 0.10, MeOH).

#### 3.1.12. (1. S,1′S,3′S,4a′S,8a′S,9a′R)-6″-Chloro-3-((E)-4-fluorobenzylidene)-1′-(4-fluorophenyl)-4a′,5′,6′,7′,8′,8a′,9′,9a′-octahydro-1′H-dispiro[cyclohexane-1,2′-pyrrolo[1,2-a]indole-3′,3″-indoline]-2,2″-dione (**4l**)

2,6-*Bis*((*E*)-4-fluorobenzylidene)cyclohexan-1-one (**1e**) (78 mg, 0.25 mmol), (2*S*)-octahydro-1*H*-indole-2-carboxylic acid (**2**) (64 mg, 0.37 mmol) and 6-chloroisatin (**3b**) (45 mg, 0.25 mmol) were reacted according to GP1 for 1 h and yielded white solid spirooxindole (**4l**) (112 mg, 75%); m.p.: 96–97 °C; ^1^H-NMR (500 MHz, CDCl_3_): δ (ppm) = 7.88 (s, 1H, N**H**), 7.47 (d, *J* = 8.0 Hz, 1H, Ar-**H**), 7.35–7.30 (m, 2H, Ar-**H**), 7.20 (s, 1H, Ar-**H**), 7.07 (td, *J* = 5.7, 2.5 Hz, 2H, Ar-**H**), 6.99–6.91 (m, 5H, Ar-**H**), 6.65 (d, *J* = 2.0 Hz, 1H, Ar-**H**), 4.89 (d, *J* = 10.3 Hz, 1H, C**H**), 4.52–4.47 (m, 1H, NC**H**), 3.35 (q, *J* = 4.4 Hz, 1H, NC**H**), 2.93–2.87 (m, 1H, C**H**_2_), 2.79–2.72 (m, 1H C**H**_2_), 2.36–2.30 (m, 1H C**H**_2_), 2.20–2.14 (m, 1H C**H**_2_), 2.07–1.98 (m, 3H C**H**_2_), 1.97–1.92 (m, 1H C**H**_2_), 1.69–1.58 (m, 6H C**H**_2_), 1.07–0.97 (m, 3H C**H**_2_); ^13^C-NMR (126 MHz, CDCl_3_): δ (ppm) = 201.9 (CO), 179.7 (CO), 162.1, 162.0, 159.1, 159.1, 142.3, 137.9, 136.1, 134.9, 134.2, 132.1, 133.1, 132.4, 132.1, 132.0, 131.7, 131.1, 128.4, 128.0, 121.6, 115.7, 115.5, 115.1, 115.0, 110.2, 79.4, 69.5, 64.5, 58.0, 51.6, 42.5, 36.1, 28.5, 28.4, 28.0, 26.6, 24.7, 21.6, 20.4; IR (KBr, cm^−1^) ν_max_ = 2928, 2853, 1715, 1684, 1611, 1584, 1367, 1312, 1273, 1234, 1207, 1151, 1072, 974, 907, 781, 734, 682; (Anal. Calcd. for C_36_H_33_ClF_2_N_2_O_2_: C, 72.17; H, 5.55; N, 4.68; Found: C, 72.05; H, 5.63; N, 4.49); LC/MS (ESI, *m*/*z*): found 599.2 [M(_35_Cl)+H]^+^; 601.2 [M(_37_Cl)+H]^+^, exact mass 598.22 for C_36_H_33_ClF_2_N_2_O_2_. ${\left[\alpha \right]}_{D}^{25}$ = −39.81° (c 0.10, MeOH).

#### 3.1.13. (1. S,1′S,3′S,4a′S,8a′S,9a′R)-6″-Chloro-3-((E)-4-(trifluoromethyl)benzylidene)-1′-(4-(trifluoromethyl)phenyl)-4a′,5′,6′,7′,8′,8a′,9′,9a′-octahydro-1′H-dispiro[cyclohexane-1,2′-pyrrolo[1,2-a]indole-3′,3″-indoline]-2,2″-dione (**4m**)

2,6-*Bis*((*E*)-4-(trifluoromethyl)benzylidene)cyclohexan-1-one (**1f**) (103 mg, 0.25 mmol), (2*S*)-octahydro-1*H*-indole-2-carboxylic acid (**2**) (64 mg, 0.37 mmol) and 6-chloroisatin (**3b**) (45 mg, 0.25 mmol) were reacted according to GP1 for 1 h and yielded white solid spirooxindole (**4m**) (108 mg, 62%); m.p.: 78–79 °C; ^1^H-NMR (500 MHz, CDCl_3_): δ (ppm) = 8.49 (S, 1H, N**H**), 7.67–7.61 (m, 1H, Ar-**H**), 7.55 (d, *J* = 7.3 Hz, 3H, Ar-**H**), 7.45–7.27 (m, 4H, Ar-**H**), 7.16 (d, *J* = 8.0 Hz, 2H, Ar-**H**), 6.99 (t, *J* = 6.9 Hz, 1H, Ar-**H**), 6.78 (q, *J* = 3.4, 2.9 Hz, 1H, Ar-**H**), 5.02 (d, *J* = 10.4 Hz, 1H, C**H**), 4.64–4.54 (m, 1H, NC**H**), 3.36 (q, *J* = 3.9 Hz, 1H, NC**H**), 2.87–2.74 (m, 1H, C**H**_2_), 2.38–2.28 (m, 1H, C**H**_2_), 2.22–2.14 (m, 1H, C**H**_2_), 2.13–2.00 (m, 2H, C**H**_2_), 1.99–1.92 (m, 1H, C**H**_2_), 1.74–1.57 (m, 4H, C**H**_2_), 1.55–1.47 (m, 2H, C**H**_2_), 1.45–1.36 (m, 2H, C**H**_2_), 1.06–0.90 (m, 3H, C**H**_2_); ^13^C-NMR (126 MHz, CDCl_3_): δ (ppm) = 201.88 (**C**O), 179.9 (**C**O), 142.5, 141.8, 140.3, 135.8, 135.2, 133.5, 131.0, 130.6, 130.5, 130.2, 130.1, 129.8, 128.2, 128.1, 125.3 (**C**F_3_), 125.1 (**C**F_3_), 121.8, 110.5, 79.7, 69.4, 64.3, 58.2, 51.8, 42.5, 36.2, 28.5, 28.3, 27.9, 26.7, 24.6, 21.5, 20.3; IR (KBr, cm^−1^) ν_max_ = 2927, 2856, 1712, 1683, 1615, 1587, 1364, 1316, 1274, 1232, 1205, 1155, 1078, 977, 908, 789, 735, 684; (Anal. Calcd. for C_38_H_33_ClF_6_N_2_O_2_: C, 65.28; H, 4.76; N, 4.01; Found: C, 65.33; H, 4.62; N, 4.13); LC/MS (ESI, *m*/*z*): found 699.2 [M(_35_Cl)+H]^+^; 701.2 [M(_37_Cl)+H]^+^, exact mass 698.21 for C_38_H_33_ClF_6_N_2_O_2_. ${\left[\alpha \right]}_{D}^{25}$ = −34.59° (c 0.15, MeOH).

#### 3.1.14. (1. S,1′S,3′S,4a′S,8a′S,9a′R)-5″-Methyl-3-((E)-4-(trifluoromethyl)benzylidene)-1′-(4-(trifluorom-thyl)phenyl)-4a′,5′,6′,7′,8′,8a′,9′,9a′-octahydro-1′H-dispiro[cyclohexane-1,2′-pyrrolo[1,2-a]indole-3′,3″-indoline]-2,2″-dione (**4n**)

2,6-*Bis*((*E*)-4-(trifluoromethyl)benzylidene)cyclohexan-1-one (**1f**) (103 mg, 0.25 mmol), (2*S*)-octahydro-1*H*-indole-2-carboxylic acid (**2**) (64 mg, 0.37 mmol) and 5-methylisatin (**3f**) (40 mg, 0.25 mmol) were reacted according to GP1 for 1 h and yielded white solid spirooxindole (**4n**) (102 mg, 60%); m.p.: 73–74 °C; ^1^H-NMR (500 MHz, CDCl_3_): δ (ppm) = 8.42 (s, 1H, N**H**), 7.62 (d, *J* = 8.1 Hz, 1H, Ar-**H**), 7.57 (d, *J* = 2.6 Hz, 1H, Ar-**H**), 7.48 (d, *J* = 8.1 Hz, 2H, Ar-**H**), 7.43 (s, 1H, Ar-**H**), 7.40 (d, *J* = 8.0 Hz, 1H, Ar-**H**), 7.35 (d, *J* = 9.1 Hz, 1H, Ar-**H**), 7.29 (d, *J* = 12.5 Hz, 1H, Ar-**H**), 7.24 (d, *J* = 8.1 Hz, 1H, Ar-**H**), 7.14 (d, *J* = 8.1 Hz, 2H, Ar-**H**), 6.61 (d, *J* = 8.1 Hz, 1H, Ar-**H**), 5.07 (d, *J* = 10.4 Hz, 1H, C**H**), 4.62–4.56 (m, 1H, NC**H**), 3.37–3.33 (m, 1H, NC**H**), 2.96–2.86 (m, 1H, C**H**_2_), 2.35 (s, 3H, C**H**_3_), 1.99–1.93 (m, 1H, C**H**_2_), 1.89–1.85 (m, 1H, C**H**_2_), 1.73–1.68 (m, 1H, C**H**_2_), 1.62–1.54 (m, 4H, C**H**_2_), 1.50–1.39 (m, 3H, C**H**_2_), 1.16–1.07 (m, 2H, C**H**_2_), 1.03–0.91 (m, 3H, C**H**_2_), 0.89–0.83 (m, 1H, C**H**_2_); ^13^C-NMR (126 MHz, CDCl_3_): δ (ppm) = 202.0 (**C**O), 180.2 (**C**O), 142.2, 140.6, 139.3, 138.8, 134.6, 132.7, 131.9, 131.0, 130.7, 130.5, 130.2, 130.1, 129.8, 129.7, 128.1, 125.2 (**C**F_3_), 125.0 (**C**F_3_), 109.4, 80.1, 69.1, 64.3, 58.3, 42.4, 36.2, 28.5, 28.3, 27.7, 26.4, 24.6, 21.6, 21.4 (**C**H_3_), 20.3; IR (KBr, cm^−1^) ν_max_ = 2926, 2852, 1714, 1687, 1615, 1582, 1363, 1315, 1272, 1234, 1207, 1157, 1075, 979, 904, 785, 737, 685; (Anal. Calcd. for C_39_H_36_F_6_N_2_O_2_: C, 69.02; H, 5.35; N, 4.13; Found: C, 69.25; H, 5.17; N, 4.01); LC/MS (ESI, *m*/*z*): found 679.3 [M+H]^+^; exact mass 678.27 for C_39_H_36_F_6_N_2_O_2_. ${\left[\alpha \right]}_{D}^{25}$ = −12.18° (c 0.14, MeOH).

### 3.2. The Biological Activity Assay Protocols

Cytotoxicity against BJ human fibroblast cells and cytotoxicity against PC3, HeLa, MCF-7, and MDA-MB231 cancer cell lines were evaluated by following the procedure as described in the literature [41,42,43,44,45,46,47] (Supplementary Materials).

### 3.3. Molecular Docking Methodology

To rationalize the observed anticancer activity, molecular docking studies of the potential compounds were carried out using MDM2 crystal structure. The selected di-spirooxindole analogs (**4a, 4b, 4i**, and **4l**) were sketched in MOE v.2019 by using Builder module and subsequently subjected to geometry correction and protonation followed by minimization using MMFF94x force field. The 3D structure of MDM2 in complex with a benzodiazepine inhibitor (PDB ID 1T4E) was retrieved from ProteinData Bank [34]. Since there were no conserved water molecules in the utilized pdb, all water molecules were deleted and the structure was corrected and protonated using GB/VI as the electrostatics function with a dielectric value of 80 (for solvent). Consequently, the protein structure was minimized to remove the bad clashes using the Amber99 force field. A grid of 6 Å was generated centered on the co-crystalized ligands and the selected compounds. The triangular method was used as a placement method with an induce fit protocol. The resulting poses of the ligands were scored by London dG scoring function and the top ranked poses were visually analyzed. All the graphics were rendered using MOE software.

## 4. Conclusions

In conclusion, a new series of hybrid di-spirooxindole analogs, engrafted with substituted oxindole and cyclohexanone moieties, were synthesized successfully by a one-pot multicomponent reaction. The anticancer assay showed promising results, which makes these di-spirooxindole analogs suitable for further research. Synthesized di-spirooxindole analog **4b** (IC_50_ = 3.7 ± 1.0 µM) appeared to be a more potent candidate against PC3 cell line, whereas, di-spirooxindole analogs **4a** (IC_50_ = 7.1 ± 0.2 µM) and **4l** (IC_50_ = 7.2 ± 0.5 µM) possessed promising anticancer activity against cervical cancer HeLa cell line and triple negative breast cancer MDA-MB231 cell line. Compound **4i** (IC_50_ = 7.63 ± 0.08 µM) appeared to be more active among these di-spirooxindole analogs. The docking studies suggested that **4a, 4b, 4i**, and **4l** accommodated well in the binding site of MDM2. However, further mechanistic studies via in vivo animal models are required to validate the results of these in vitro assays.

## Figures and Tables

**Table 1:** Results of anticancer assay against BJ, PC3, HeLa, MCF-7, and MDA-MB231 cells.

≠	Chemical Structure 4a-n	Cancer Type/Cell Line (IC_50_, µM) ^a,b^				
		Human Fibroblast BJ	Prostate PC3	Cervical HeLa	Breast MCF-7	Breast MDA-MB231
**4a**	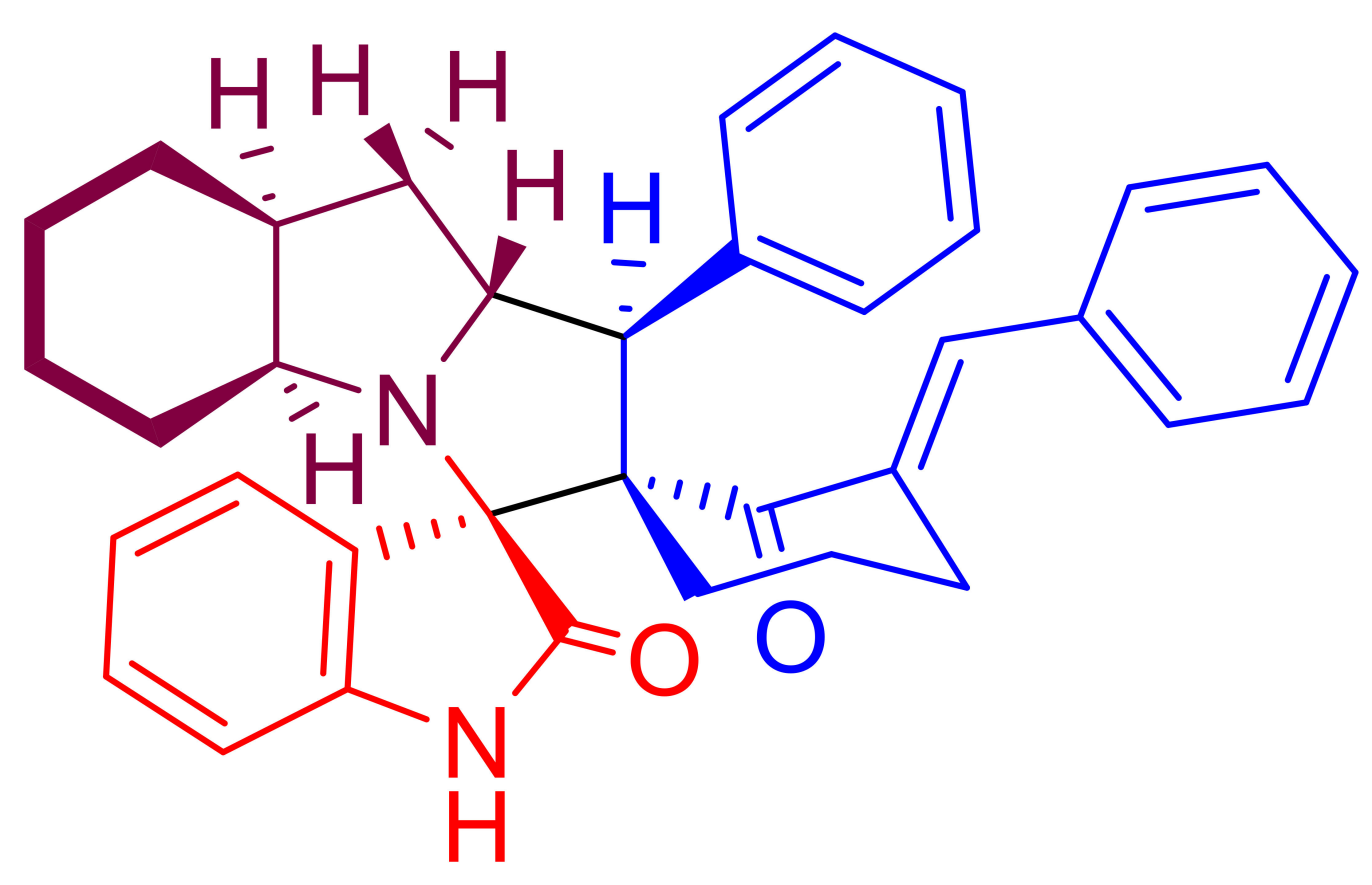	NA	24.1 ± 1.1	7.1 ± 0.2	25.04 ± 0.57	19.50 ± 0.56
**4b**	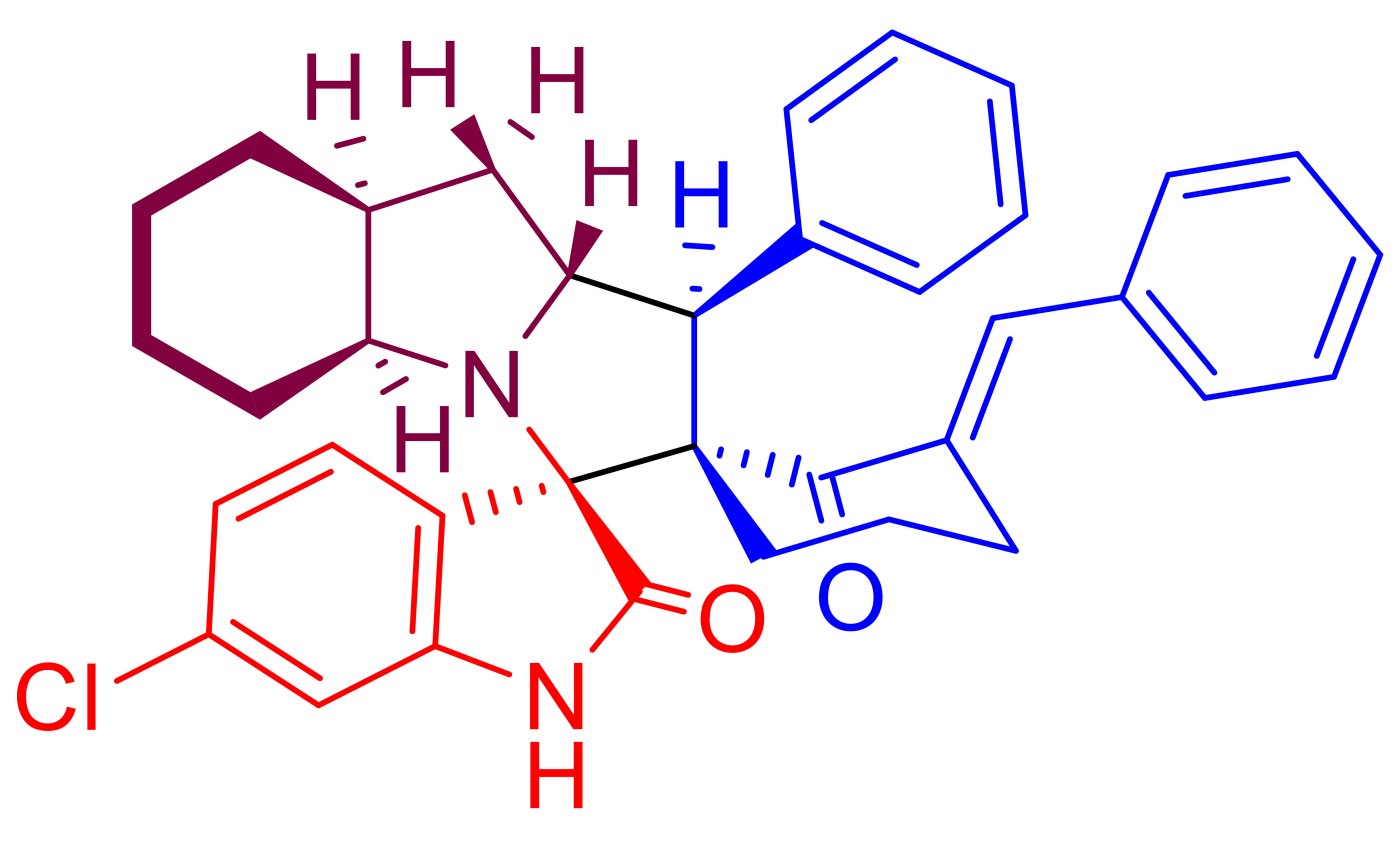	NA	3.7 ± 1.0	NA	27.72 ± 0.59	24.08 ± 0.02
**4c**	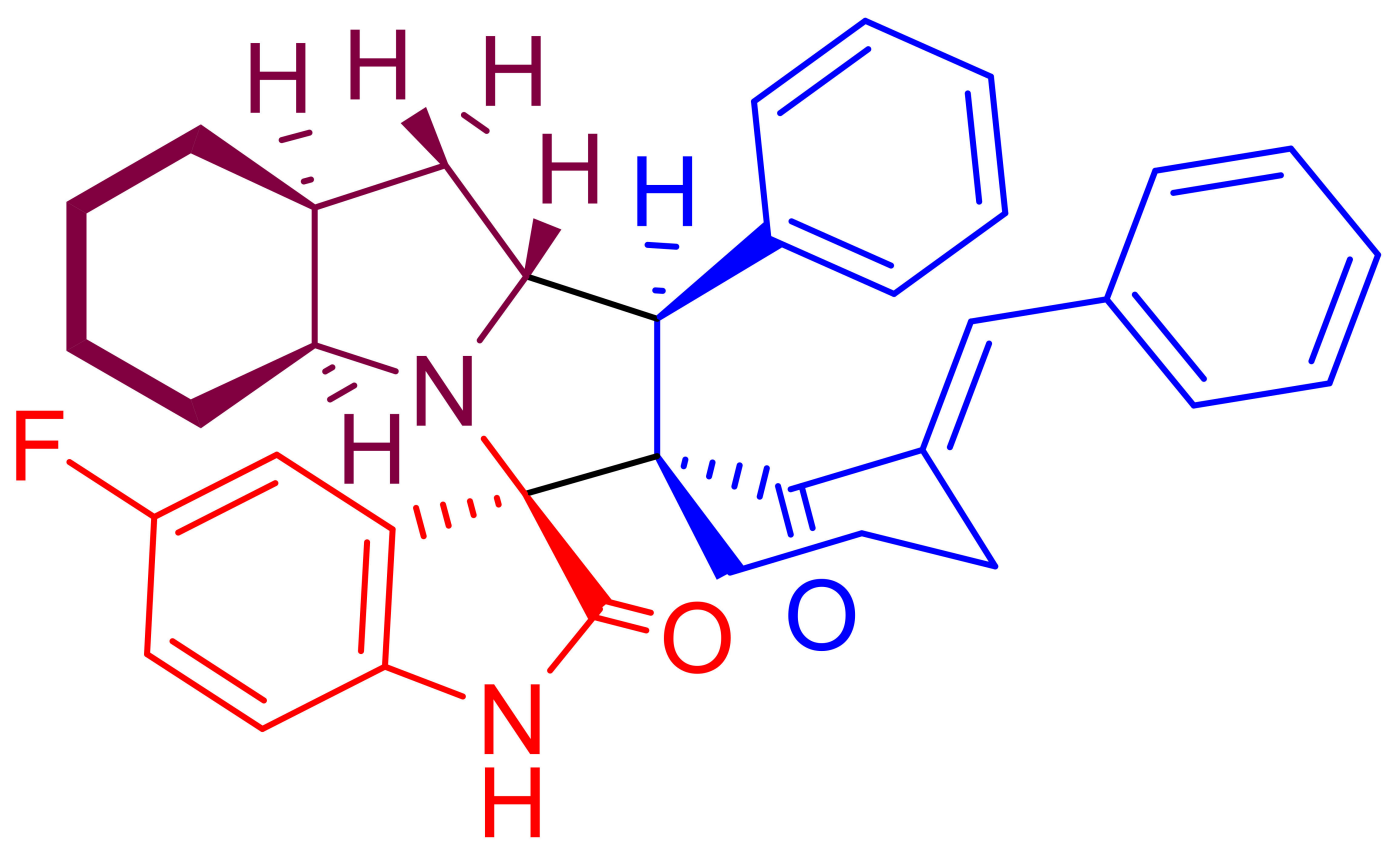	NA	17.9 ± 0.2	NA	27.82 ± 1.02	20.62 ± 2.16
**4d**	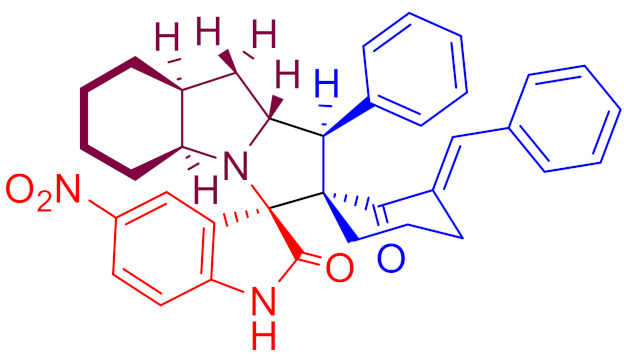	NA	29.8 ± 0.1	NA	NA	NA
**4e**	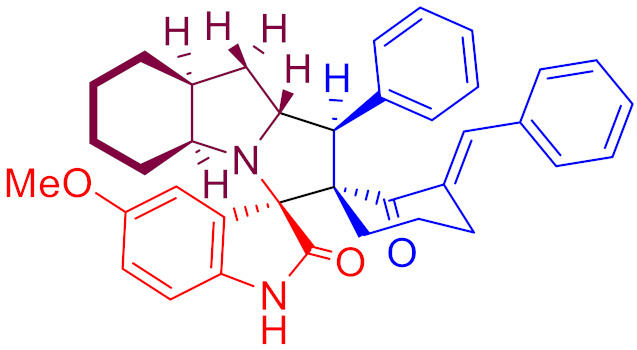	NA	19.6 ± 1.2	26.5 ± 0.04	NA	NA
**4f**	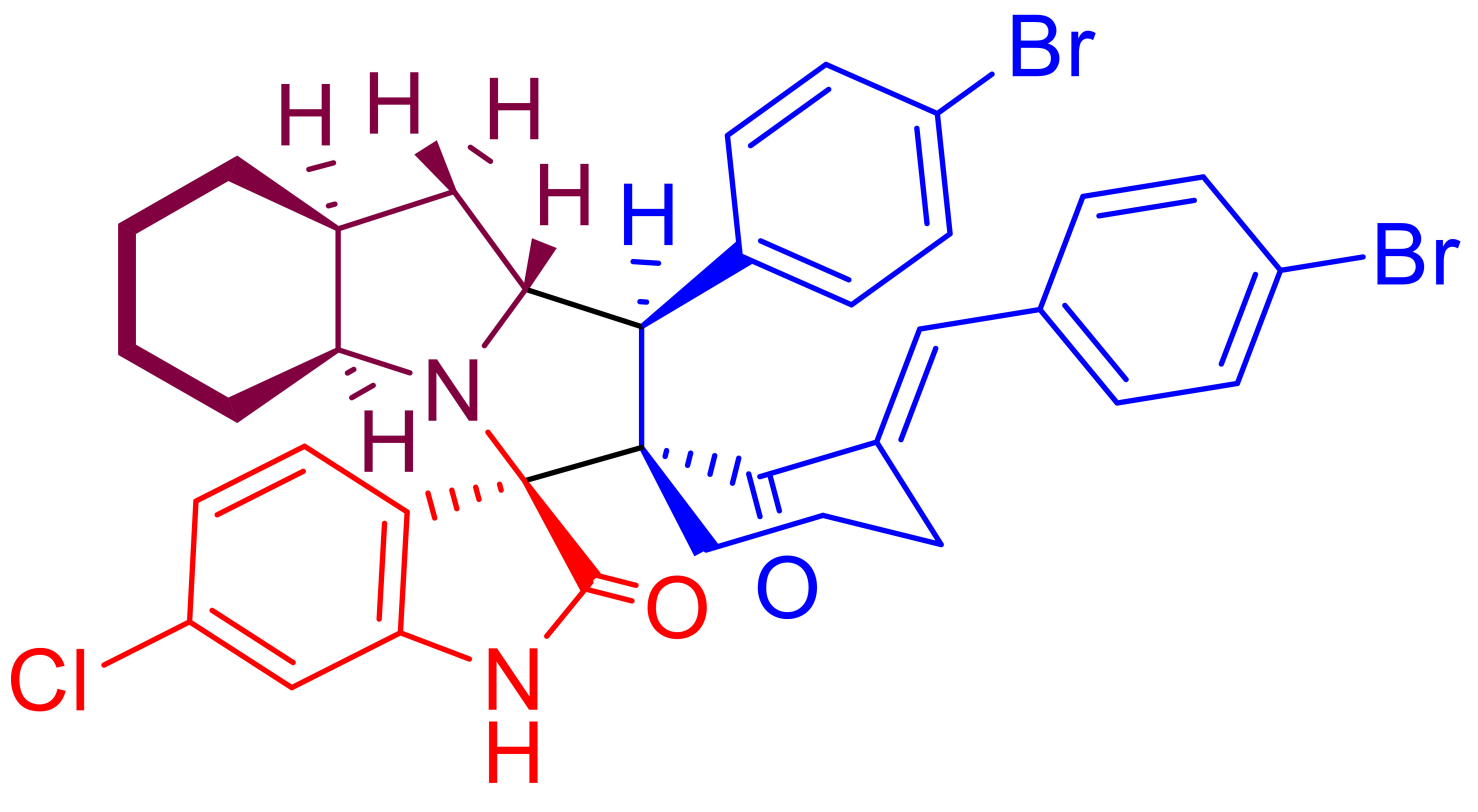	NA	NA	NA	NS ^c^	NS
**4g**	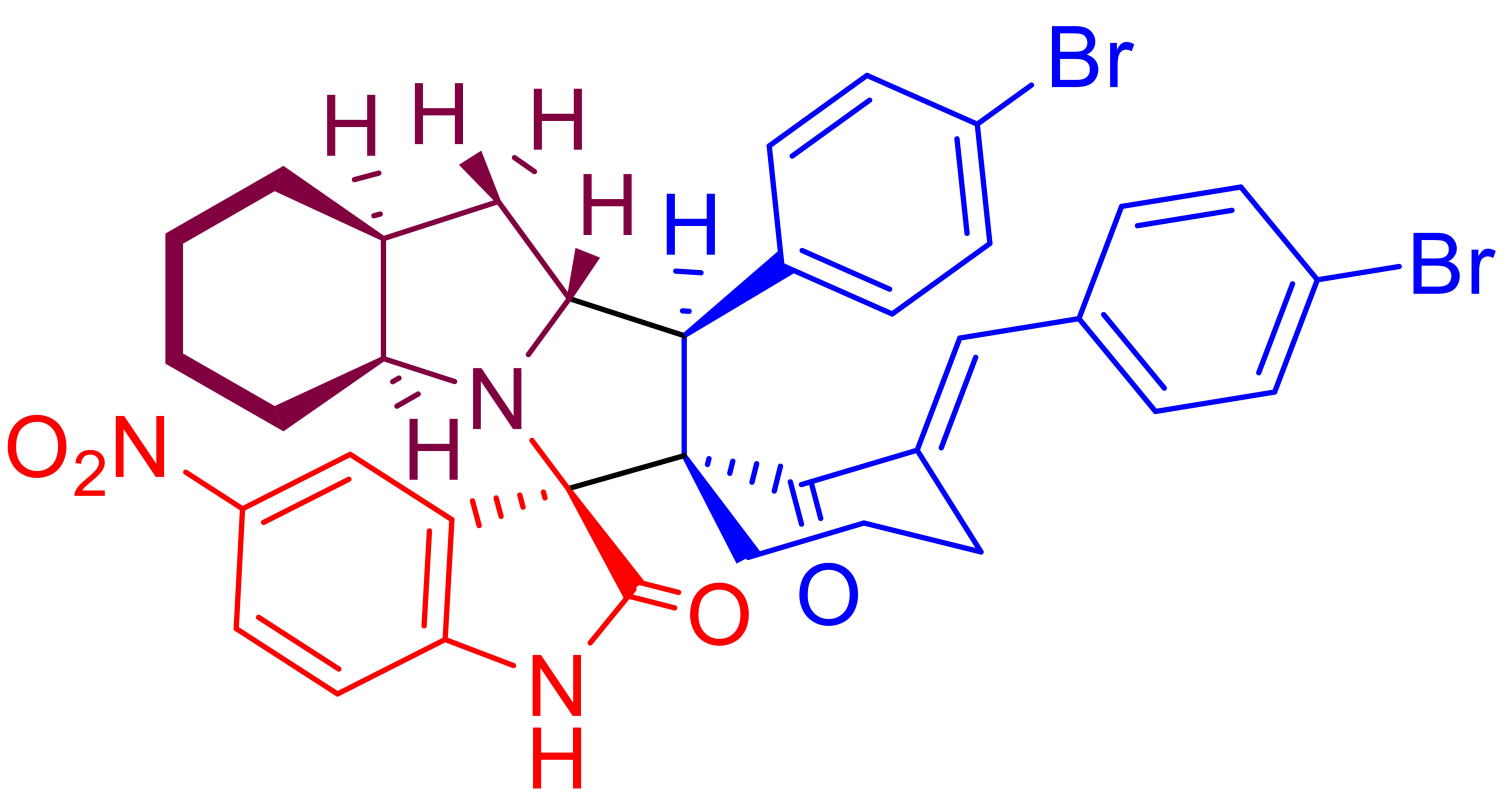	21.7 ± 0.2	14.3 ± 1.0	NA	NA	NS
**4h**	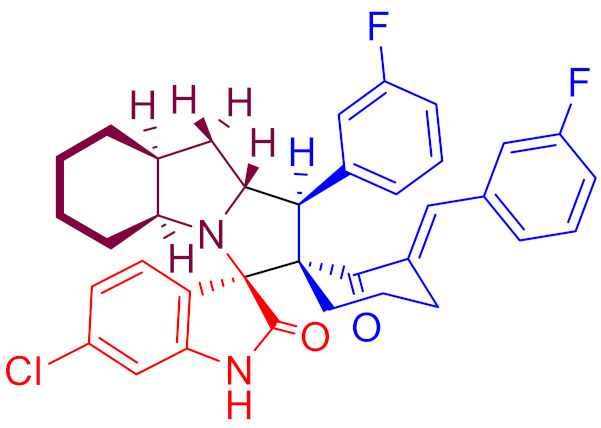	NA	NA	NA	NA	14.43 ± 0.09
**4i**	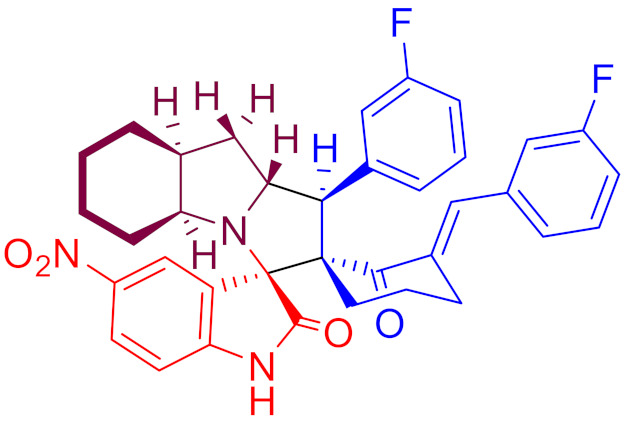	NA	NA	NA	NA	7.63 ± 0.08
**4j**	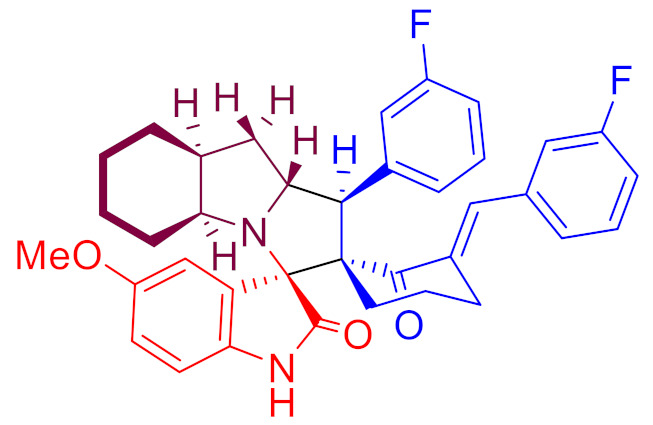	NA	NA	11.9 ± 0.04	NA	10.49 ± 0.71
**4k**	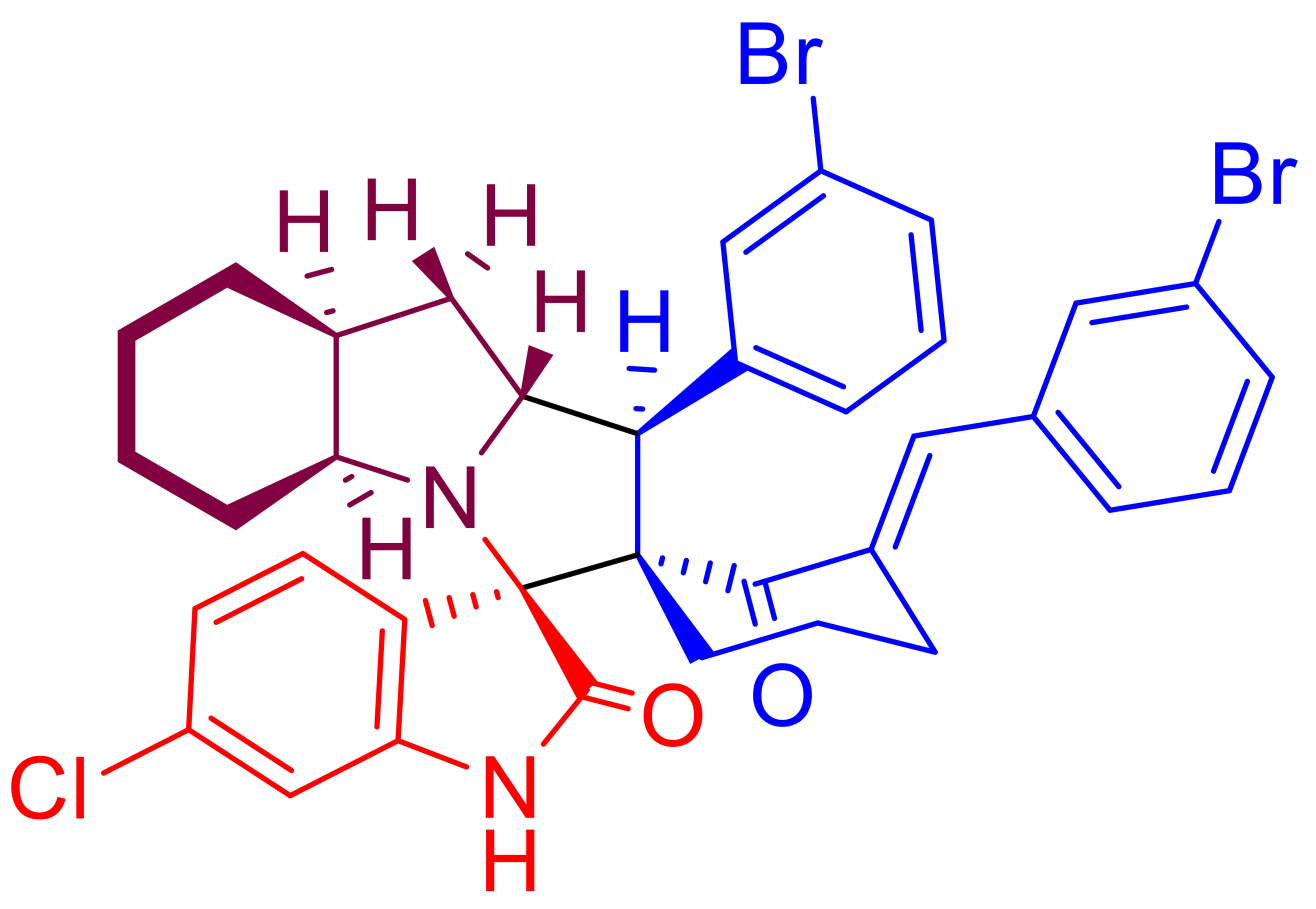	NA	NA	NA	NA	NA
**4l**	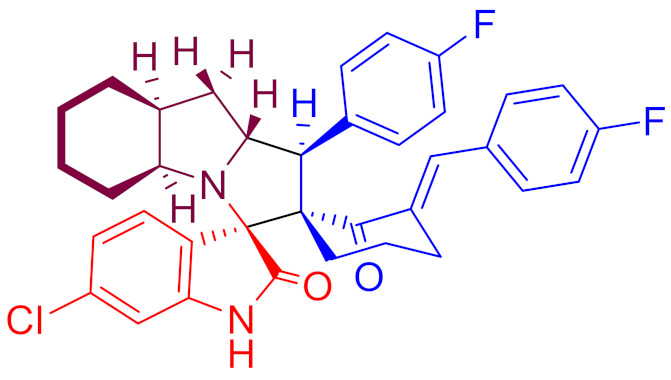	NA	NA	7.2 ± 0.5	NA	14.45 ± 0.08
**4m**	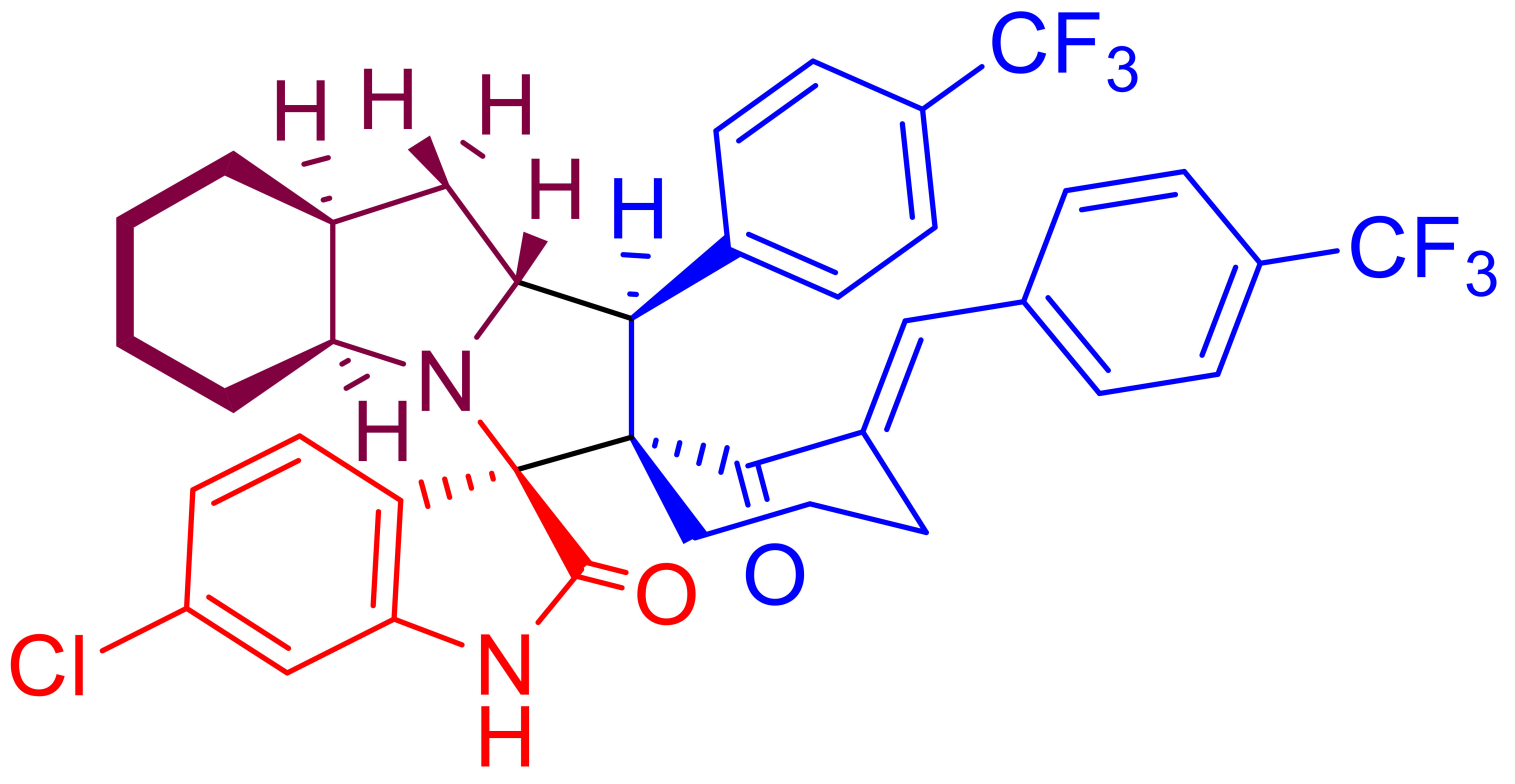	NA	NA	24.6 ± 0.4	NA	NA
**4n**	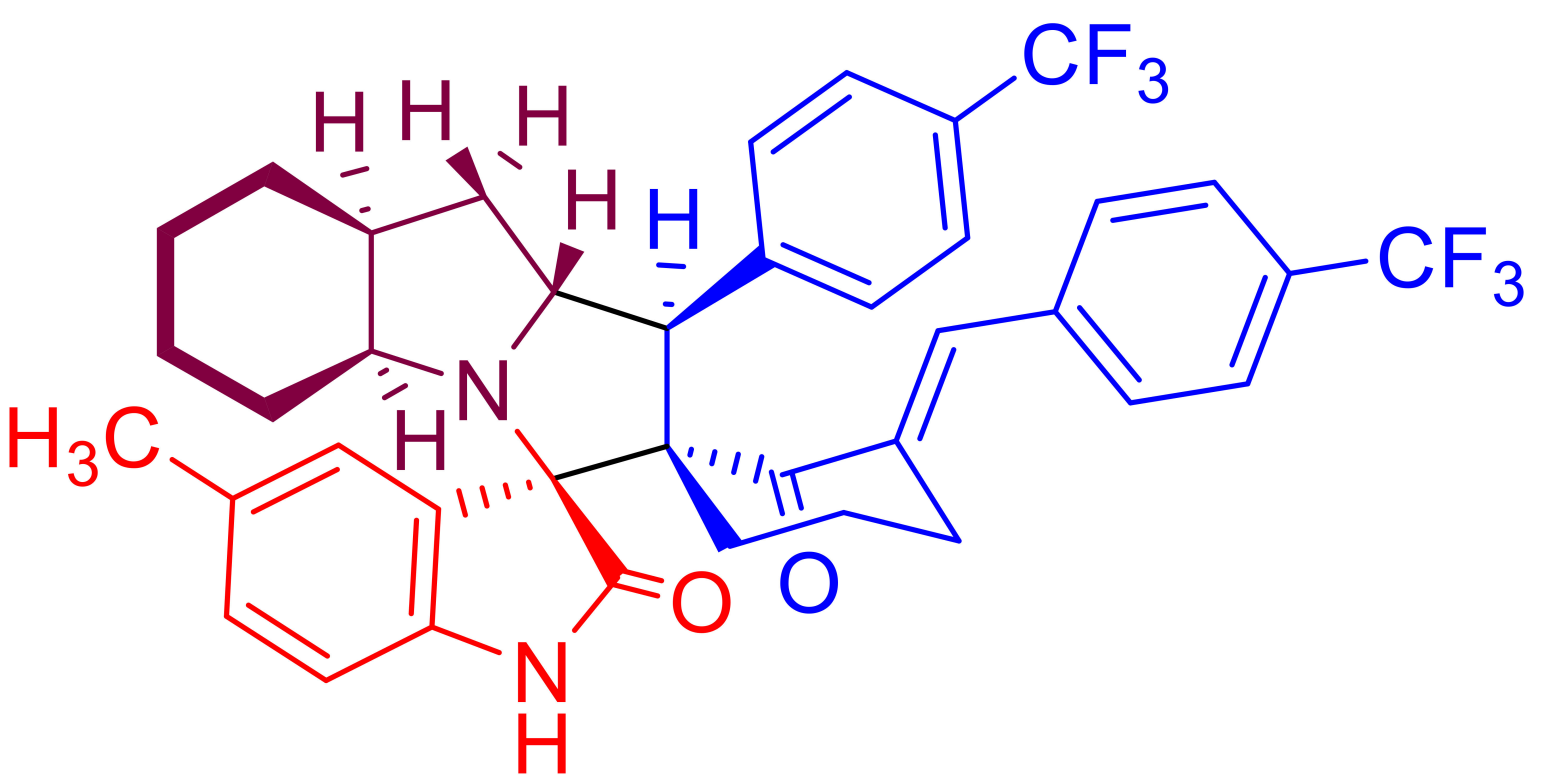	NA	NA	NA	NA	NA
STD.	Doxorubicin	NA	1.9 ± 0.4	0.9 ± 0.14	0.79 ± 0.05	0.32 ± 0.002

^a^ IC_50_ (μM) was evaluated using MTT assay and ± is the standard deviation from three independent experiments. ^b^ NA means that the tested compound did not show anticancer activity at 30 µM. ^c^ NS: Not soluble.

## Data Availability

The data presented in this study are available in Supplementary Materials.

## Supplementary Materials

The following are available online, 1D-NMR and 2D-NMR chemical shift data for compounds **4a–n** and the biological activity assays protocols are provided in SI.

## Sample Availability

Samples of the compounds **4a–n** are available from the authors.
