# Supplementary material for: Stereoselective Synthesis of the Di-Spirooxindole Analogs Based Oxindole and Cyclohexanone Moieties as Potential Anticancer Agents
Source: Molecules. 2021 Oct 19;26(20):6305. doi: 10.3390/molecules26206305 (PMC8541513; doi:10.3390/molecules26206305)
Supplement: Supplementary file 1 [file molecules-26-06305-s001.zip › molecules-1288807-supplementary.pdf]

# Supporting Information

# Stereoselective Synthesis of the Di-Spirooxindole Analogs Based Oxindole and Cyclohexanone Moieties as Potential Anticancer Agents

**Abdullah Mohammed Al-Majid [1], M. Ali [1], Mohammad Shahidul Islam [1], Saeed Alshahrani [1], Abdullah Saleh Alamary [1], Sammer Yousuf [2], M. Iqbal Choudhary[2], and Assem Barakat [1,3,*]**

[1] Department of Chemistry, College of Science, King Saud University, P. O. Box 2455, Riyadh 11451, Saudi Arabia; Emails: amajid@ksu.edu.sa (A.M.A.-M.); maly.c@ksu.edu.sa (M.A.); mislam@ksu.edu.sa (M.S.I.); chemistry99y@gmail.com (S.A.); alamary1401@yahoo.com (A.S.A.).
[2] H.E.J. Research Institute of Chemistry, International Center for Chemical and Biological Sciences, University of Karachi, Karachi 75270, Pakistan; dr.sammer.yousuf@gmail.com (S.Y.); iqbal.choudhary@iccs.edu (M.I.C.)
[3] Department of Chemistry, Faculty of Science, Alexandria University, P.O. Box 426, Ibrahimia, Alexandria 21321, Egypt.
[*] Correspondence: ambarakat@ksu.edu.sa; Tel.: +966-11467-5901; Fax: +966-11467-5992

# NMR SPECTRA

**Figure S1:** $^{1}$H-NMR and $^{13}$C-NMR for compound-**4a**

AB1171_1H

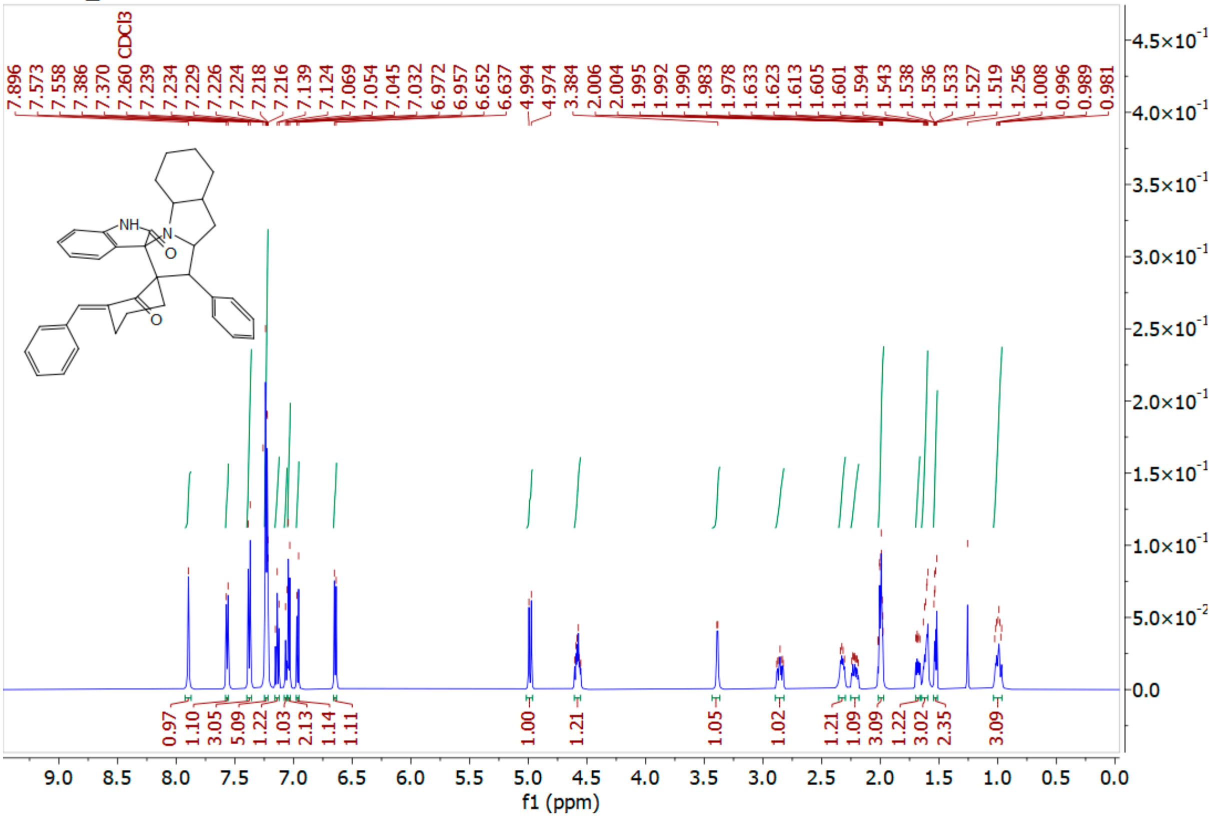


AB1171_13C

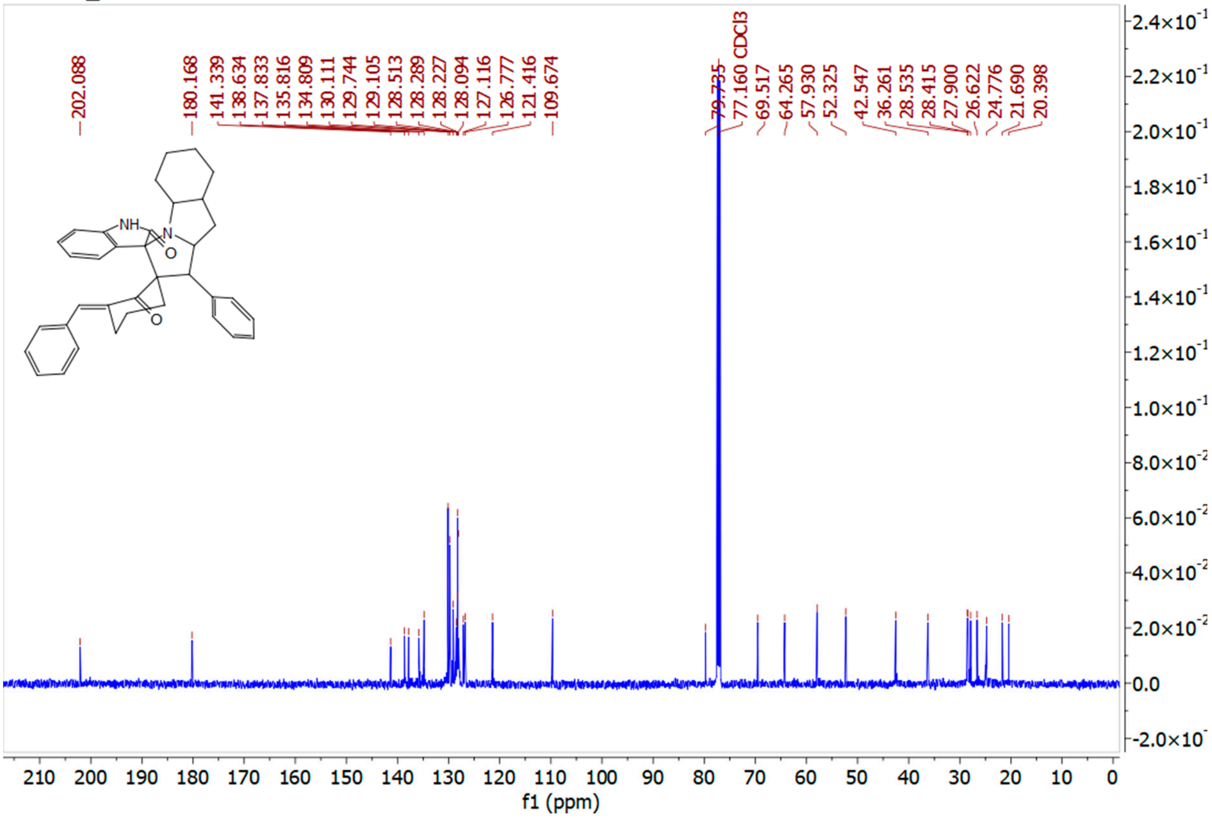

**Figure S2:** $^{1}$H-NMR and $^{13}$C-NMR for compound-**4b**

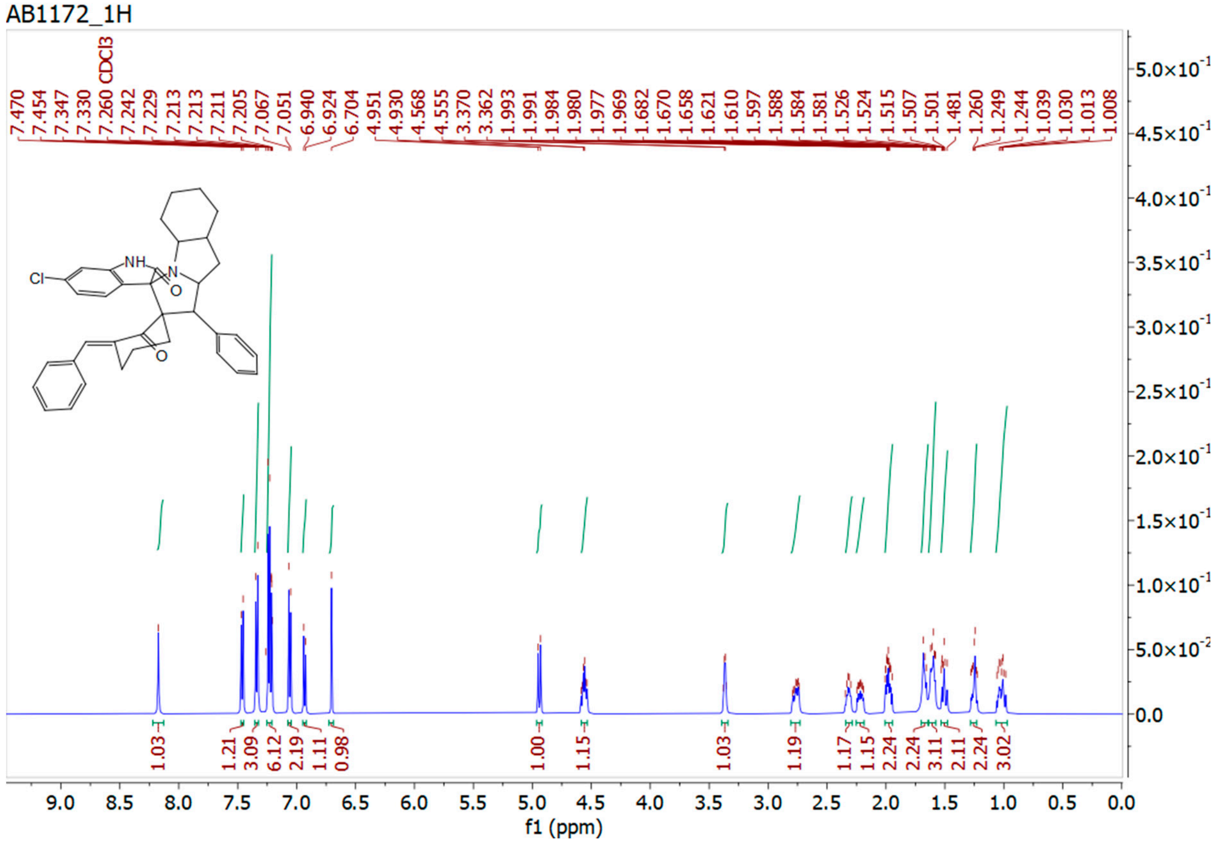


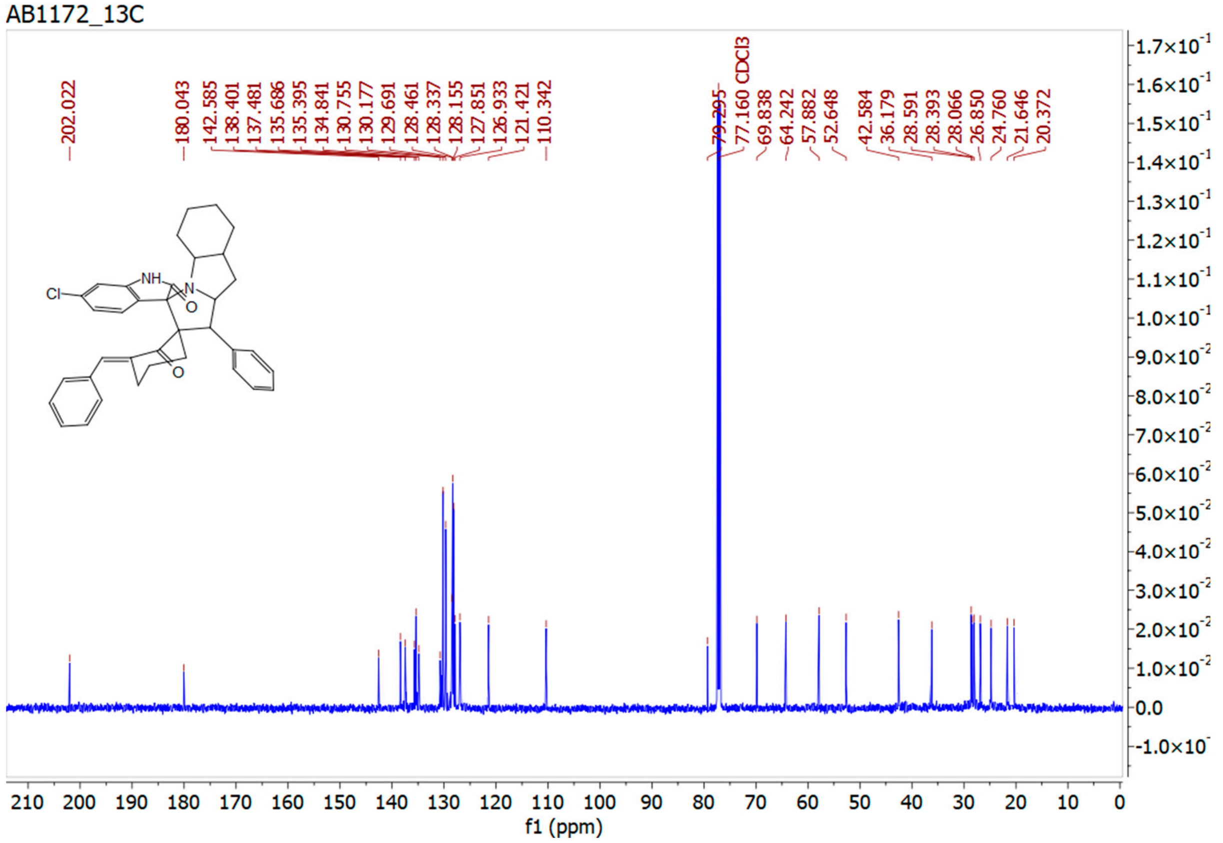

**Figure S3:** $^{1}$H-NMR and $^{13}$C-NMR for compound-**4c**

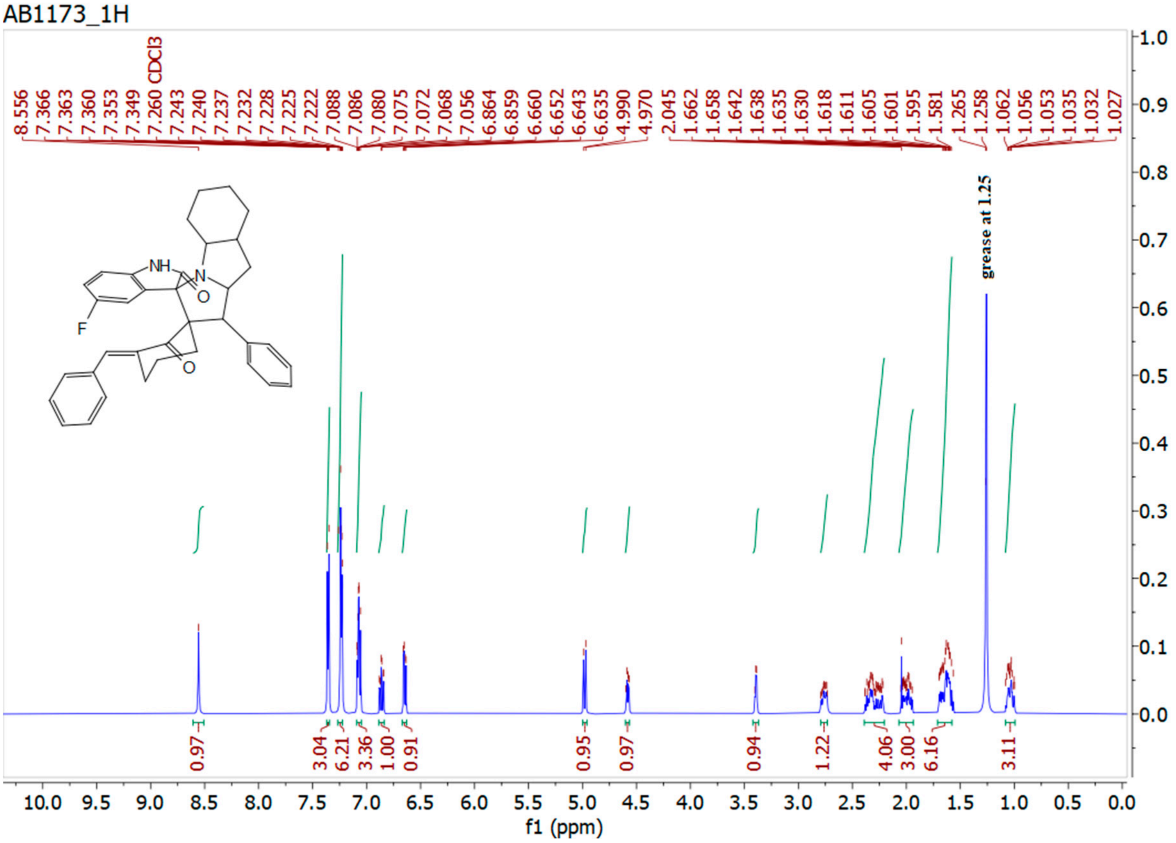


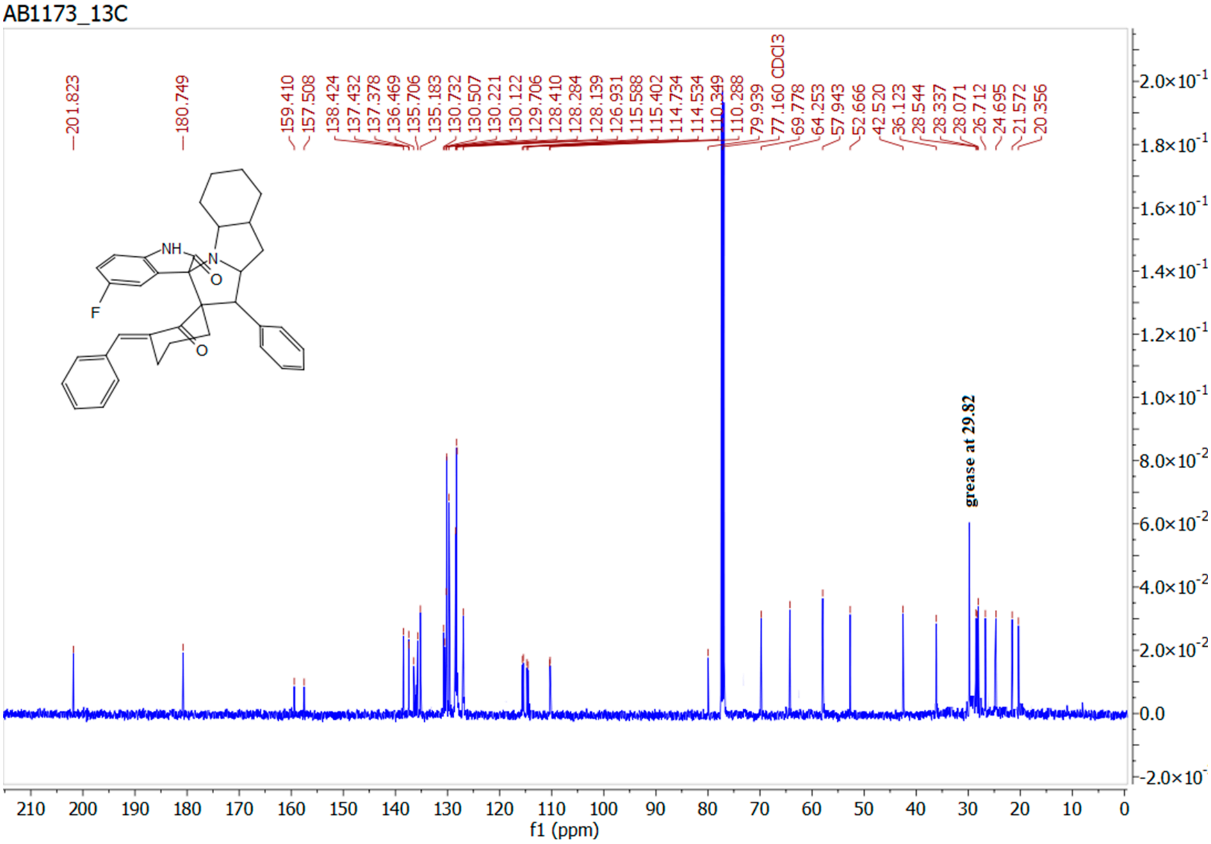

**Figure S4:** $^{1}$H-NMR and $^{13}$C-NMR for compound-**4d**

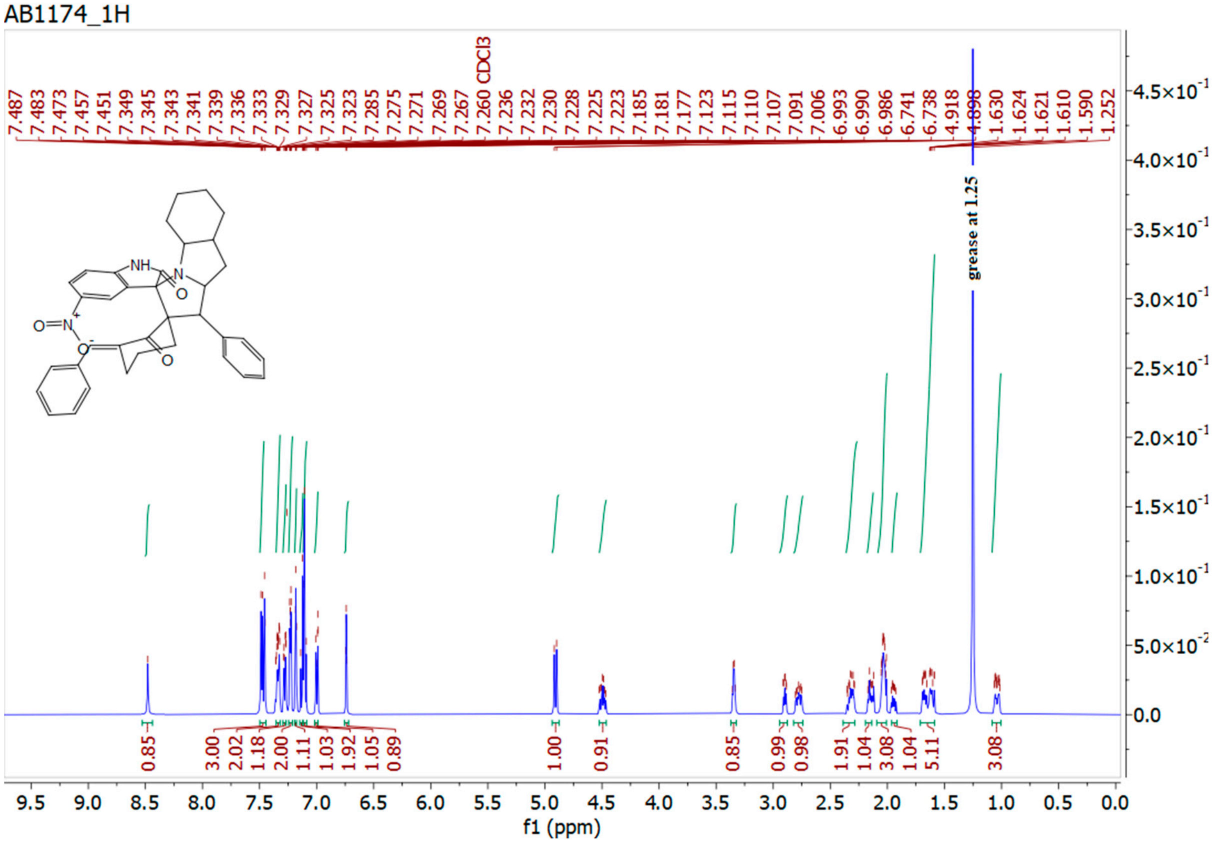


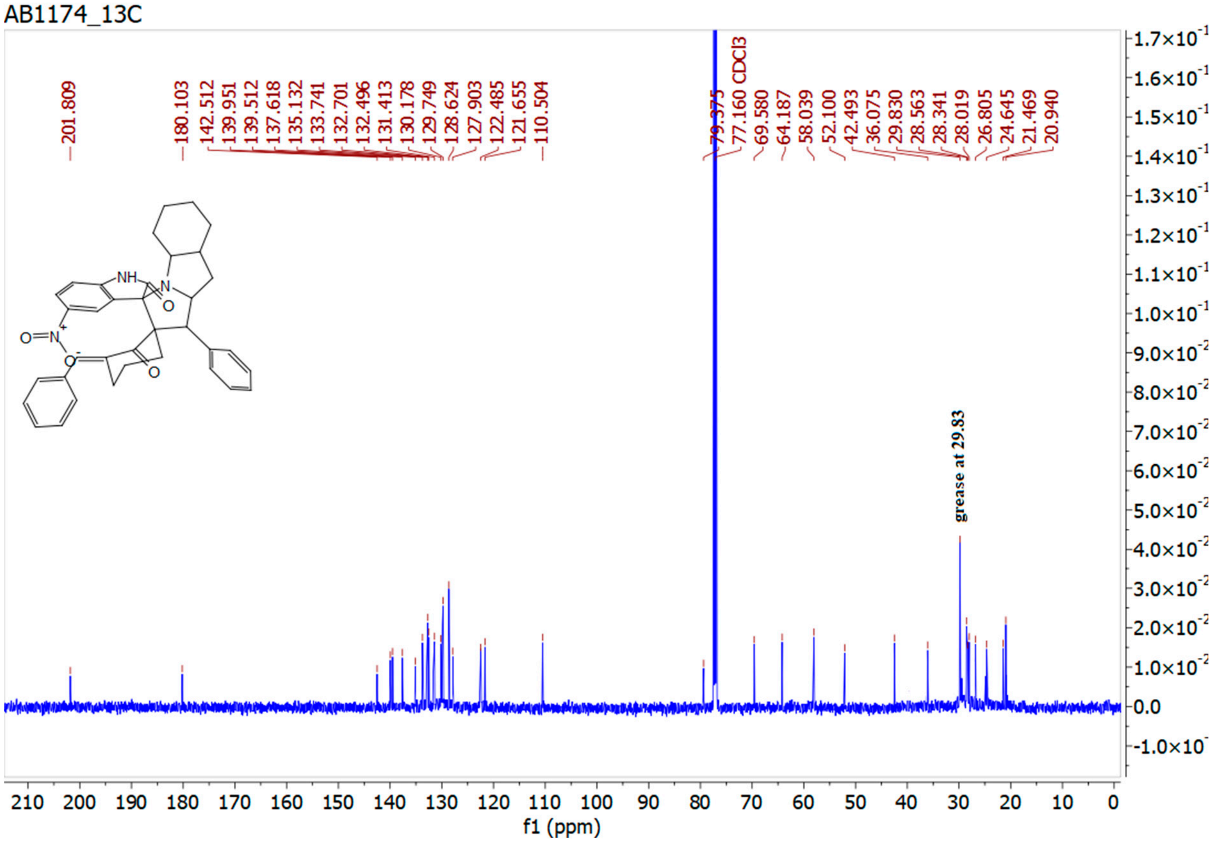

**Figure S5:** $^{1}$H-NMR and $^{13}$C-NMR for Compound-**4e**

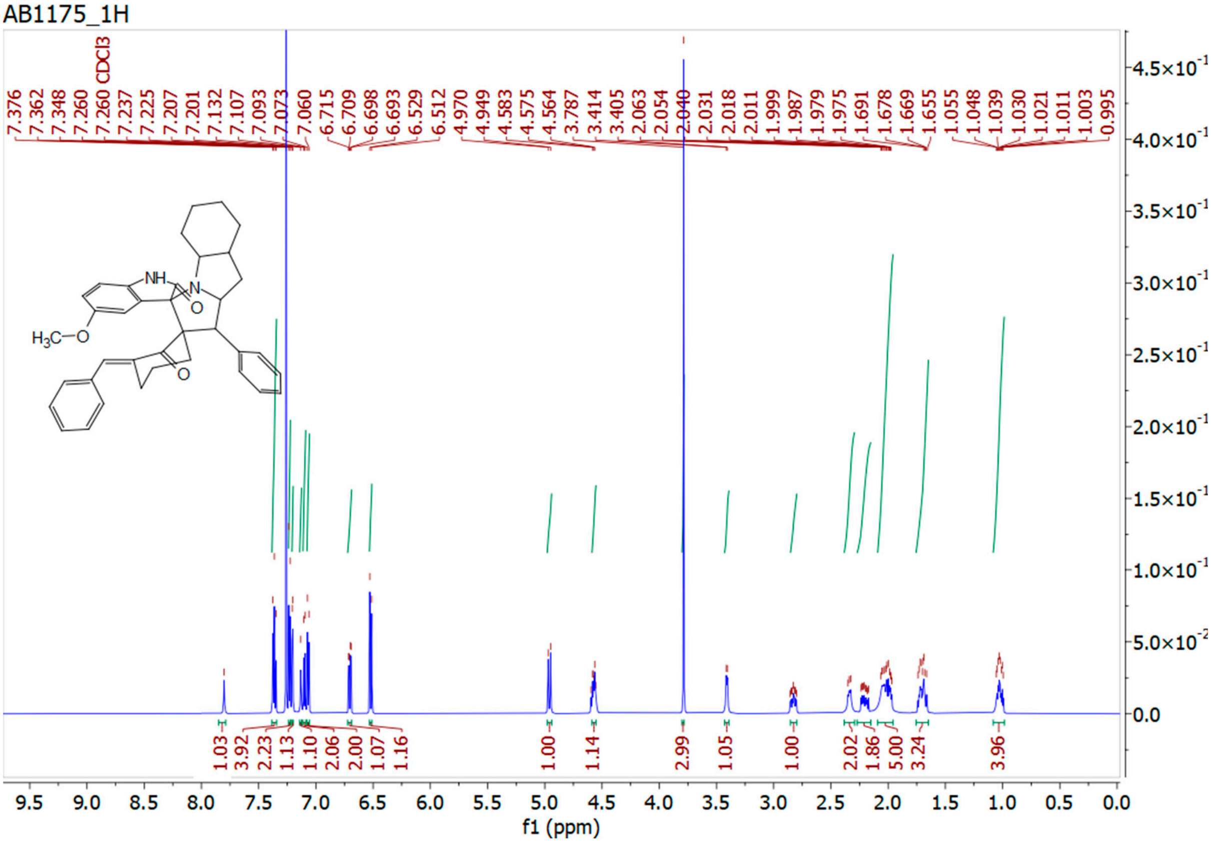


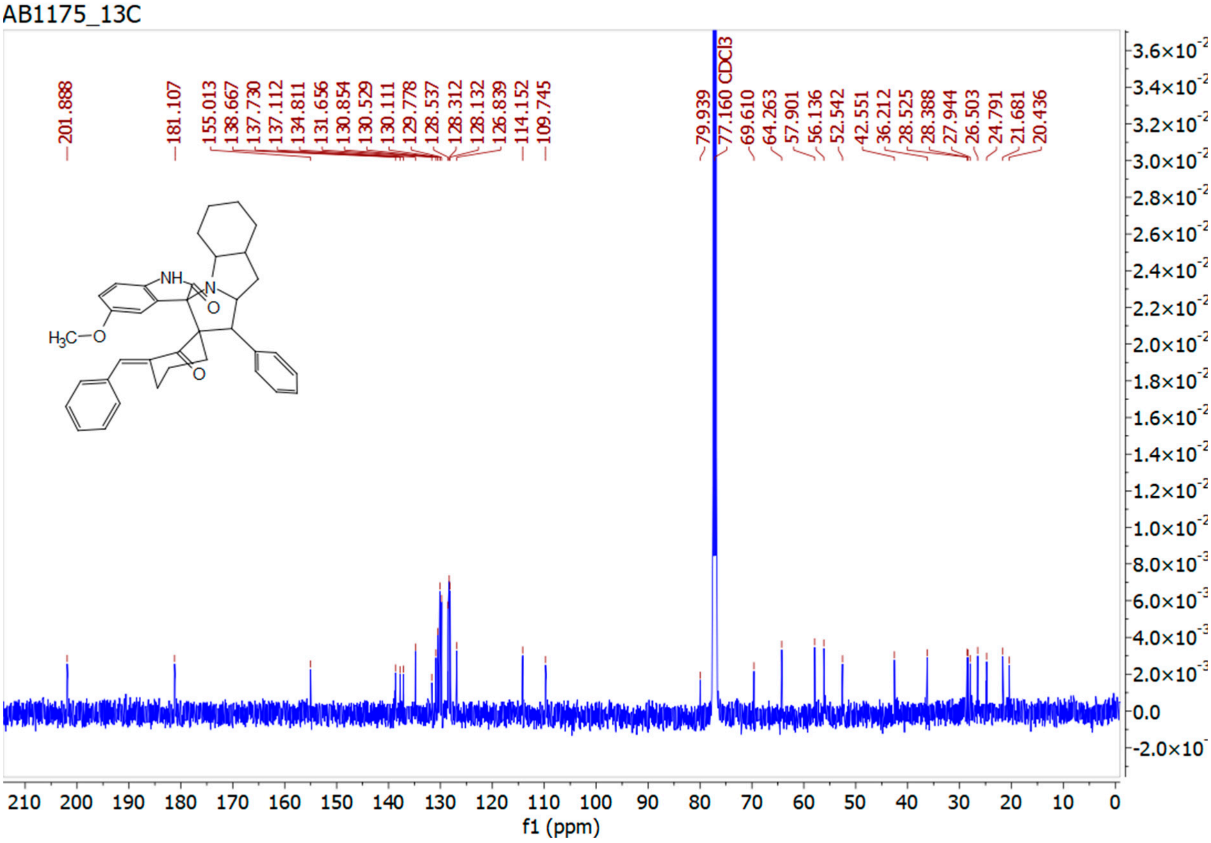

**Figure S6:** $^{1}$H-NMR and $^{13}$C-NMR for compound-**4f**

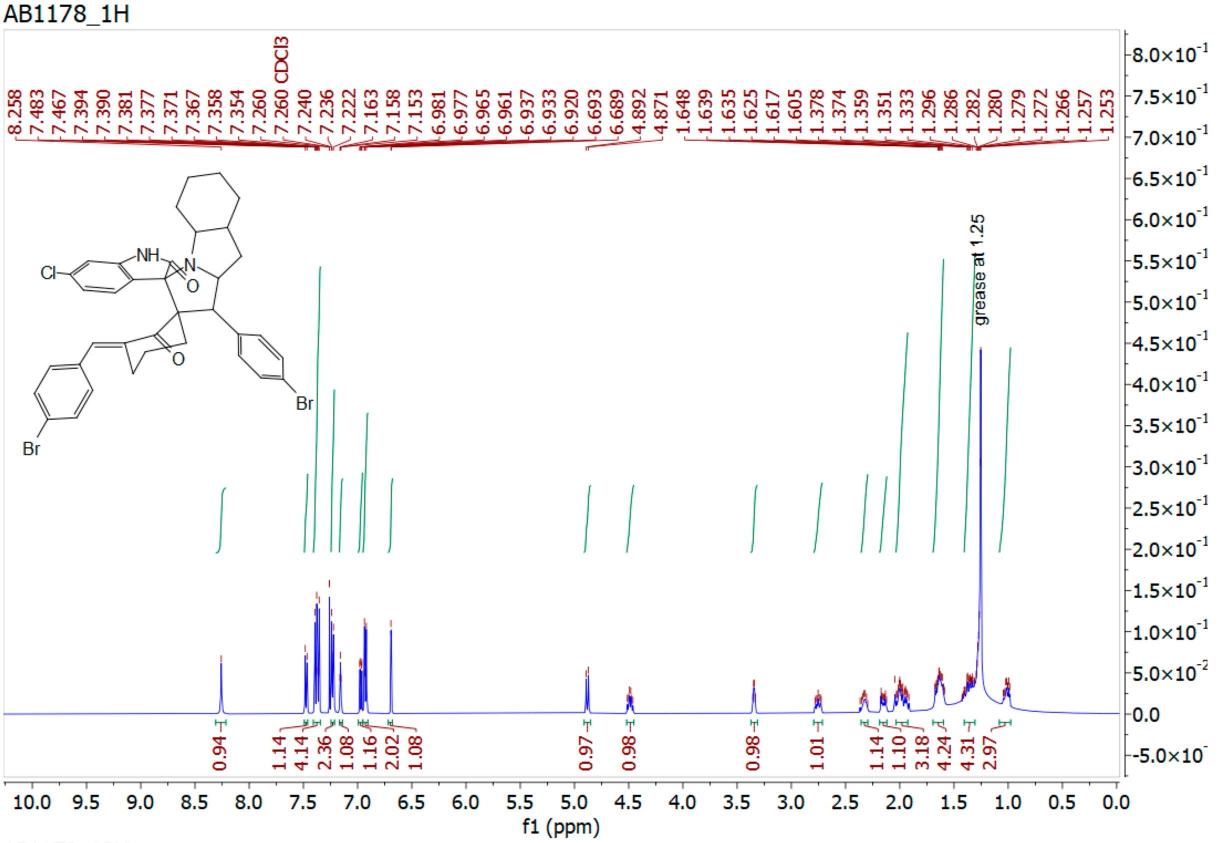


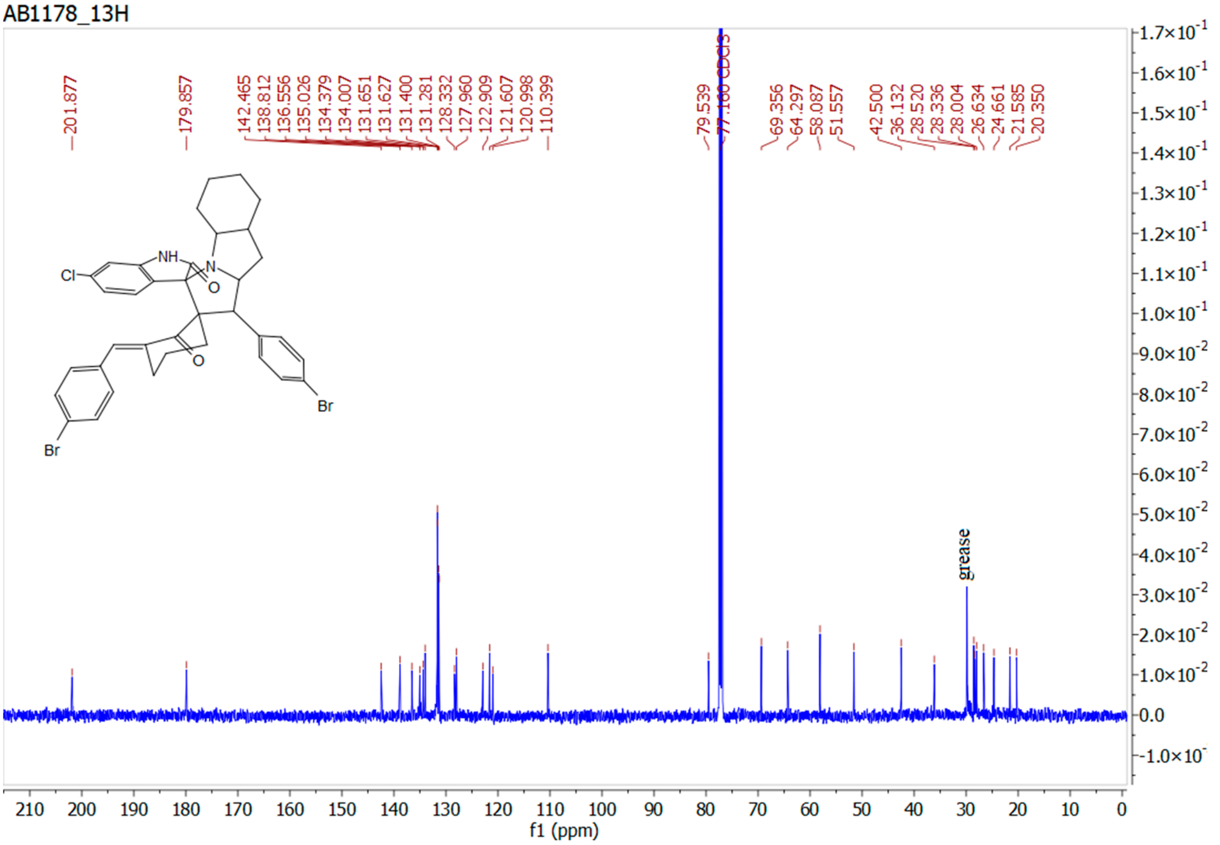

**Figure S7:** $^{1}$H-NMR and $^{13}$C-NMR for compound-**4g**

AB1179_1H

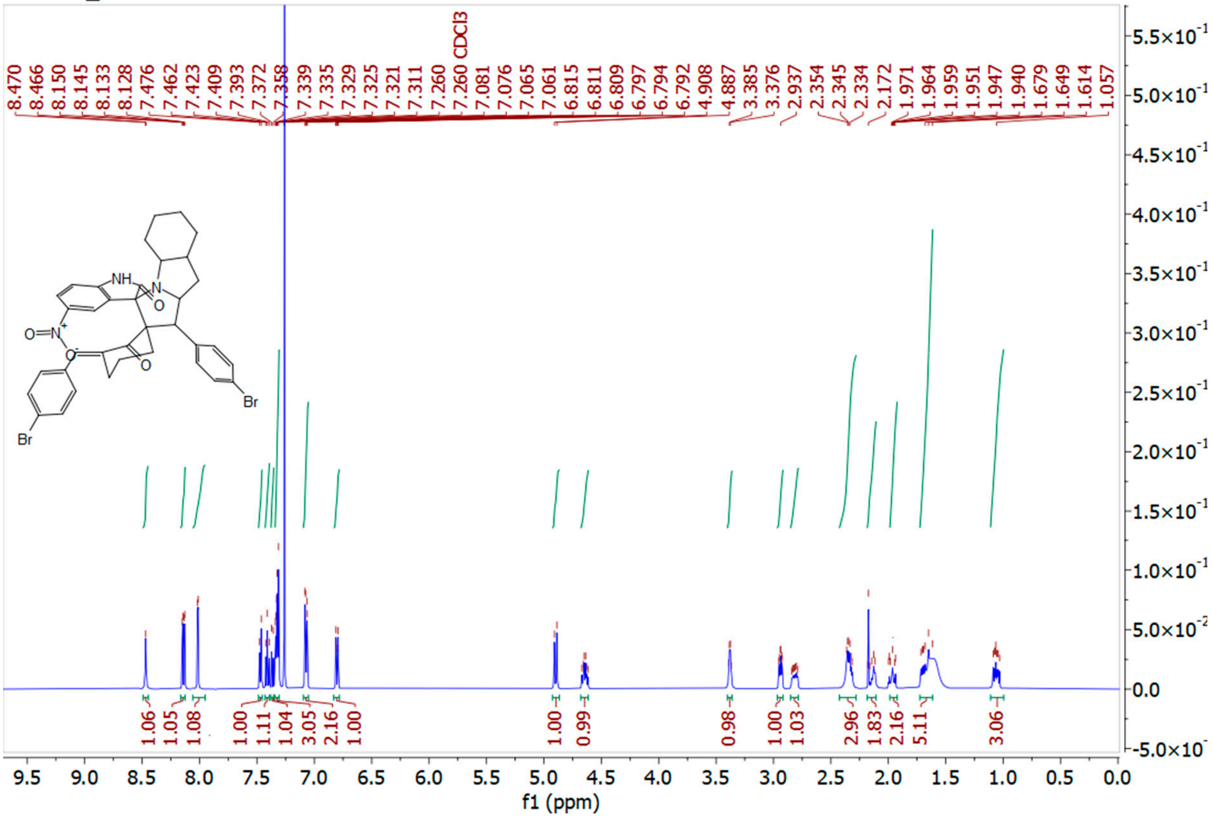


AB1179_13C

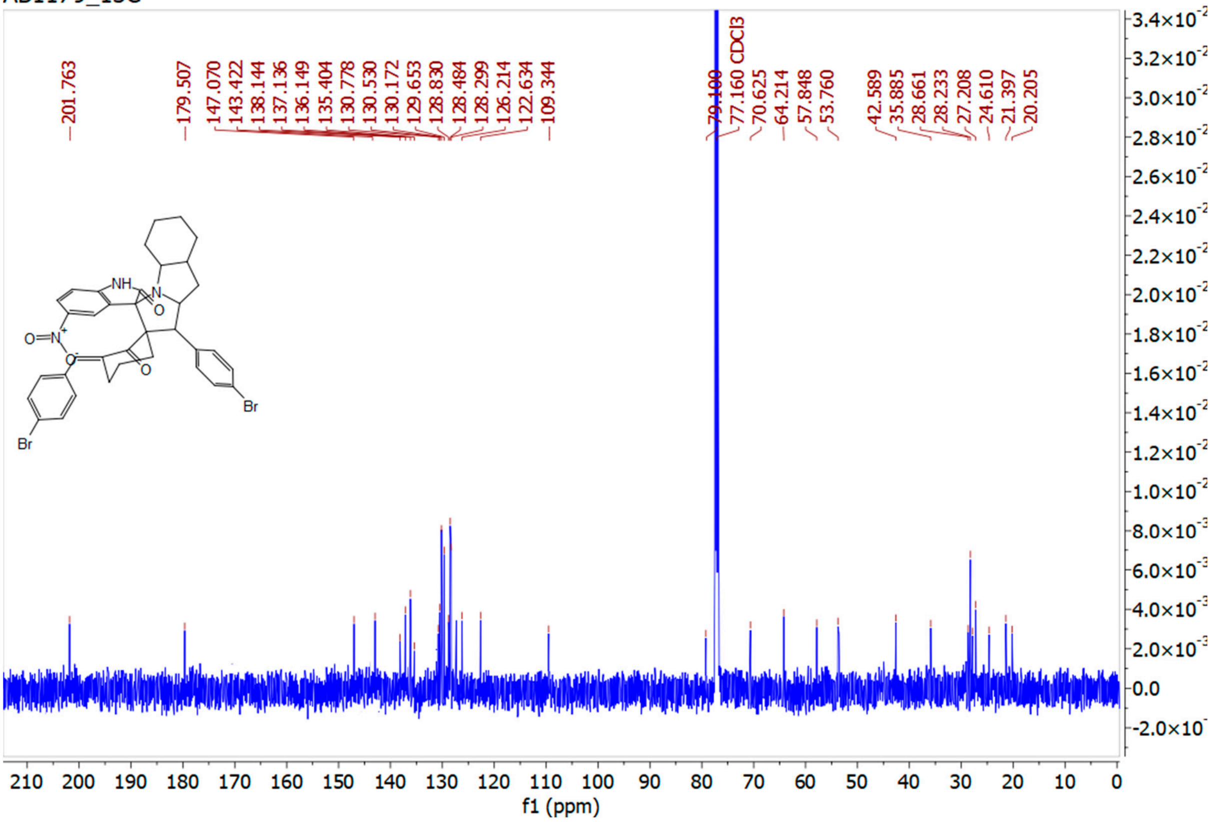

**Figure S8**: $^{1}$H-NMR and $^{13}$C-NMR for compound-**4h**

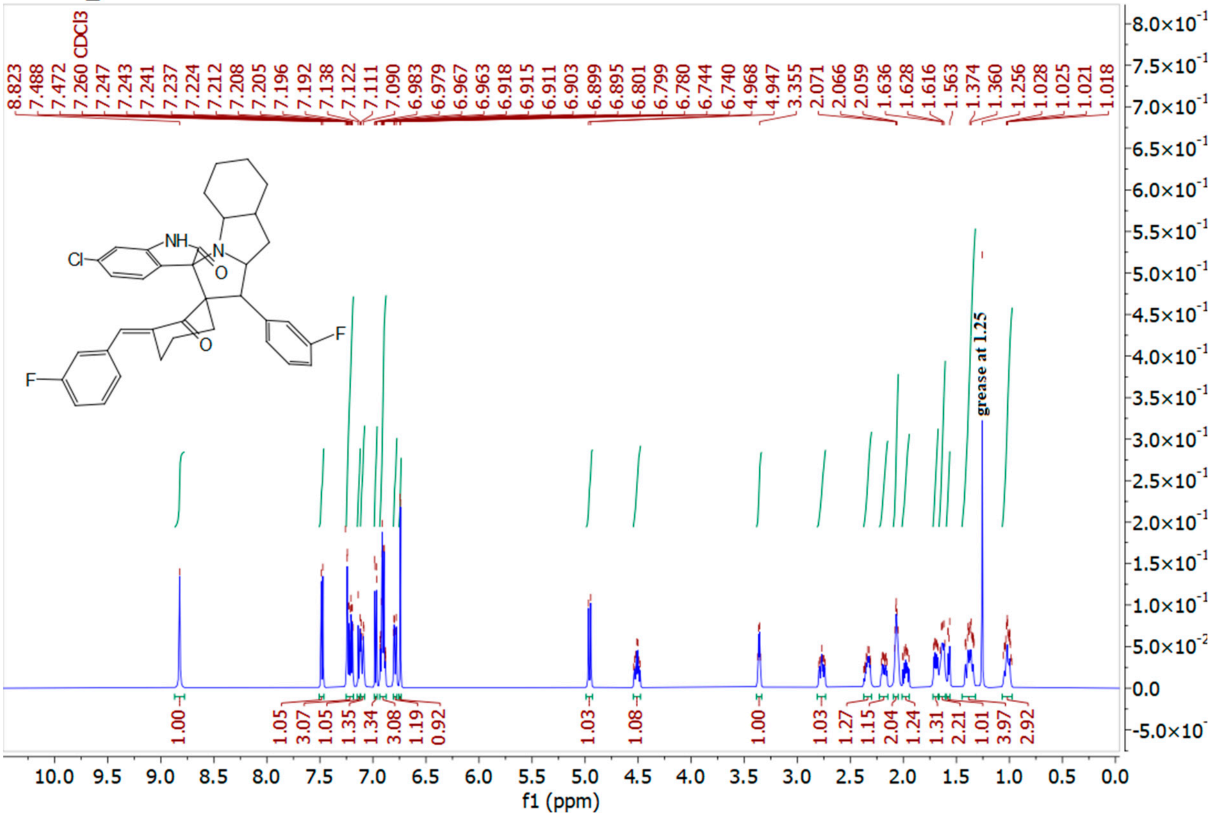


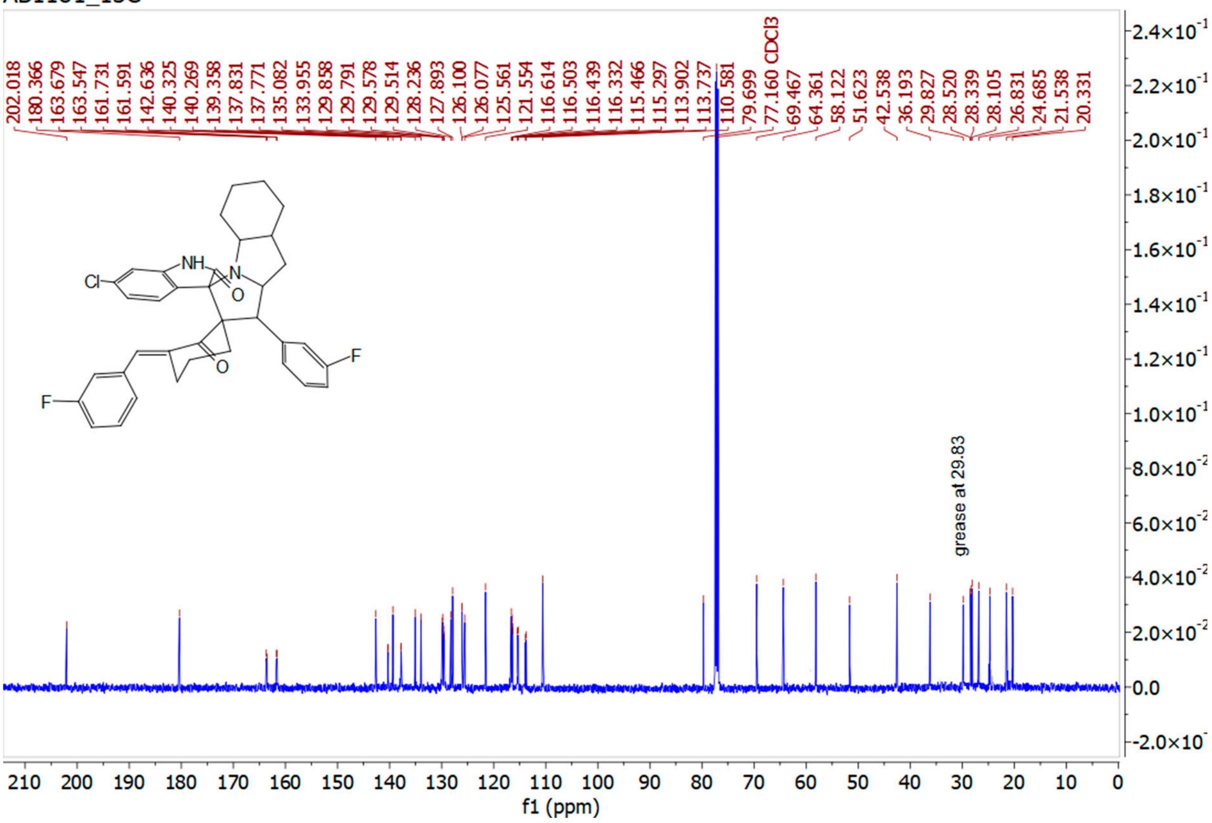

**Figure S9:** $^{1}$H-NMR and $^{13}$C-NMR for compound-**4i**

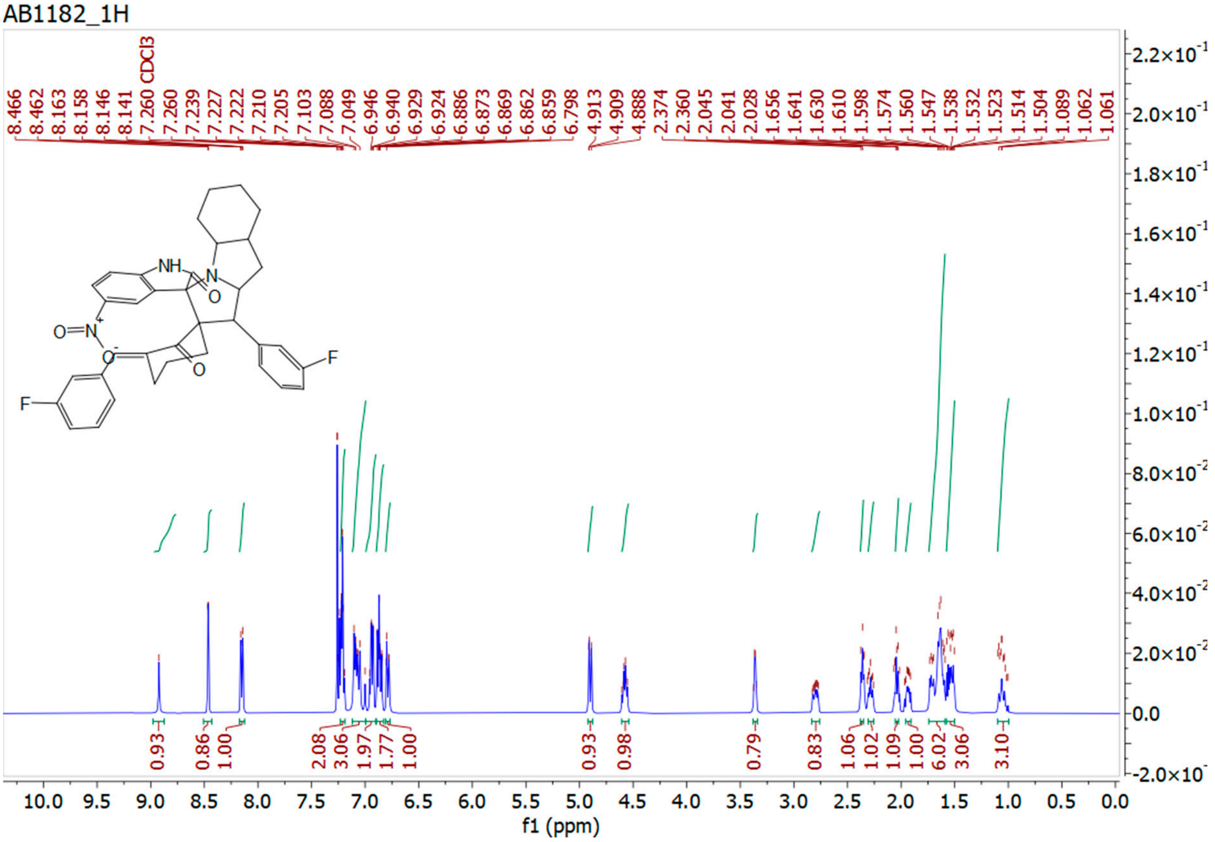


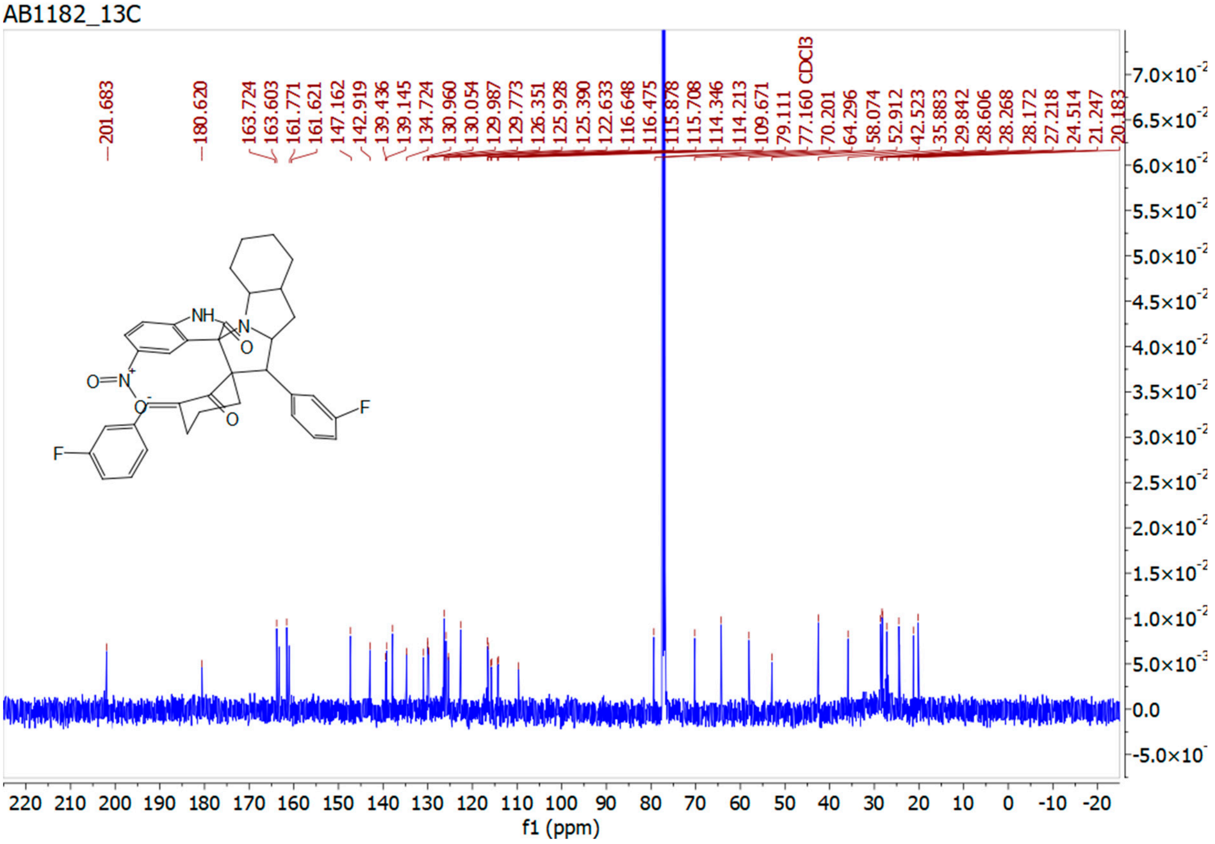

**Figure S10:** $^{1}$H-NMR and $^{13}$C-NMR for Compound-**4j**

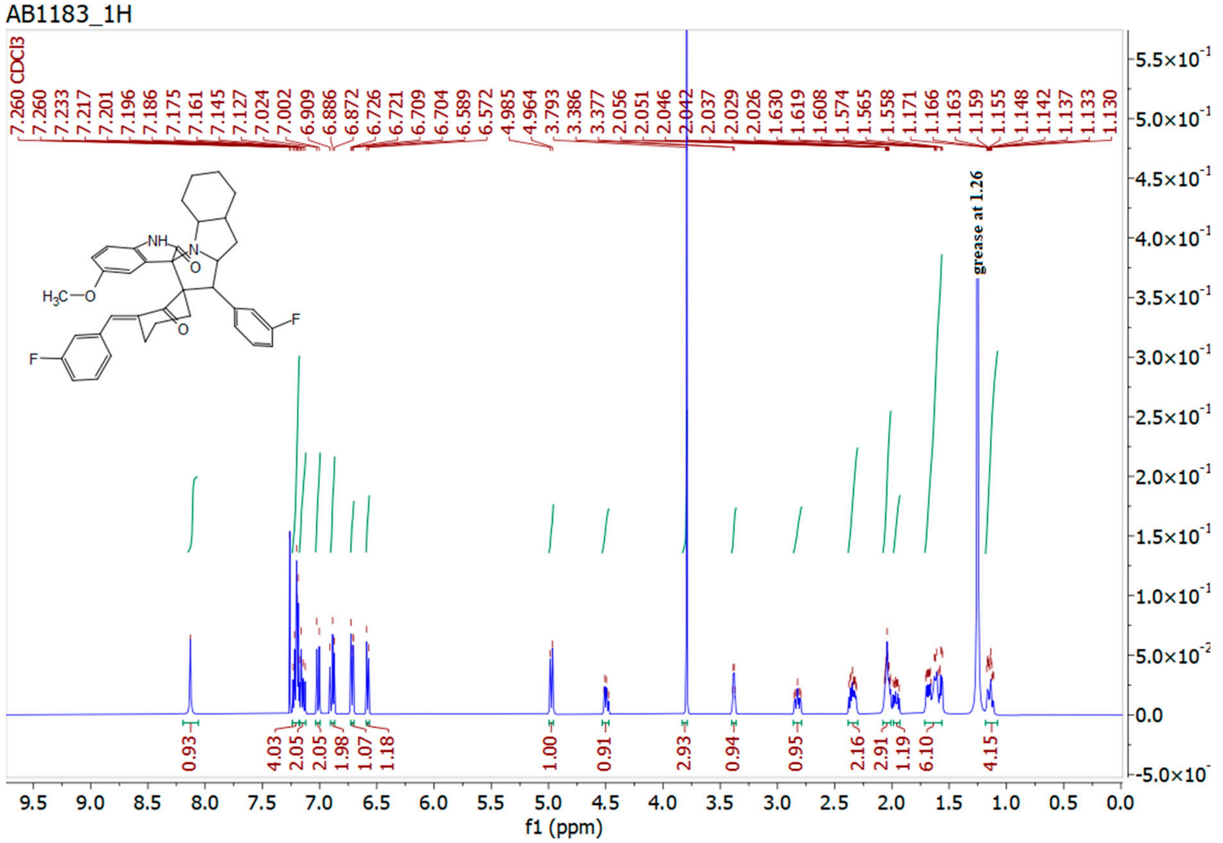


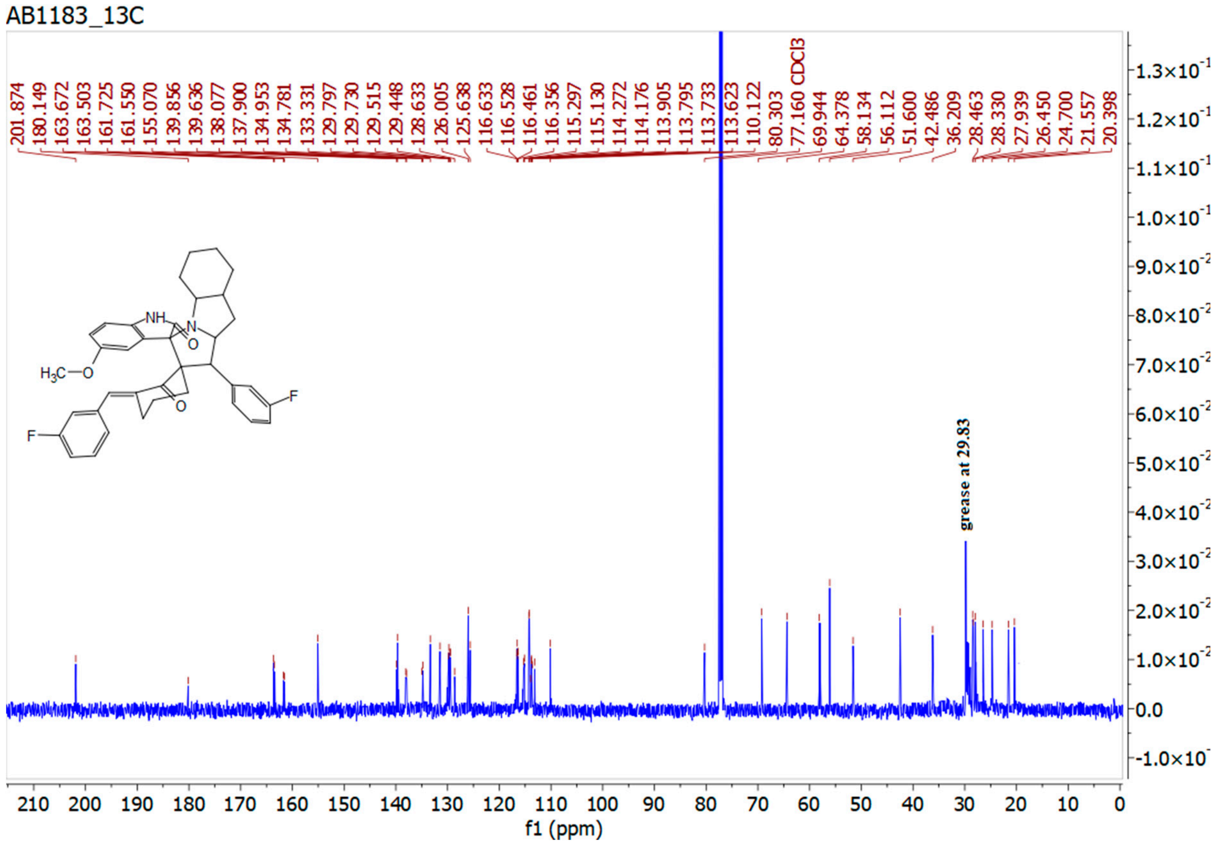

**Figure S11:** $^{1}$H-NMR and $^{13}$C-NMR for compound-**4k**

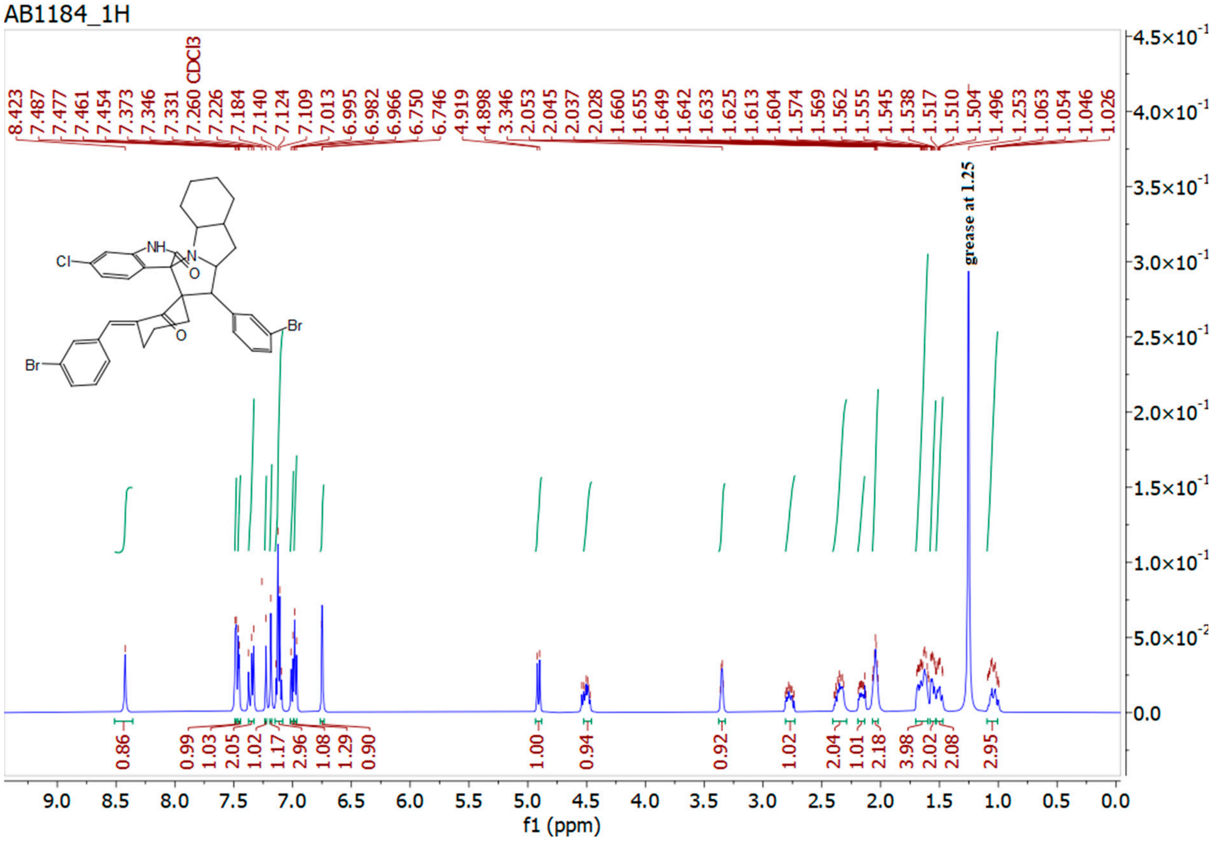


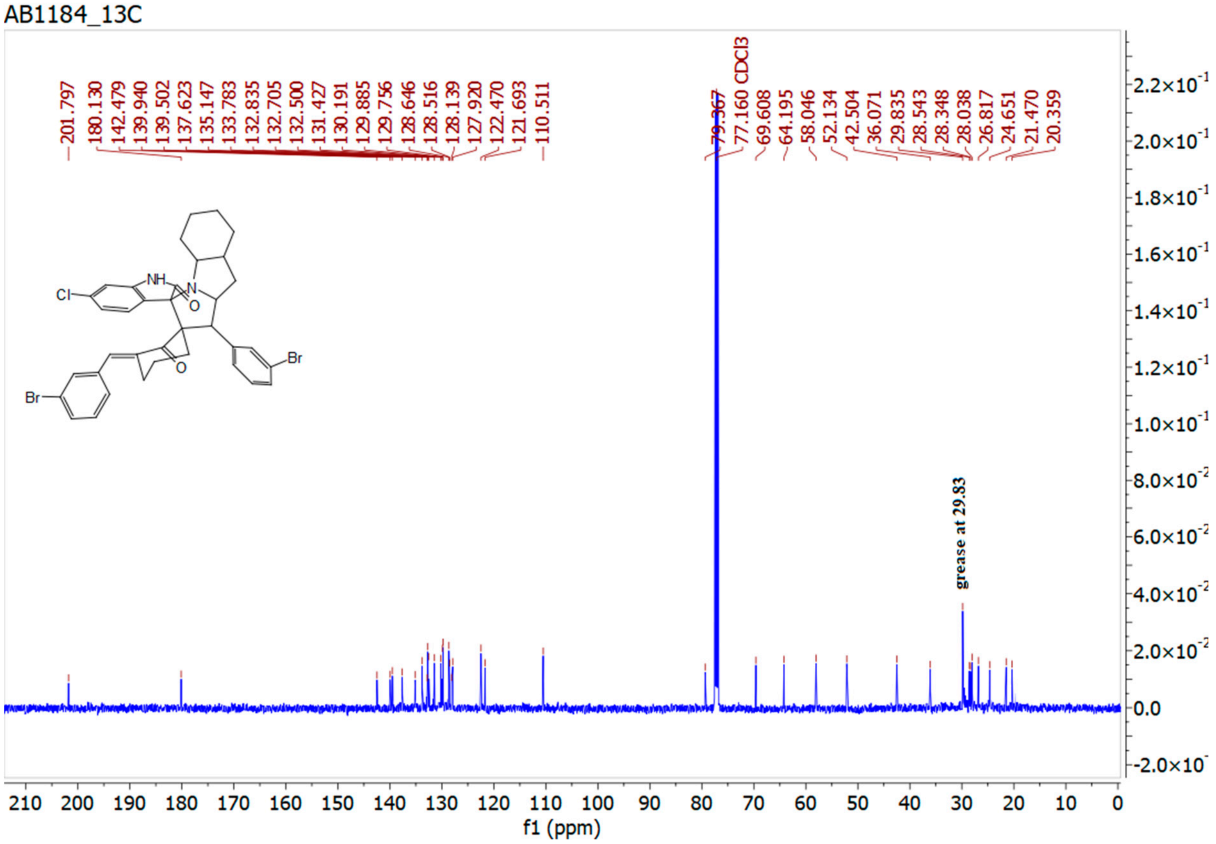

**Figure S12:** $^{1}$H-NMR and $^{13}$C-NMR for compound-**4l**

AB1187_1H

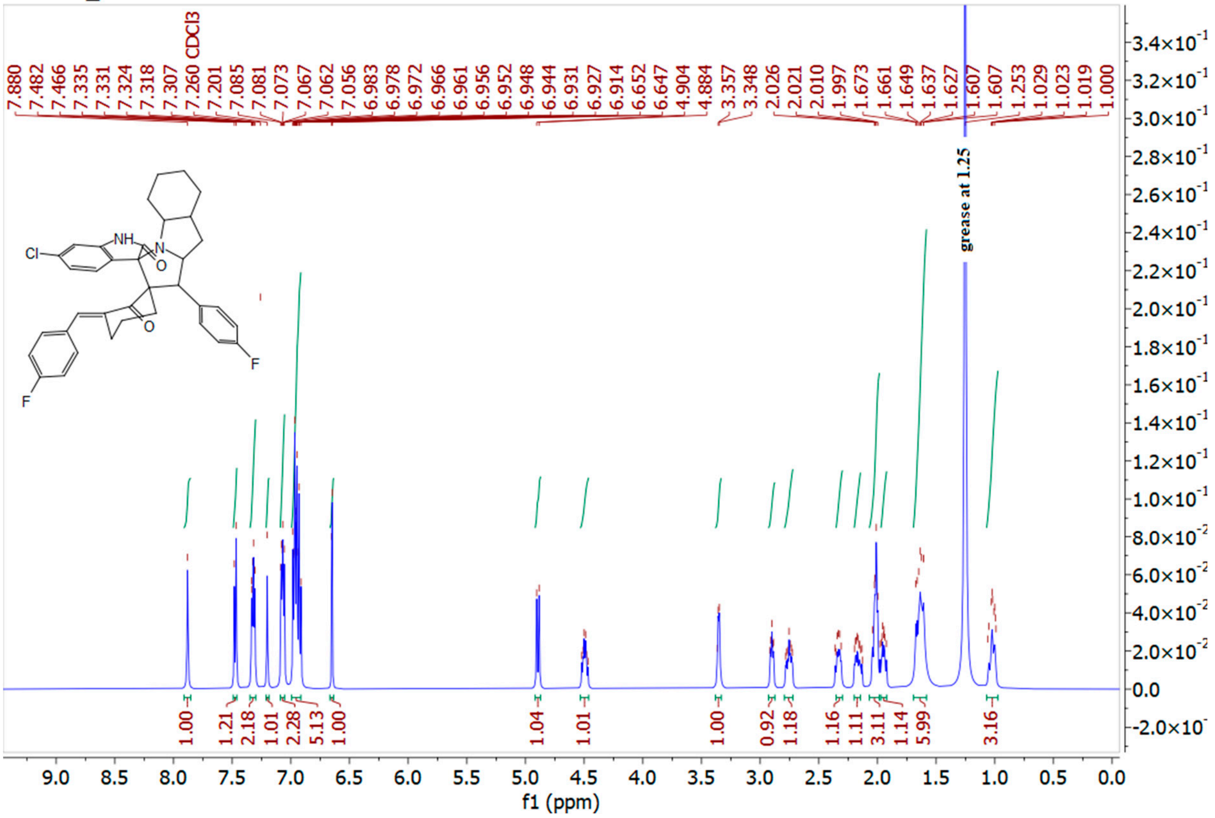

AB1187_13C

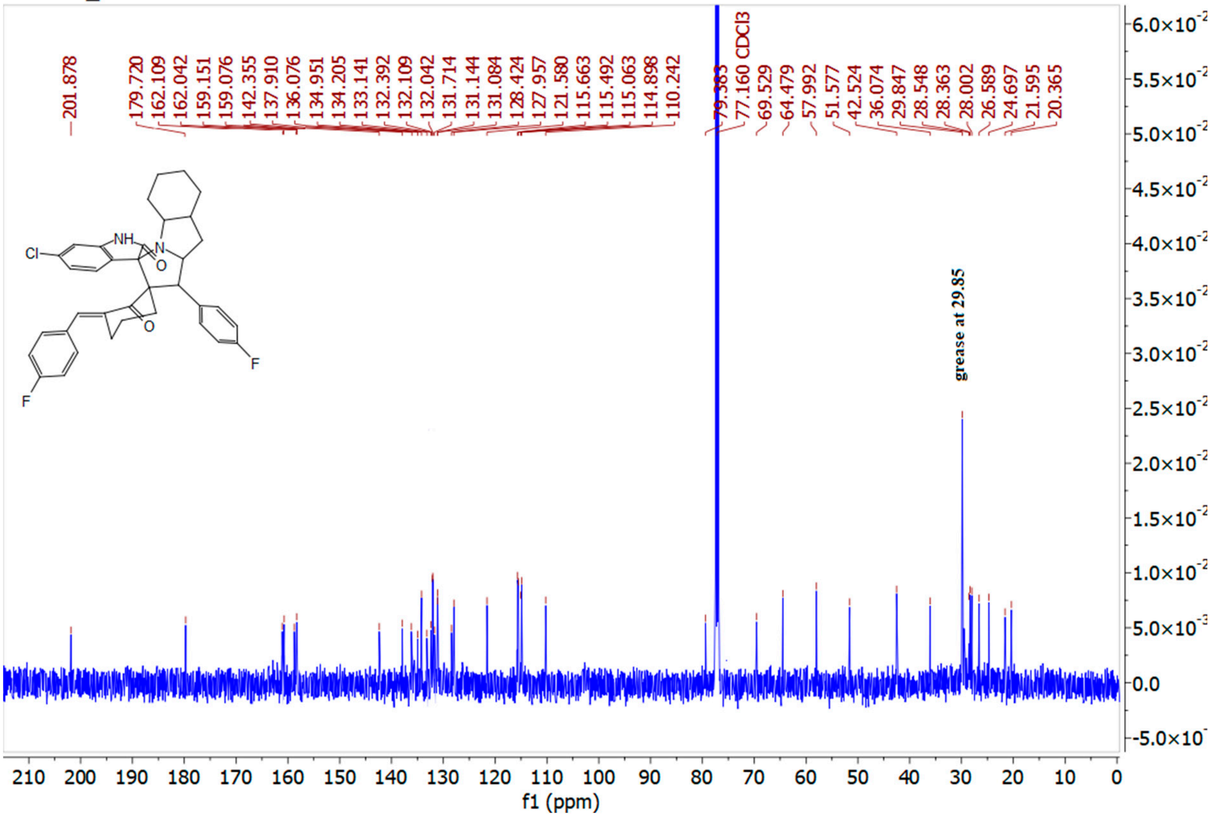

**Figure S13:** $^{1}$H-NMR and $^{13}$C-NMR for compound-**4m**

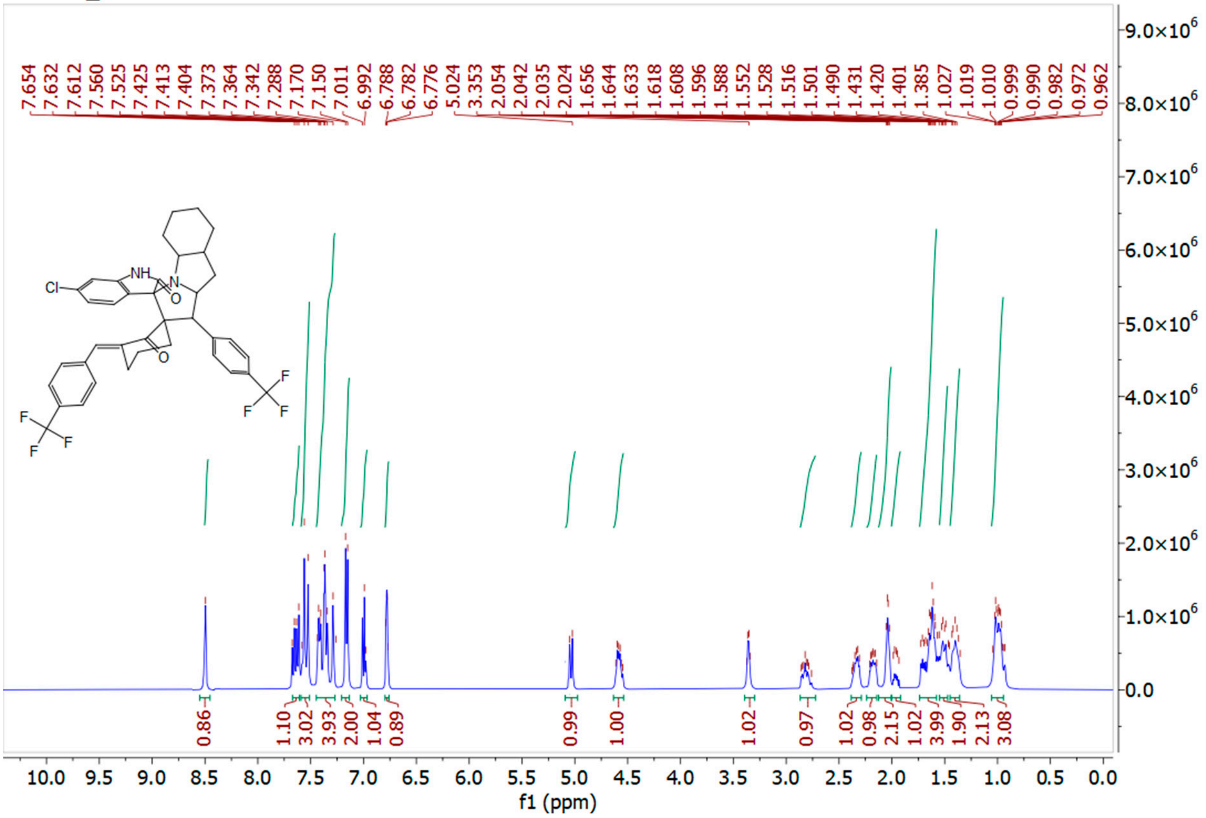


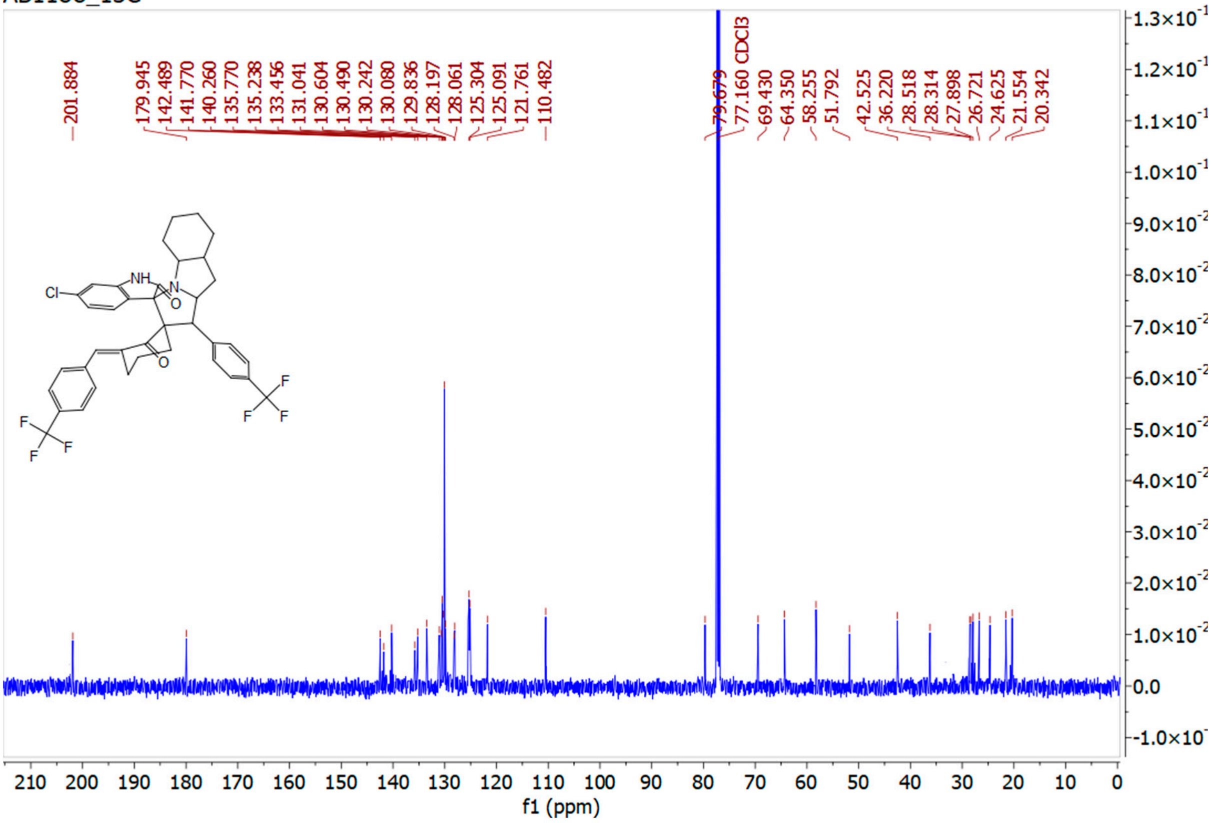

**Figure S14:** $^{1}$H-NMR and $^{13}$C-NMR for compound-**4n**

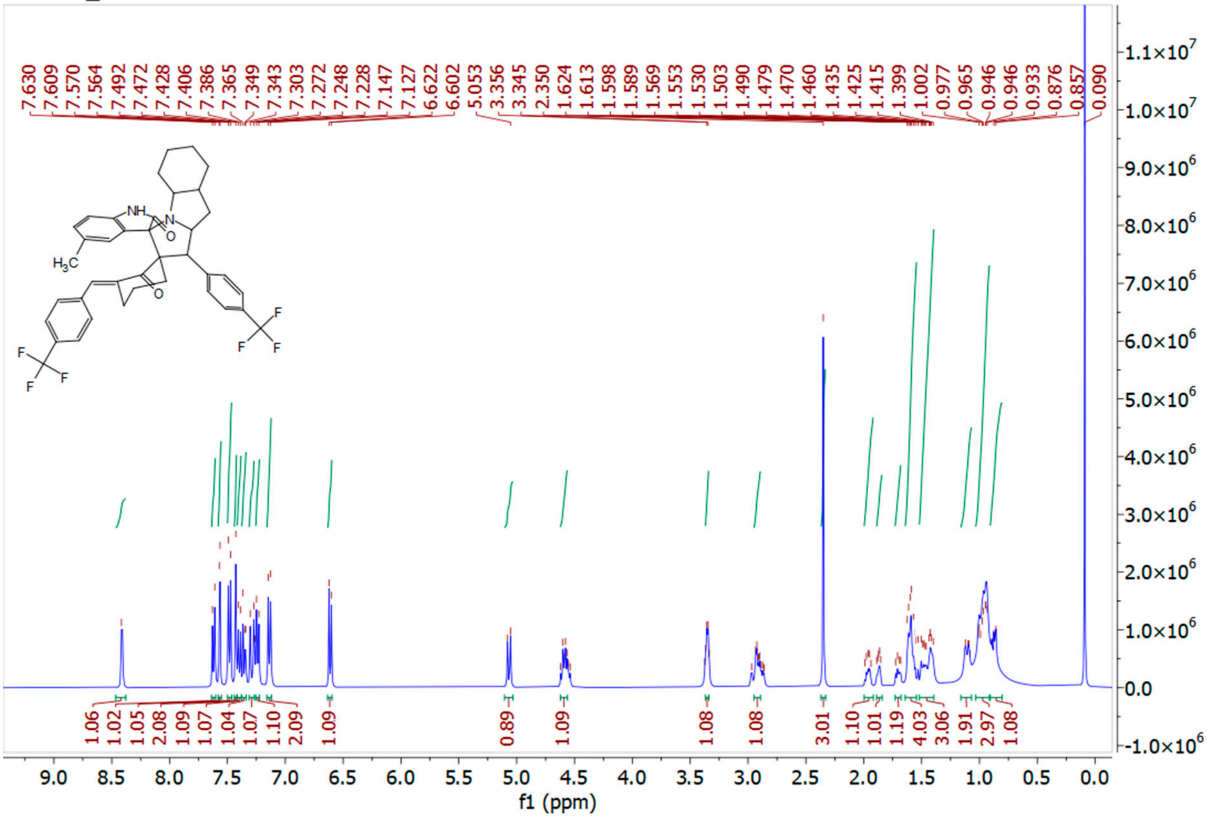


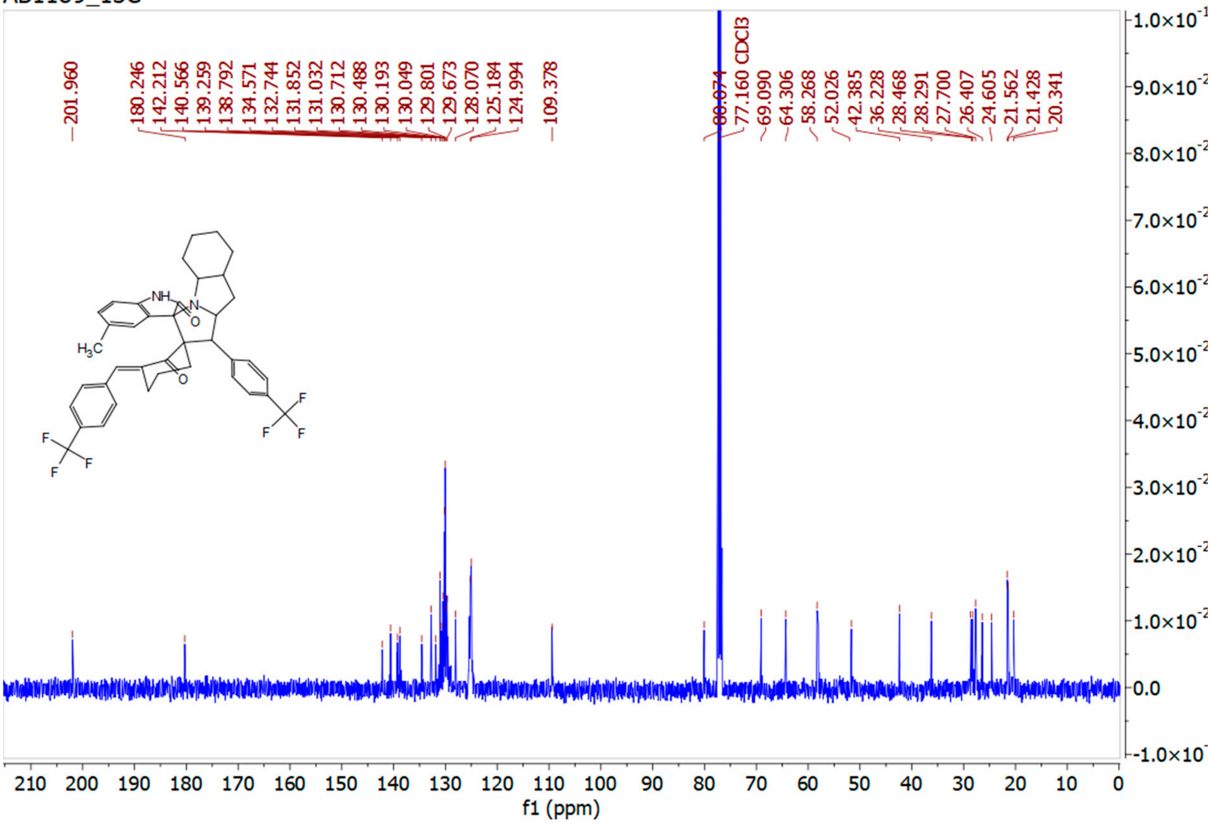

1D- &2D-NMR deep investigation for compound **4f**

**Figure S15:** $^{1}$H-NMR and $^{13}$C-NMR for compound-**4f**

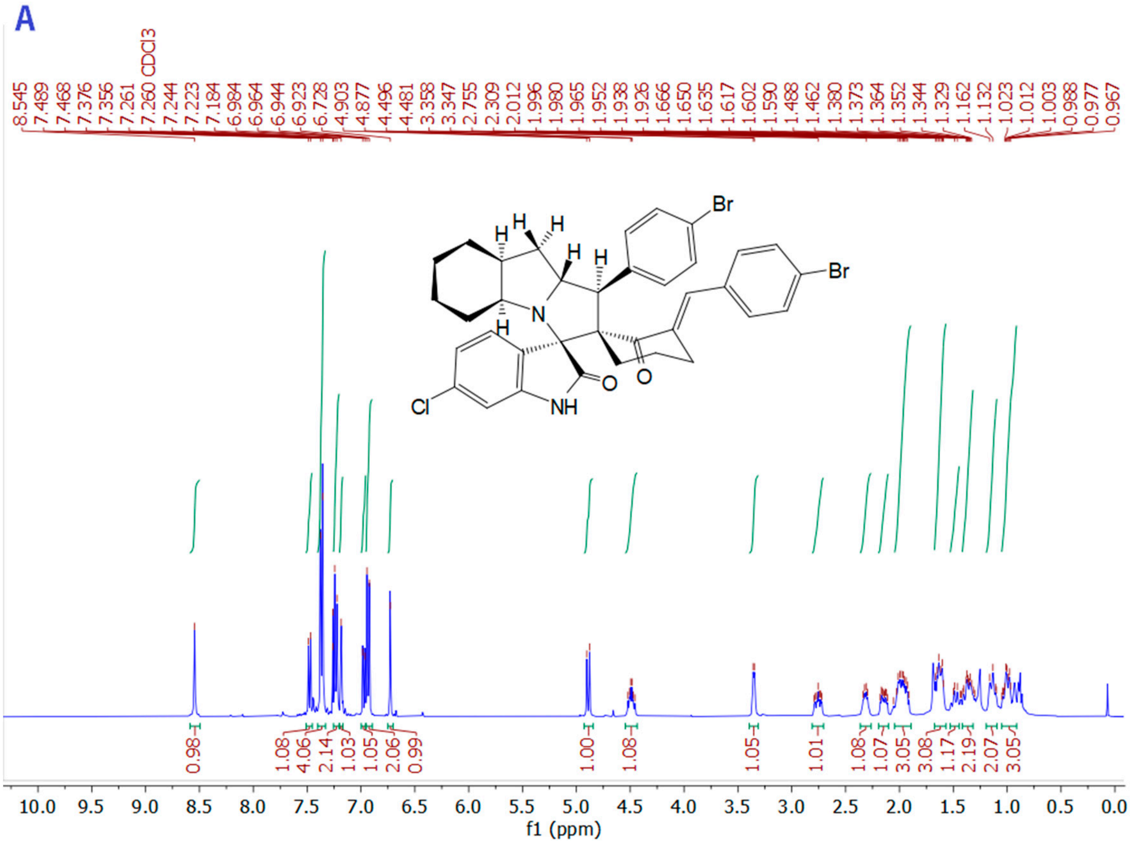

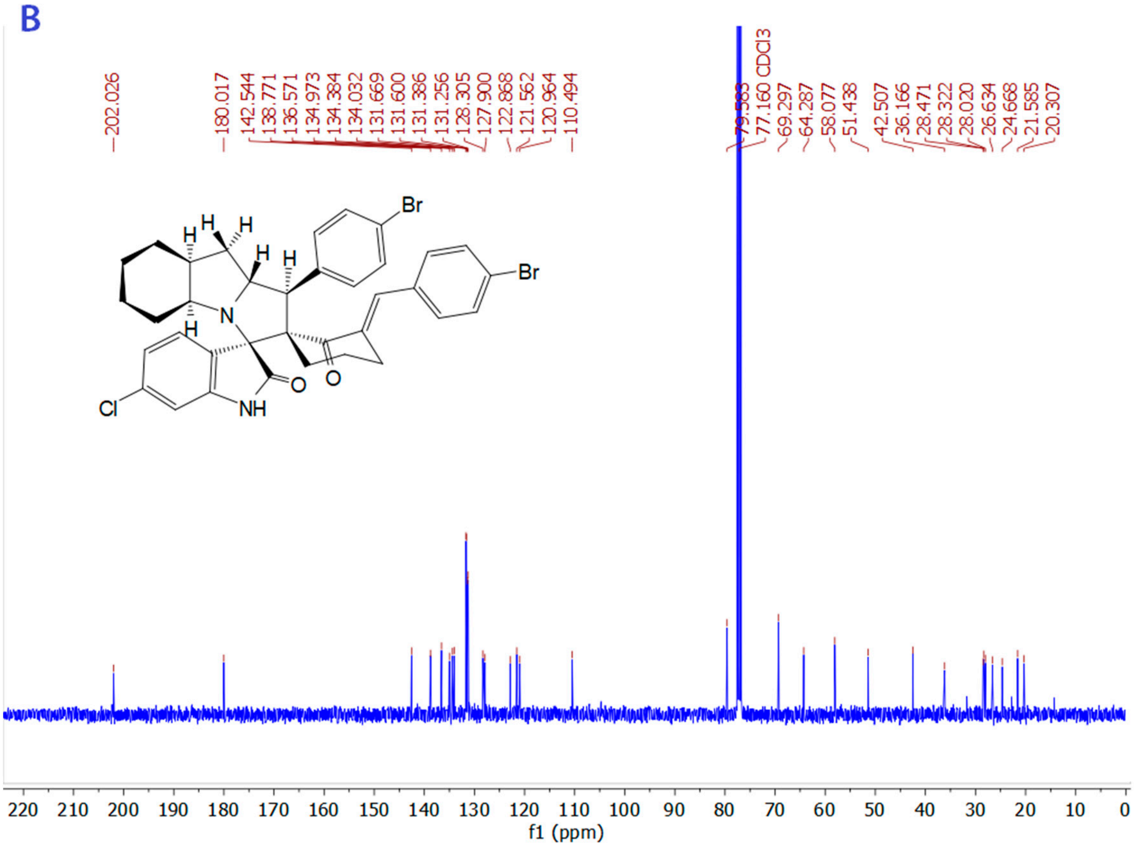

**Figure S16**: DEPT-135- NMR for compound **4f** expanded aliphatic region.

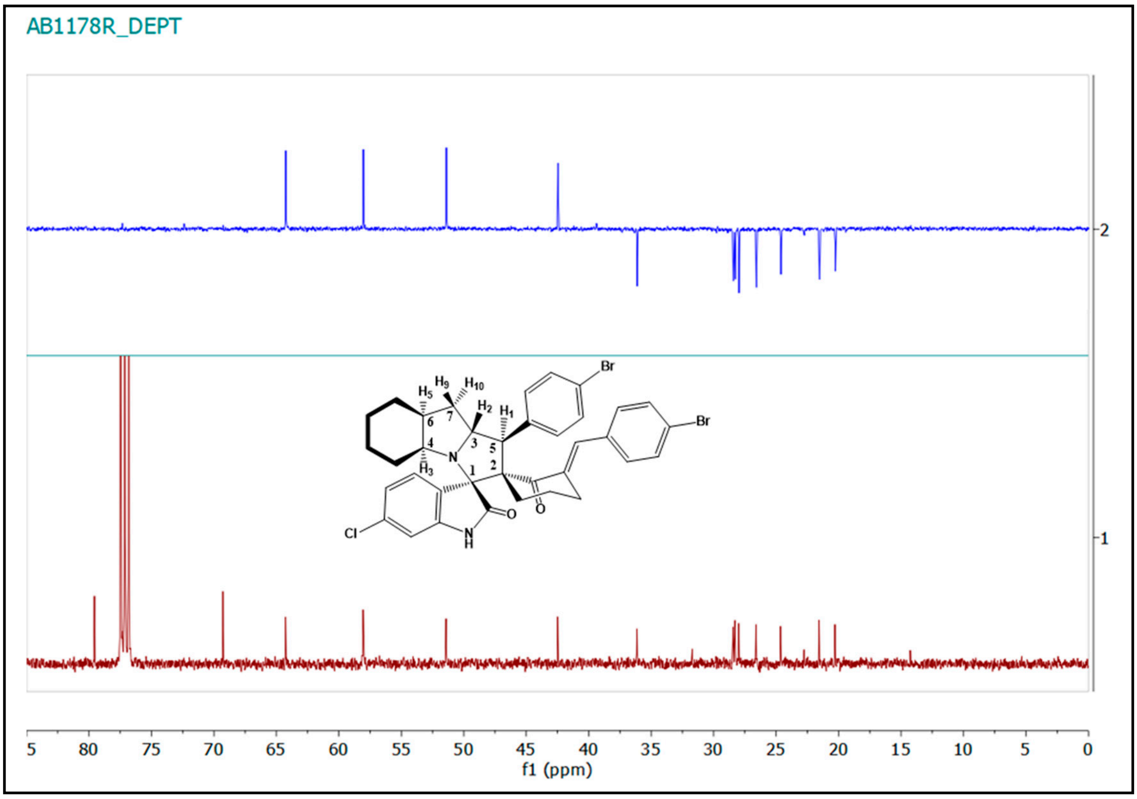

**Figure S17**: HMQC- NMR for compound **4f** more expanded aliphatic region.

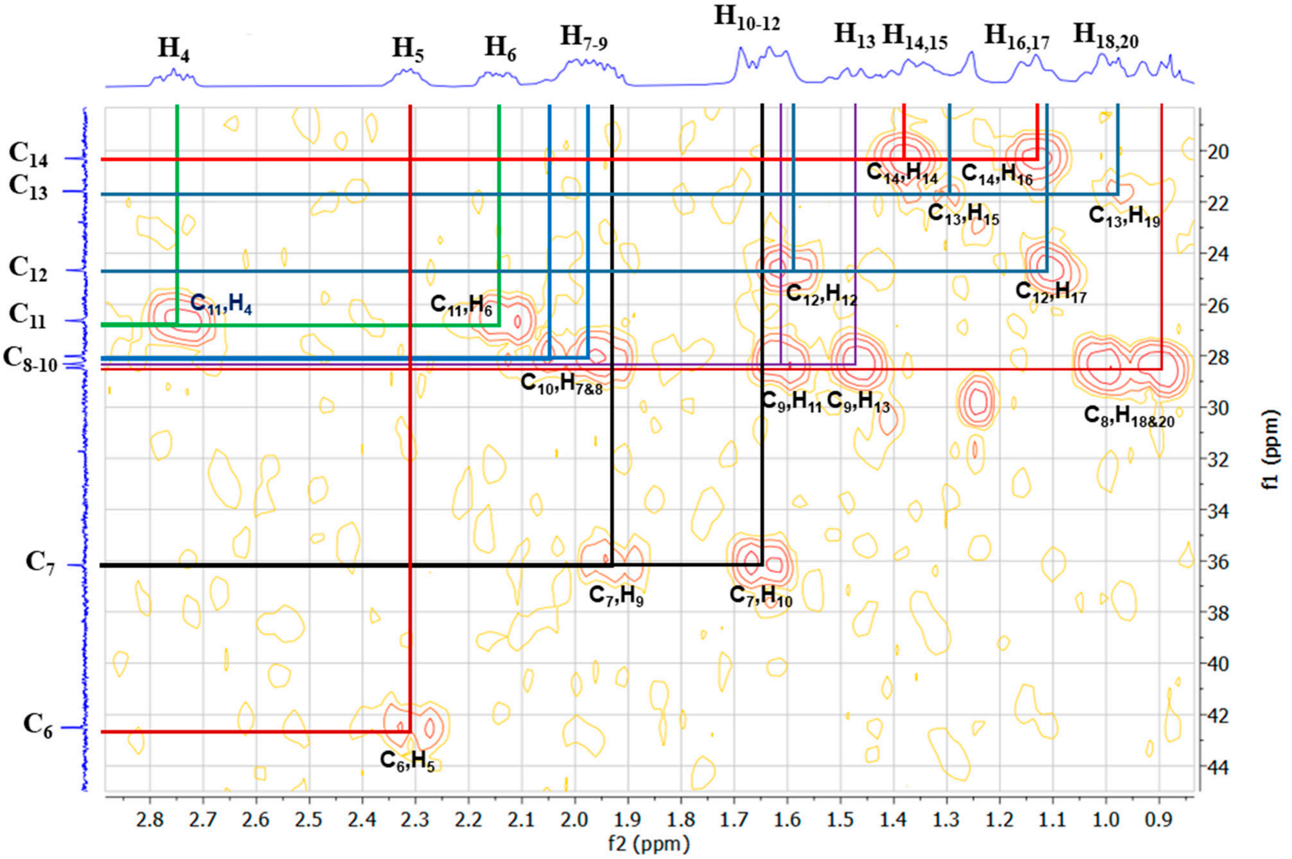


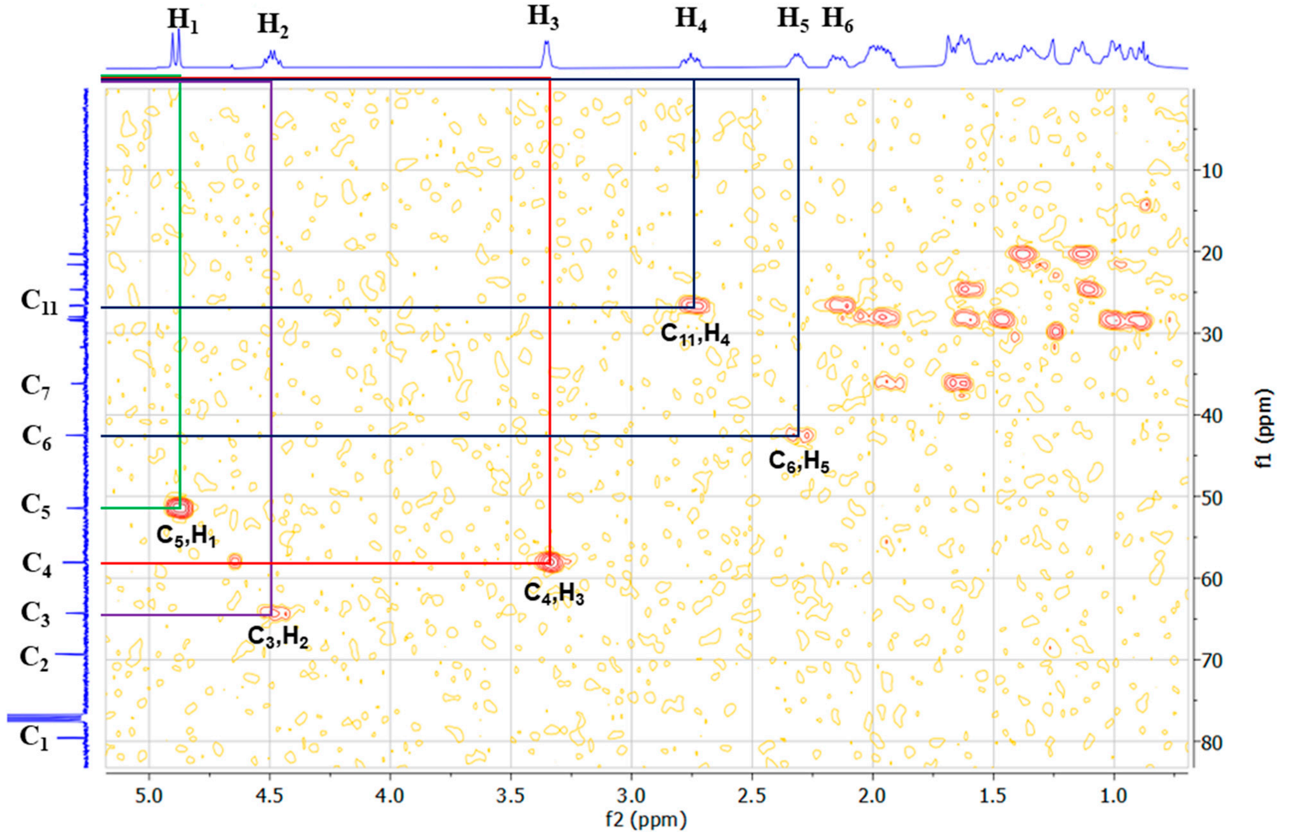

**Figure S18**: COSY- NMR for compound **4f** more expanded aliphatic region.

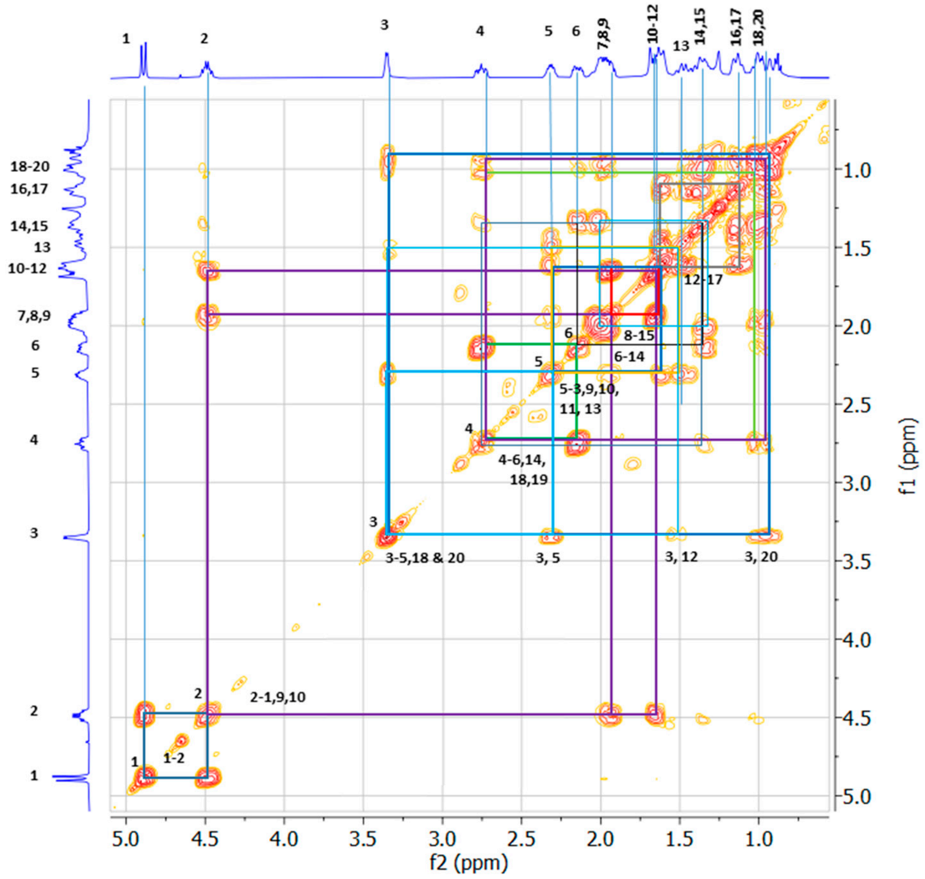


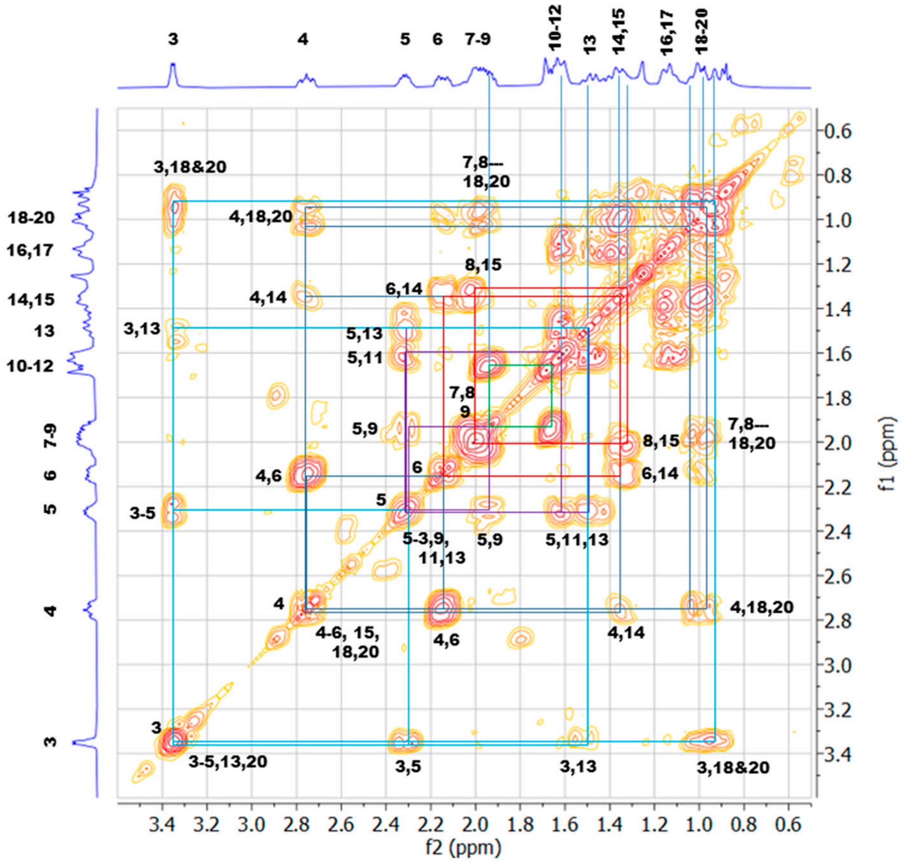

**Figure S19**: HMBC- NMR for compound **4f**.

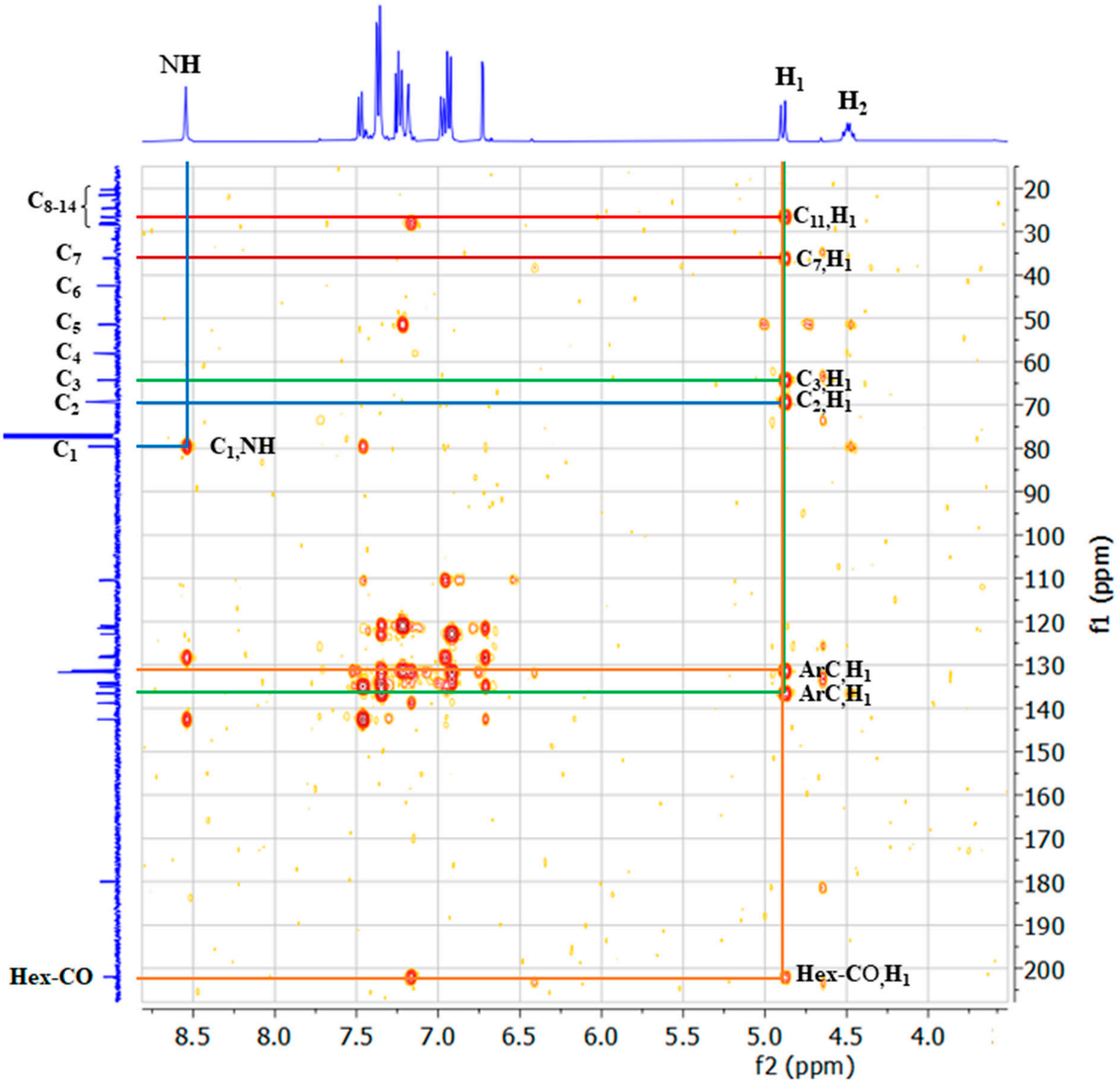


**The Biological Activity Assays Protocols**

Cytotoxic activity of compounds was evaluated in 96-well flat-bottomed micro plates by using the standard MTT (3-[4, 5-dimethylthiazole-2-yl]-2, 5-diphenyl-tetrazolium bromide) colorimetric assay. For this purpose, PC3 cells (Prostate Cancer) / BJ *Human fibroblast* cells / HeLa cells (Cervical Cancer) / MCF-7 and MDA-MB-231 breast cancer cell line were cultured in Dulbecco's Modified Eagle Medium, supplemented with 5% of fetal bovine serum (FBS), 100 IU/ml of penicillin and 100 µg/ml of streptomycin in 75 $cm^2$ flasks, and kept in 5% $CO_2$ incubator at $37^oC$. Exponentially growing cells were harvested, counted with haemocytometer and diluted with a particular medium. Cell culture with the concentration of $1x10^5$ cells/ml was prepared and introduced (100 µL/well) into 96-well plates. After overnight incubation, medium was removed and 200 µL of fresh medium was added with different concentrations of compounds (1-30µM). After 48 hrs, 200 µL MTT (0.5 mg/ml) was added to each well and incubated further for 4 hrs. Subsequently, 100µL of DMSO was added to each well. The extent of MTT reduction to formazan within cells was calculated by measuring the absorbance at 570 nm, using a micro plate reader (Spectra Max plus, Molecular Devices, CA, USA). The cytotoxicity was recorded

as concentration causing 50% growth inhibition ($IC_{50}$) for PC3 cells. The percent inhibition was calculated by using the following formula:

% inhibition = 100-((mean of O.D of test compound – mean of O.D of negative control)/ (mean of O.D of positive control – mean of O.D of negative control)*100).

The results (% inhibition) were processed by using Soft- Max Pro software (Molecular Device, USA).

**STANDARD DRUG:**

Standard drug used in the MTT assay was doxorubicin.

## References


1. Foye, W.O.; Lemke, T.L.; Williams, D.A. *Principles of Medicinal Chemistry*, 4th Ed., Williams and Wilkins, Philadelphia, PA, USA, 2002; pp. 822.
2. Mannerström, M.; Toimela, T.; Sarkanen, J.-R.; Heinonen, T. Human BJ Fibroblasts is an Alternative to Mouse BALB/c 3T3 Cells inIn VitroNeutral Red Uptake Assay. *Basic Clin. Pharmacol. Toxicol.* **2017**, *121*, 109–115, doi:10.1111/bcpt.12790.
3. Price, P.; McMillan1, T.J. Use of the tetrazolium assay in measuring the response of human tumor cells to ionizing radiation. *Cancer Research* **1990**, *50*, 1392–1396. Retrieved from https://cancerres.aacrjournals.org/content/canres/50/5/1392.full.pdf. (access date: 1 June 2020).
4. Scudiero, D. A.; Shoemaker, R.H.; Paull, K.D.; Monks, A.; Tierney, S.; Nofziger, T.H.; Currens, M.J.; Seniff, D.; Boyd, M.R. Evaluation of a soluble tetrazolium/formazan assay for cell growth and drug sensitivity in culture using human and other tumor cell lines. *Cancer Res.* **1988**, *48*, 4827–4833.
5. Mosmann, T. Rapid colorimetric assay for cellular growth and survival: Application to proliferation and cytotoxicity assays. *J. Immunol. Methods* **1983**, *65*, 55–63, doi:10.1016/0022-1759(83)90303-4.
